# Bounding Global Aerosol Radiative Forcing of Climate Change

**DOI:** 10.1029/2019RG000660

**Published:** 2020-03-16

**Authors:** N. Bellouin, J. Quaas, E. Gryspeerdt, S. Kinne, P. Stier, D. Watson‐Parris, O. Boucher, K. S. Carslaw, M. Christensen, A.‐L. Daniau, J.‐L. Dufresne, G. Feingold, S. Fiedler, P. Forster, A. Gettelman, J. M. Haywood, U. Lohmann, F. Malavelle, T. Mauritsen, D. T. McCoy, G. Myhre, J. Mülmenstädt, D. Neubauer, A. Possner, M. Rugenstein, Y. Sato, M. Schulz, S. E. Schwartz, O. Sourdeval, T. Storelvmo, V. Toll, D. Winker, B. Stevens

**Affiliations:** ^1^ Department of Meteorology University of Reading Reading UK; ^2^ Institute for Meteorology Universität Leipzig Leipzig Germany; ^3^ Space and Atmospheric Physics Group Imperial College London London UK; ^4^ Max Planck Institute for Meteorology Hamburg Germany; ^5^ Atmospheric, Oceanic and Planetary Physics, Department of Physics University of Oxford Oxford UK; ^6^ Institut Pierre‐Simon Laplace, Sorbonne Université/CNRS Paris France; ^7^ School of Earth and Environment University of Leeds Leeds UK; ^8^ EPOC, UMR 5805, CNRS‐Université de Bordeaux Pessac France; ^9^ Laboratoire de Météorologie Dynamique/IPSL, CNRS, Sorbonne Université, Ecole Normale Supérieure, PSL Research University, Ecole Polytechnique Paris France; ^10^ NOAA ESRL Chemical Sciences Division Boulder CO USA; ^11^ Priestley International Centre for Climate University of Leeds Leeds UK; ^12^ National Center for Atmospheric Research Boulder CO USA; ^13^ CEMPS University of Exeter Exeter UK; ^14^ UK Met Office Hadley Centre Exeter UK; ^15^ Institute for Atmospheric and Climate Science ETH Zürich Zürich Switzerland; ^16^ Department of Meteorology Stockholm University Stockholm Sweden; ^17^ Center for International Climate and Environmental Research‐Oslo (CICERO) Oslo Norway; ^18^ Department of Global Ecology Carnegie Institution for Science Stanford CA USA; ^19^ Now at Institute for Atmospheric and Environmental Sciences Goethe University Frankfurt Germany; ^20^ Department of Applied Energy, Graduate School of Engineering, Nagoya University Nagoya Japan; ^21^ Now at Faculty of Science, Department of Earth and Planetary Sciences Hokkaido University Sapporo Japan; ^22^ Climate Modelling and Air Pollution Section, Research and Development Department Norwegian Meteorological Institute Oslo Norway; ^23^ Brookhaven National Laboratory Environmental and Climate Sciences Department Upton NY USA; ^24^ Laboratoire d'Optique Atmosphérique Université de Lille Villeneuve d'Ascq France; ^25^ Department of Geosciences University of Oslo Oslo Norway; ^26^ Now at Institute of Physics University of Tartu Tartu Estonia; ^27^ NASA Langley Research Center Hampton VA USA; ^28^ Now at Institut für Geophysik und Meteorologie Universität zu Köln Köln Germany

## Abstract

Aerosols interact with radiation and clouds. Substantial progress made over the past 40 years in observing, understanding, and modeling these processes helped quantify the imbalance in the Earth's radiation budget caused by anthropogenic aerosols, called aerosol radiative forcing, but uncertainties remain large. This review provides a new range of aerosol radiative forcing over the industrial era based on multiple, traceable, and arguable lines of evidence, including modeling approaches, theoretical considerations, and observations. Improved understanding of aerosol absorption and the causes of trends in surface radiative fluxes constrain the forcing from aerosol‐radiation interactions. A robust theoretical foundation and convincing evidence constrain the forcing caused by aerosol‐driven increases in liquid cloud droplet number concentration. However, the influence of anthropogenic aerosols on cloud liquid water content and cloud fraction is less clear, and the influence on mixed‐phase and ice clouds remains poorly constrained. Observed changes in surface temperature and radiative fluxes provide additional constraints. These multiple lines of evidence lead to a 68% confidence interval for the total aerosol effective radiative forcing of ‐1.6 to ‐0.6 W m^−2^, or ‐2.0 to ‐0.4 W m^−2^ with a 90% likelihood. Those intervals are of similar width to the last Intergovernmental Panel on Climate Change assessment but shifted toward more negative values. The uncertainty will narrow in the future by continuing to critically combine multiple lines of evidence, especially those addressing industrial‐era changes in aerosol sources and aerosol effects on liquid cloud amount and on ice clouds.

## Key Points

- An assessment of multiple lines of evidence supported by a conceptual model provides ranges for aerosol radiative forcing of climate change
- Aerosol effective radiative forcing is assessed to be between ‐1.6 and ‐0.6 W m^−2^ at the 16–84% confidence level
- Although key uncertainties remain, new ways of using observations provide stronger constraints for models

## 1 Introduction

At steady state and averaged over a suitably long period, the heat content in the Earth system, defined here as the ocean, the atmosphere, the land surface, and the cryosphere, remains constant because incoming radiative fluxes balance their outgoing counterparts. Perturbations to the radiative balance force the state of the system to change. Those perturbations can be natural, for example, due to variations in the astronomical parameters of the Earth, a change in solar radiative output or injections of gases and aerosol particles by volcanic eruptions. Perturbations can also be due to human activities, which change the composition of the atmosphere.

A key objective of Earth system sciences is to understand historical changes in the energy budget of the Earth over the industrial period (Myhre et al., 2017) and how they translate into changes in the state variables of the atmosphere, land, and ocean; to attribute observed temperature change since preindustrial times to specific perturbations (Jones et al., 2016); and to predict the impact of projected emission changes on the climate system. From that understanding climate scientists can derive estimates of the amount of committed warming that can be expected from past emissions (Pincus & Mauritsen, 2017; Schwartz, 2018), estimates of net carbon dioxide emissions that would be consistent with maintaining the increase in global mean surface temperature below agreed targets (Allen et al., 2018), or the efficacy of climate engineering to possibly mitigate against climate changes in the future (Kravitz et al., 2015).

**Figure 1:**
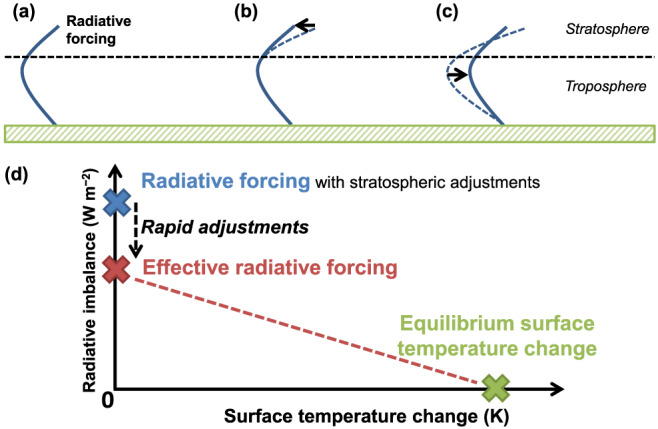
(a) Instantaneous radiative forcing: A perturbation is applied, but the vertical profiles of temperature (solid line) and moisture remain unperturbed. (b) Stratosphere‐adjusted radiative forcing: Stratospheric temperatures respond (transition from dashed to solid line). (c) Effective radiative forcing: The perturbation also triggers rapid adjustments in the troposphere, but surface temperatures have not yet responded. (d) The system returns to radiative balance by a change in surface temperature.

A sustained radiative perturbation imposed on the climate system initially exerts a transient imbalance in the energy budget, which is called a radiative forcing (RF; denoted as F; Figure 1a). The system then responds by eventually reaching a new steady state whereby its heat content once again remains fairly constant. The equilibrium change in global mean surface temperature Δ*T*_s_, in K, is given by

(1) $$\Delta T_{s}=\lambda \;F$$

where F is the global mean RF, in W m^−2^, and *λ* is the climate sensitivity parameter that quantifies the combined effect of feedbacks, in K (W m^−2^)^−1^ (Ramanathan, 1975). For multiple reasons, including lack of knowledge of *λ* and the long response time of *T*_s_ to RF (Forster, 2016; Knutti et al., 2017; Schwartz, 2012), it has become customary to compare the strengths of different perturbations by their RFs rather than by the changes in *T*_s_ that ultimately ensue.

Temperatures in the stratosphere, a region of the atmosphere which is largely uncoupled from the troposphere‐land‐ocean system below, respond on a timescale of months, adjusting the magnitude and in the case of ozone perturbations even the sign of the initial RF (Figure 1b) (Hansen et al., 1997). This adjusted RF is defined by the 5th Assessment Report (AR5) of the Intergovernmental Panel on Climate Change (IPCC) (Myhre, Shindell, et al., 2013) as the change in net downward radiative flux at the tropopause, holding tropospheric state variables fixed at their unperturbed state but allowing for stratospheric temperatures to adjust to radiative equilibrium. This definition is adopted by this review.

In addition to exerting a RF, changes in atmospheric composition affect other global mean quantities, such as temperature, moisture, surface radiative and heat fluxes, and wind fields, as well as their spatiotemporal patterns. Some of these responses occur on timescales much faster than the adjustment timescales of ocean surface temperatures. These responses are called rapid adjustments and occur independently of surface temperature change (Hansen et al., 2005; Shine et al., 2003). Rapid adjustment mechanisms can augment or offset the initial RF by a sizable fraction, because they involve changes to the radiative properties of the atmosphere, including clouds, and/or the surface, which all contribute substantially to the Earth's energy budget. Consequently, effective radiative forcing (ERF; denoted E; Figure 1c), which is the sum of RF and the associated rapid adjustments, is a better predictor of Δ*T*_s_ than RF (Figure 1d). Sherwood et al. (2015) make a pedagogical presentation of the concept of rapid adjustments that was used in IPCC AR5 (Boucher et al., 2013; Myhre, Shindell, et al., 2013). This review also adopts the definition of ERF introduced in the IPCC AR5 (Boucher et al., 2013; Myhre, Shindell, et al., 2013), which is the change in net top‐of‐atmosphere downward radiative flux that includes adjustments of temperatures, water vapor, and clouds throughout the atmosphere, including the stratosphere, but with sea surface temperature maintained fixed. In addition to its influence on global temperature change, ERF is also an efficient predictor of changes in globally averaged precipitation rate (Andrews et al., 2010). Those changes arise from a balance between radiative changes within the atmosphere and changes in the latent and sensible heat fluxes at the surface (Richardson et al., 2016). Accounting for rapid adjustments when quantifying radiative changes is essential to obtain the full response of precipitation.

**Table 1:** Best Estimates and Uncertainty Ranges of Radiative Forcing of Aerosol‐Radiation and Aerosol‐Cloud Interactions, and Total Aerosol Radiative Forcing, in W m^−2^, as Given by Successive Assessment Reports of the IPCC

Assessment	Forcing	Aerosol‐radiation	Aerosol‐cloud	Total
report	period	interactions	interactions	
2 (Schimel et al., 1996)	1750–1993	−0.50 (−1.00 to −0.25)	N/A (−1.5 to 0.0)	N/A
3 (Penner et al., 2001)	1750–1998	N/A	N/A (−2 to 0.0)	N/A
4 (Forster et al., 2007)	1750–2005	−0.50 (−0.90 to −0.10)	−0.70 (−1.80 to −0.30)	−1.3 (−2.2 to −0.5)
5 Boucher et al. (2013)	1750–2011	−0.45 (−0.95 to +0.05)	−0.45 (−1.2 to 0.0)	−0.9 (−1.9 to −0.1)

*Note*. Uncertainty ranges are given at the 90% confidence level. The First Assessment Report did not have the scientific understanding needed to quantify aerosol radiative forcing, although they noted that it was potentially substantial. All values are for radiative forcing, except for the Fifth Assessment Report, which are for effective radiative forcing. Adapted from Table 8.6 of (Myhre, Shindell, et al., 2013).

RF can be induced in multiple ways: changes in atmospheric composition, both in the gaseous and particulate phases, induced by volcanic or anthropogenic emissions; changes in surface albedo; and variations in solar irradiance. An estimated full range of anthropogenic aerosol RF based on an elicitation of 24 experts of −0.3 W m^−2^ to −2.1 W m^−2^ at the 90% confidence level was presented by Morgan et al. (2006). Individual experts, however, allowed for the possibility of much more negative, but also the possibility even of net positive, RF. A similar degree of uncertainty has been reflected in an evolving series of IPCC assessment reports (Table 1), where best estimates and uncertainty ranges of aerosol RF are also based at least partly on expert judgment. Since RFs are additive within the forcing‐response paradigm, the uncertainty attached to the aerosol ERF translates to the entire anthropogenic ERF (Schwartz & Andreae, 1996). Recognition of this fact has motivated a tremendous effort, now lasting several decades, to better understand how aerosols influence radiation, clouds, and ultimately the large‐scale trajectory of the climate system, involving field measurements, laboratory studies, and modeling from microphysical to global scales (e.g., Ghan & Schwartz, 2007; Kulmala et al., 2011; Seinfeld et al., 2016).

In spring 2018, under the auspices of the World Climate Research Programme's Grand Science Challenge on Clouds, Circulation and Climate Sensitivity, 36 experts gathered at Schloss Ringberg, in the mountains of Southern Germany, to take a fresh and comprehensive look at the present state of understanding of aerosol ERF and identify prospects for progress on some of the most pressing open questions, thereby drawing the outlines for this review. The participants at that workshop expressed a wide range of views regarding the mechanisms and magnitudes of aerosol influences on the Earth's energy budget. This review represents a synthesis of these views and the underlying evidence.

This review is structured as follows. Section 2 reviews the physical mechanisms by which anthropogenic aerosols exert an RF of climate and sets the scope of this review. Section 3 presents a conceptual model of globally averaged aerosol ERF and the different lines of evidence used to quantify the uncertainty bounds in the terms of that conceptual model. Section 4 quantifies changes in aerosol amounts between preindustrial and present‐day conditions. Sections 5 and 6 review current knowledge of aerosol interactions with radiation and clouds, respectively, to propose bounds for their RF, while sections 7 and 8, respectively, do the same for their rapid adjustments. Section 9 reviews the knowledge, and gaps thereof, in aerosol‐cloud interactions in ice clouds. Section 10 reviews estimates of aerosol ERF based on the response of the climate system over the last century. Finally, section 11 brings all lines of evidence together to bound total global aerosol ERF and outlines open questions and research directions that could further contribute to narrow uncertainty or reduce the likelihood of surprises.

## 2 Mechanisms, Scope, and Terminology

### 2.1 Aerosol RF Mechanisms

The term “atmospheric aerosol” denotes a suspension of microscopic and submicroscopic particles in air. These particles may be primary, meaning emitted directly in the liquid or solid phase, or secondary, meaning that they are produced in the atmosphere from gaseous precursors. In both cases, sources may be natural, for example, sand storms, sea spray, volcanoes, natural wildfires, and biogenic emissions, or result from human activities, like construction and cement production, agriculture, and combustion of biomass and fossil fuels (Hoesly et al., 2018). Once in the atmosphere, aerosols undergo microphysical (e.g., coagulation and condensation) and chemical (e.g., oxidation) transformation and are transported with the atmospheric flow. Tropospheric aerosols, the aerosols of main concern here, remain in the atmosphere for days to weeks (e.g., Kristiansen et al., 2012). Those relatively short residence times, compared to greenhouse gases, are caused by efficient removal processes, either by direct deposition to the surface by sedimentation, diffusion, or turbulence or by scavenging by and into cloud droplets and ice crystals, and subsequent precipitation. As a consequence of these relatively rapid removal processes together with spatially heterogeneous distribution of sources, tropospheric aerosols are highly nonuniform spatially and temporally: A mean residence time of approximately 5 days results in typical transport distances of about 2000 km. In consequence, aerosols are concentrated in and downwind of source regions such as cities and industrialized regions. In contrast, aerosols introduced into the stratosphere, for example, by explosive volcanic eruptions, may have residence times of several months to a few years because of slow particle sedimentation velocities and secondary aerosol production.

**Figure 2:**
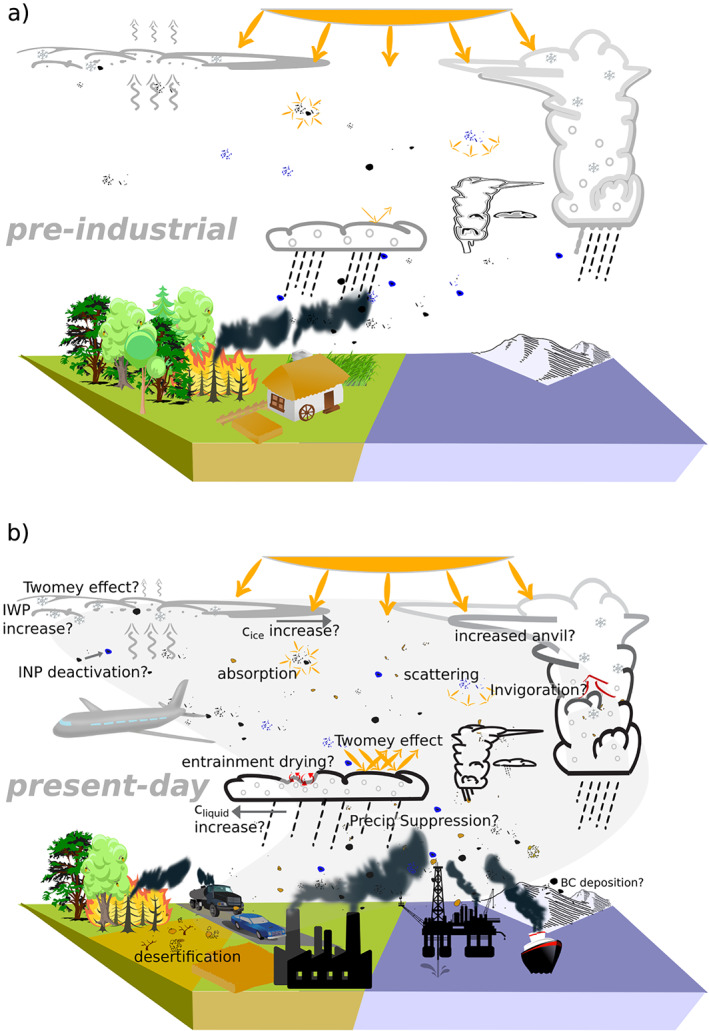
Simplified representation of the impact of anthropogenic aerosol emissions on the Earth system in (a) the preindustrial and (b) the present‐day atmosphere. A schematic representation of known processes relevant for the effective radiative forcing of anthropogenic aerosol is summarized for present‐day conditions in panel (b), but the same processes were active, with different strengths, in preindustrial conditions. Processes where the impact on the effective radiative forcing remains qualitatively uncertain are followed by a question mark. $C_{\text{liquid}}$ and $C_{\text{ice}}$ denote liquid and ice cloud fractions, respectively. LWP and IWP stand for liquid and ice water path, respectively. INP stands for ice nucleating particle.

Aerosols modify the Earth's radiative budget directly through scattering and absorption of radiation, denoted here aerosol radiative interaction, ari, and indirectly by modifying the microphysical properties of clouds, affecting their reflectivity and persistence, denoted here aerosol‐cloud interactions, aci (Figure 2). Aerosols may also affect the reflectivity of the surface, as absorbing aerosol deposited on snow‐covered surfaces may decrease their reflectivity. As a result of these processes, anthropogenic emissions of aerosols and their gaseous precursors have over the Anthropocene exerted an ERF, which is thought to have been strengthening over time for much of the industrial period, but is locally and instantaneously highly variable. All of this heterogeneity combines to make the aerosol ERF challenging to quantify, not just locally, but also in the global and annual mean.

Aerosol‐radiation interactions are readily discerned by human observers as smoke, haze, and dust (see box). As early as the fifteenth century, Leonardo da Vinci in instructions on how to paint a battle scene noted that the distribution of light in a mineral dust and biomass burning plume was such that “*from the side whence the light comes this mixture of air and smoke and dust will seem far brighter than on the opposite side*” (Paris Manuscript A, circa 1492), a manifestation of the angular distribution of light scattering that must be accurately represented in calculation of the RF. Volcanic aerosols and their impact on sunsets have also influenced a wide range of artists as shown by Zerefos et al. (2014). In this context the possibility that anthropogenic and volcanic aerosols decrease atmospheric transmittance of solar radiation globally was therefore considered relatively early in climate change studies (e.g., McCormick & Ludwig, 1967; Mitchell, 1971).

Improvements in the physical understanding of atmospheric scattering and absorption, combined with a good constraint on ocean surface reflectance, allowed Haywood et al. (1999) to show that ari was needed to explain satellite‐retrieved top‐of‐atmosphere shortwave radiative fluxes under cloud‐free conditions. Aerosol contributions to outgoing shortwave radiative fluxes can exceed 100 W m^−2^ in some cases, as estimated for example, by Haywood et al. (2003) from aircraft measurements of a mineral dust plume over the ocean. In addition to these direct effects of scattering and absorption, rapid adjustments to ari, originally called semidirect effects, were postulated by Grassl (1975), then again more recently from global modeling (Hansen et al., 1997), and observations made during the Indian Ocean Experiment (INDOEX) field campaign (Ackerman et al., 2000). Those adjustments stem from changes in the distribution of atmospheric radiative fluxes and heating rates induced by the aerosols, especially light‐absorbing aerosols, which then modify surface radiative and heat fluxes, temperature and water vapor profiles, atmospheric stability, and the conditions for cloud formation (Stjern et al., 2017). Correlations between satellite retrievals of aerosol, clouds, and planetary albedo consistent with the expected signature of semidirect effects have been reported, for example, over the subtropical South Atlantic Ocean (Wilcox, 2012) and North Atlantic marine stratocumulus decks (Amiri‐Farahani et al., 2017).

**“Impact of absorption on aerosol‐radiation interactions”**

Aerosol particles scatter and absorb solar (also called shortwave) and terrestrial (or longwave) radiation, hereafter denoted aerosol‐radiation interaction (ari). The efficiency at which they do so depends on the wavelength of the radiation, the distribution of particle sizes, their shapes, and on their refractive index, which is determined by their chemical composition and mixing state (Hansen & Travis, 1974). For each particle, both scattering and absorption contribute to the extinction of radiation, and the single‐scattering albedo (SSA), denoted *ϖ*_0_, quantifies the contribution of scattering to total extinction:

(2) $$\varpi_{0}=\frac{\sigma_{\text{sca}}}{\sigma_{\text{sca}}+\sigma_{\text{abs}}}$$

where *σ*_sca_ and *σ*_abs_ are the scattering and absorption cross sections, respectively, in units of area. This key quantity can be likewise defined for a population of aerosol particles.

**Figure 3:**
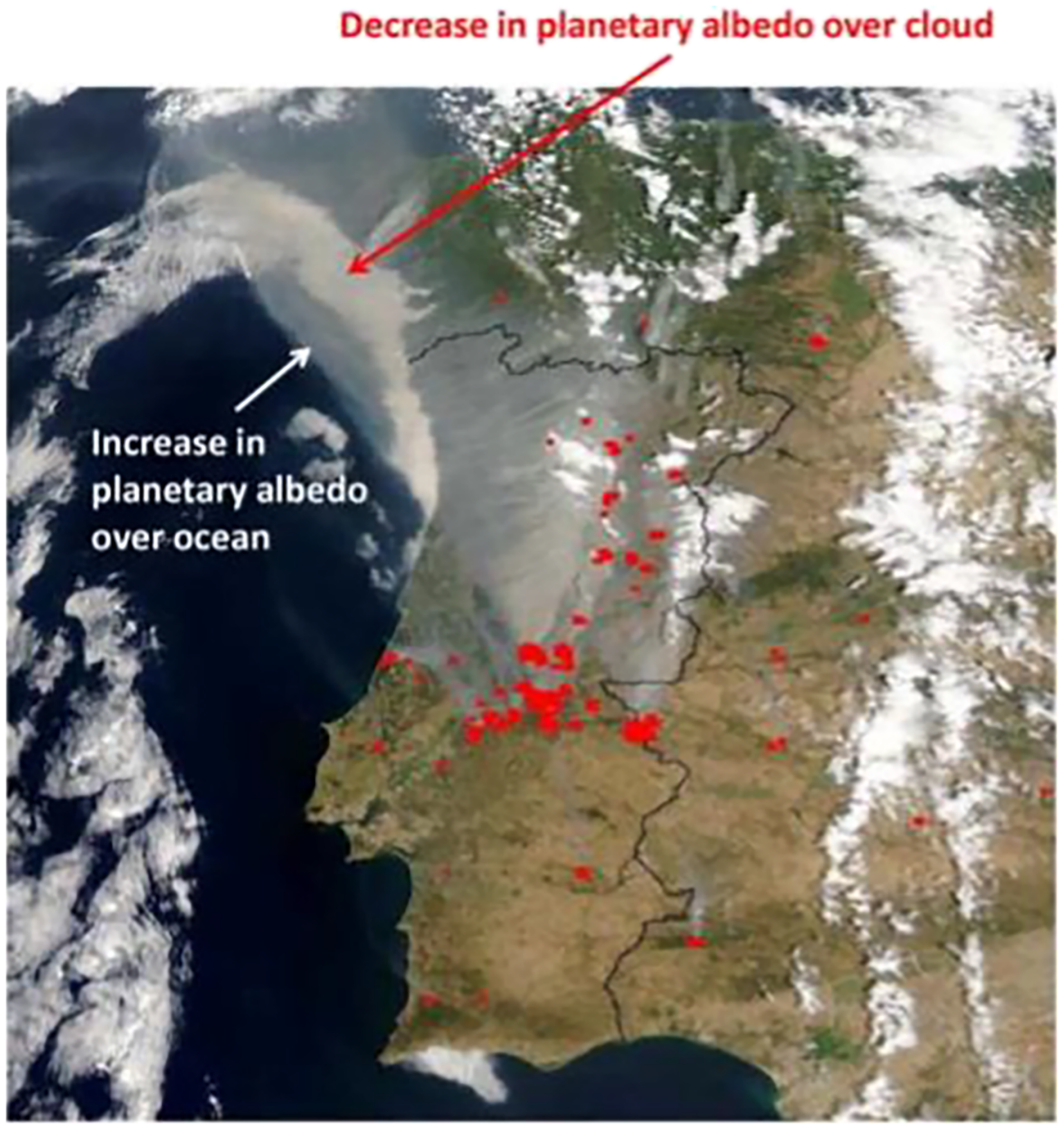
True‐color satellite image taken by the Moderate Resolution Imaging Spectroradiometer (MODIS) showing a plume of smoke from forest fires in Portugal on 3 August 2003. Fires are shown by the red spots; the smoke plume appears in gray. From Haywood (2015).

Locally and seen from the top of the atmosphere, aerosol particles can both increase or decrease the amount of radiation reflected to space, depending on the contrast between the brightness of the aerosols and that of the underlying surface. Bright (scattering) aerosols increase the local albedo when over dark surfaces but have less of an impact when over brighter surfaces. Conversely, dark (absorbing) aerosols decrease the albedo over bright surfaces but have less of an impact over darker surfaces. This effect is clearly demonstrated by the satellite image shown in Figure 3 showing biomass burning aerosol over the Iberian Peninsula. The absorbing smoke plume brightens the image when located over dark land and ocean surfaces but darkens it when overlying the bright cloud to the northwest.

Mathematically, that change in sign of the aerosol‐radiation interactions means that there exists a SSA, named critical SSA (Chýlek & Coakley, 1974) and denoted $\varpi_{0}^{\text{crit}}$, where aerosols have the same brightness as the underlying surface and thus exert no radiative perturbation in spite of interacting with radiation. Haywood and Shine (1995) have expressed $\varpi_{0}^{\text{crit}}$ as a function of the surface albedo, *α*_*s*_, and the mean fraction of radiation up‐scattered to space by the aerosols, *β*, as follows:

(3) $$\varpi_{0}^{\text{crit}}=\frac{2\;\alpha_{s}}{\beta {\left(1-\;\alpha_{s}\right)}^{2}+2\;\alpha_{s}}$$

Quantities in this equation are integrated and weighted over the solar spectrum. In practice, the critical SSA ranges from 0.7 and 0.8 over land surfaces (e.g., Gonzi et al., 2007) and is up to 0.9 over clouds (Costantino, 2012). Most aerosols from natural and human sources have a SSA larger than 0.9 and therefore typically increase reflection of radiation to space, but aerosols from agricultural and forest fires are often more strongly absorbing, and decrease reflection of radiation when located above clouds (e.g., Leahy et al., 2007; Zuidema et al., 2016). The point where the radiative effect of aerosol‐radiation interactions switches sign from negative to positive has alternatively been characterized as a critical surface albedo (King et al., 1999) or a critical cloud fraction (Chand et al., 2009).

Clouds affect aerosol populations. They act as a source of aerosol mass, because heterogeneous chemistry converts precursor gases into low‐volatile or nonvolatile chemical components of aerosol, and as a sink of aerosols because precipitation is the main pathway for removing aerosols from the atmosphere. But aerosols also affect clouds.

Aerosol‐cloud interactions are based, for liquid clouds, on the role aerosol particles play as cloud condensation nuclei (CCN), first identified by Aitken (1880) and then described thermodynamically by Köhler (1936). An anthropogenically driven increase in CCN concentrations therefore leads to more cloud droplets. Conover (1966), Hobbs et al. (1970) and Twomey (1974) presented observational evidence for increases in CCN resulting in increases in droplet number. More numerous droplets present an increased scattering cross section leading to an increase of the albedo of the cloud when liquid water path (LWP) is held constant. In radiative transfer the particle size is often measured by a droplet effective radius, *r*_e_, rather than the droplet number concentration, *N*_d_, so it is often stated that an increase in *N*_d_, for a given cloud liquid water content, implies a decrease in *r*_e_. This was the original formulation by Twomey (1977). Ship tracks, the quasi‐linear features of enhanced cloud albedo along the track of ships (Conover, 1966), are commonly cited as evidence for that cloud brightening.

Ice clouds also contribute to aci. This is the case when ice crystals form via homogeneous freezing of water droplets or aqueous aerosol particles, and because some aerosols serve as ice nucleating particles (INPs) (DeMott et al., 1997). Changes to liquid droplets may also have later implications for the ice phase in mixed‐phase clouds (Coopman et al., 2018; Norgren et al., 2018). Observations of higher concentrations of smaller ice crystals in cirrus clouds polluted by aircraft exhaust were first made by Ström and Ohlsson (1998) and Zhao et al. (2018) found similar correlations in satellite retrievals, although seasonal variations in water vapor overwhelm the aerosol signature. Vergara‐Temprado, Miltenberger, et al. (2018) have shown by comparing a global model to satellite retrievals of radiative fluxes that INP concentrations can strongly alter the reflectivity of shallow mixed‐phase clouds. However, evidence for a Twomey effect acting on ice clouds is far from being as strong as for liquid clouds.

The list of rapid adjustments associated with aci is long. Because of different processes, adjustments in liquid clouds, in mixed‐phase, and ice clouds are usually considered separately. But even among clouds of the same phase, differences in cloud dynamics or environmental conditions may influence the sign of the adjustment. Adjustments in liquid clouds have been hypothesized through aerosol increases driving delays in precipitation rates (Albrecht, 1989) and increases in cloud thickness (Pincus & Baker, 1994) that would manifest themselves as increases in cloud LWP or changes in cloud fraction (CF). Altered droplet size distributions also affect entrainment mixing of clouds with environmental air, possibly reducing LWP (Ackerman et al., 2004; Small et al., 2009). The latter adjustments might reduce the increase in cloud albedo (Stevens & Feingold, 2009). Adjustments in mixed phase and ice clouds stem from different mechanisms. Responses of these clouds to aerosols include more frequent glaciation of supercooled water because of preferential freezing onto increased INP (Lohmann, 2002), deactivation of INP because of changes in aerosol mixing state (Girard et al., 2004; Hoose et al., 2008; Storelvmo et al., 2008), changes in precipitation and consequently cloud water path and cloud reflectivity (Vergara‐Temprado, Miltenberger, et al., 2018), invigoration of convection from suppression of precipitation and latent heat release (Khain et al., 2001; Koren et al., 2005), and increase in lightning occurrence in deep convective clouds (Thornton et al., 2017).

Aerosols may also exert an RF after their removal from the atmosphere. Aerosol‐surface interactions refer to changes in albedo from the deposition of absorbing aerosols on to bright—for example, snow‐ and ice‐covered—surfaces. Initially hypothesized by Bloch (1965) to explain past changes in sea level, the impact of aerosols on snow albedo was quantified by Warren and Wiscombe (1980), who showed that including in‐snow aerosol absorption in a radiative transfer model better fits albedo measurements made in the Arctic and Antarctica. Rapid adjustments to aerosol‐surface interactions involve changes in snow grain size and the timing of melting of the snow pack (Flanner et al., 2007). Since such effects are relevant only in confined regions, they are not assessed in detail in this review.

Compared to greenhouse gases, aerosols exhibit much more variable chemical compositions and much shorter atmospheric residence times, but much greater forcing per unit mass from interaction with radiation. For ari, aerosol scattering and absorption cross sections depend on the wavelength of the radiation and the physical and chemical properties of the aerosol (see box). The sign and strength of the RF due to ari, RFari, is modulated further by environmental factors, including incident radiation, relative humidity, and the albedo of the underlying ocean, land surface or cloud (See Figure 3). For aci, the ability of aerosol particles to serve as CCN or INPs depends on the number concentration, size distribution, solubility, shape, and surface chemical properties of the particles. In addition, cloud type or cloud regime, that is, discrimination between cumuliform and stratiform clouds, as well as clouds in different altitudes (WMO, 2017) is a strong determinant of the complex responses of cloud processes to an aerosol‐driven increase in drop number, and those cloud processes may be more important and uncertain for aci than aerosol processes (Gettelman, 2015). Even if all of these issues could be addressed accurately, uncertainty would remain due to uncertainty in the reference state (Carslaw et al., 2013), increasingly so the further back in time one adopts a baseline.

### 2.2 Scope and Definitions

The scope of this review is globally averaged aerosol ERF because the concept of ERF is mostly relevant to the understanding of climate change in a global sense. Consequently, ERF from aerosol‐surface interactions due to deposition of absorbing aerosols on to snow and ice is not considered here because it comes primarily from local areas within high latitude regions or high mountain ranges and does not contribute much to the globally averaged ERF (Jiao et al., 2014). The strong regional variations in aerosol distributions and ERF may matter for determining impacts of aerosol ERF on several aspects of the Earth system (e.g. Bollasina et al., 2011; Chung and Soden, 2017; Kasoar et al., 2018), but those considerations are also not addressed in this review. Both RF and ERF are measured in W m^−2^ and cover both the solar (shortwave, SW) and terrestrial (longwave, LW) parts of the electromagnetic spectrum.

Although this review adopts the IPCC definitions of RF and ERF (Myhre, Shindell, et al., 2013), it differs from previous IPCC practices in two ways. First, the reference year is chosen to be 1850 instead of 1750. Although 1750 represents a preindustrial state when fossil fuel combustion emissions were negligible, there is no evidence for 1750 being special from an aerosol point of view, as agricultural fires occurred well before that. In addition, 1850 matches the start of most surface temperature records and also the start of the historical climate simulations of the Coupled Model Intercomparison Project (CMIP; Eyring et al., 2016). This match is important because having coincidence in the starting year is beneficial to comparing the change in forcing with the change in temperature. The difference in RF between the two reference years is smaller than 0.1 W m^−2^ (Myhre, Shindell, et al., 2013; Carslaw et al., 2017) because industrialization was still in its early stages in 1850. The ERF between the years 1750 and 1850 has been estimated at −0.2 W m^−2^ by the IPCC AR5 (Myhre, Shindell, et al., 2013) but it could have been as weak as −0.028 W m^−2^ according to simulations using more recent emissions (Lund et al. 2019). For present day, (Myhre, Shindell, et al., 2013) used 2011 but this review is slightly more generic so present day refers here to average aerosol concentrations over the period 2005–2015. Second, this review will not attempt to bound aerosol RF mechanisms for which lines of evidence remain fragile, which increases the possibility that the bounds derived here are too conservative. Consequently, uncertainty ranges are given in this review as 16–84% confidence intervals (68% likelihood of being in the ranges given, equivalent to ±1‐*σ* for a normal distribution) instead of the 5–95% confidence interval (90% likelihood of being in the range) generally considered in IPCC Assessment Reports. The main uncertainty ranges are however translated to 5–95% confidence intervals in section 11 and Table 5 to make comparison easier.

To quantify the confidence intervals for RF and ERF, this review will need to combine the 16–84% confidence intervals obtained for different quantities. To do so, each 16–84% confidence interval is first expanded to a full interval (0–100% confidence) by assuming that probabilities are uniformly distributed within the interval, that is, by extending the range by a factor 100/68. Full intervals are then sampled randomly 10 million times in a Monte Carlo framework similar to that of Boucher and Haywood (2001), with the difference that they applied the uniform distribution approach to a case where the terms are added rather than multiplied. Finally, the resulting intervals are reported with 16–84% confidence.

## 3 Conceptual Model and Lines and Evidence

### 3.1 Conceptual Model

The net radiative flux, *R*, at the top of the atmosphere is the difference between the globally and annually averaged absorbed insolation (SW), $R_{\text{SW}}^{↓}\;(1-\alpha )$, and outgoing terrestrial (LW) irradiance, $R_{\text{LW}}^{↑}$:

(4) $$R=R_{\text{SW}}^{↓}(1-\alpha )-R_{\;\text{LW}}^{↑}\approx 0$$

where the near equality of the two denotes a state of stationarity. The albedo, *α*, the fraction of the insolation that is scattered back to space, depends on the properties of the atmosphere, the surface, and the angle of illumination. Aerosol perturbations primarily affect *α*, in which context their effect stems from changes in the column‐integrated extinction coefficient of the aerosol, called aerosol optical depth (AOD) and denoted *τ*_a_, and in cloud droplet number concentrations, *N*_d_. *τ*_a_ is usually dominated by scattering, but some subcomponents of the aerosol are also absorbing in the SW or LW parts of the electromagnetic spectrum and contribute to the net irradiance absorbed by the atmosphere, *R*_atm_. Similarly to extinction, aerosol absorption is usually quantified by the aerosol absorption optical depth, *τ*_abs_. *N*_d_ depends on another subcomponent of the aerosol, namely, the number of hygroscopic aerosol particles that serve as CCN. Anthropogenic aerosol, through its forcing and consequent rapid adjustments of clouds as well as through its direct interaction with terrestrial radiation, may also contribute to changes in $R_{\text{LW}}^{↑}$. Aerosol‐induced changes in ice clouds may also influence *R*. Changes in surface properties are assumed small relative to the magnitude of the other components in the global annual mean.

Adopting this description leads to the expectation that a change in the amount or properties of aerosol influences the net irradiance and thus exerts an RF, F, as follows

(5) $$F=\Delta R=\Delta \tau_{a}\;\frac{\partial R}{\partial \tau_{a}}+\Delta \ln N_{d}\;{\left.\frac{\partial R}{\partial \ln N_{d}}\right|}_{L,C}$$

where Δ*τ*_a_ and $\Delta \ln N_{d}$ denote the perturbation in global AOD and relative perturbation in cloud droplet number concentration, respectively, taken here as the difference between 1850 and an average year between 2005 and 2015, hereafter called for convenience “preindustrial” and “present‐day,” respectively. L denotes the cloud LWP and C the cloud fraction, and the second partial derivative therefore excludes changes in those quantities, following Twomey (1974). Equation (5) is valid for a given point in space and time. Perturbations in *τ*_a_ and *N*_d_ are not independent, but the two terms in equation (5) assume a decoupling between radiative changes originating in the clear part of the atmosphere from those originating in the cloudy part of the atmosphere. However, it should be noted that this assumption is not equivalent to decoupling changes in clear‐sky and cloudy‐sky radiative fluxes.

Rapid adjustments are added to F to obtain the ERF, E. For ari, this consists of a term describing changes to *R*_atm_ driven by changes in *τ*_a_. Changes in *R*_atm_ then impact *R*, including $R_{\text{LW}}^{↑}$, and cloud amount. For aci, this modifies the sensitivity of *R* to changes in *N*_d_ to allow for changes in L and C, in cloud top temperature and hence $R_{\text{LW}}^{↑}$, and in ice clouds. The inclusion of rapid adjustments is represented mathematically by moving from partial to total derivatives:

(6) $$E=\Delta \tau_{a}\;\frac{\partial R}{\partial \tau_{a}}+\Delta \tau_{a}\;\frac{dR}{dR_{\text{atm}}}\;\frac{dR_{\text{atm}}}{d\tau_{a}}+\Delta \ln N_{d}\;\frac{dR}{d\ln N_{d}}.$$

The literature does not decompose rapid adjustments of *τ*_a_ or *τ*_abs_ on cloud properties into adjustments of C and L separately (Bond et al., 2013; Koch & Del Genio, 2010), so these rapid adjustments are included in the overall sensitivity of *R*_atm_ to *τ*_a_ through the second term on the right‐hand side of equation (6). In contrast, such decomposition is commonly performed for aci (Chen et al., 2014; Gryspeerdt, Goren, et al., 2019; Mülmenstädt et al., 2019; Quaas et al., 2008; Sekiguchi et al., 2003). For a given point in space and time, the sensitivity of *R* to changes in *N*_d_, $\frac{dR}{d\ln N_{d}}$, neglecting the changes in ice clouds and in cloud top temperature, consists of the change in response solely due to changes in *N*_d_ with everything else constant—relevant for the RF due to aci, RFaci (equation (5))—and the radiative impact of the adjustments. The sensitivity is best expressed as logarithmic in *N*_d_, because most cloud processes are sensitive to a relative, rather than absolute, change in *N*_d_ (Carslaw et al., 2013, see also Eq. 17). This approach is also supported by satellite data analyses (e.g., Kaufman & Koren, 2006; Nakajima et al., 2001; Sekiguchi et al., 2003). The total response of *R* to relative perturbations in *N*_d_ can therefore be expanded as

(7) $$\frac{dR}{d\ln N_{d}}\approx {\left.\frac{\partial R}{\partial \ln N_{d}}\right|}_{L,C}+\frac{\partial R}{\partial C}\frac{dC}{d\ln N_{d}}+\frac{\partial R}{\partial L}\frac{dL}{d\ln N_{d}}=S_{N}+S_{C,N}+S_{L,N}.$$

The first and third terms are restricted to cloudy regions. The last step defines the denotation of the three terms as radiative sensitivities, *S*_*N*_, $S_{C,N}$, and $S_{L,N}$.

In some cases, usually under idealized conditions, the sensitivities expressed by the partial derivatives in equations (6) and (7) can be calculated theoretically or inferred observationally. For instance, under clear skies *∂R*/*∂τ*_a_ can be calculated locally and averaged over different scenes to get a global sensitivity of top‐of‐atmosphere net radiation to changes in *τ*_a_. To relate this global sensitivity to the global, all‐sky response requires also accounting for situations where there is little sensitivity. For instance, over a sufficiently bright background, like a snow‐covered surface or a cloud, increasing the clear‐sky scattering will have no appreciable effect on *α*, irrespective of the magnitude of the aerosol perturbation. Likewise, over a dark surface increasing aerosol absorption has little effect on *α* (see box).

This assessment targets the global, annual mean aerosol ERF so there is a need to integrate equations (6) and (7), which are valid at a given location in space and time, globally and over periods of time long enough to eliminate variability from changes in the weather. In particular, the aci sensitivities defined in equation 7 require averaging globally over the different cloud regimes that experience changes in *N*_d_. Weighting factors are introduced to account for those spatial and temporal dependencies, following Steven (2015). Although these weighting factors are related to cloud amount, clouds span a distribution of optical depths and their optical depth differently mediates the extent to which they mask ari or express aci. So the weighting factors are effective cloud fractions, denoted *c*, and the effective clear‐sky fraction need not be the complement of the effective cloudy‐sky fraction. The introduction of the weighting factors allows for an attractive framework to quantify the aerosol RFs and their uncertainties, at the expense of having to quantify the uncertainties of the weighting factors themselves. These uncertainties may be larger than the uncertainty on CF but arguments can be made to estimate them.

Effective cloud fractions *c*_*τ*_, *c*_*N*_, $c_{C}$, and $c_{L}$ are therefore introduced for each term in equation 8. They are formally defined, and quantified from the literature, in sections 5, 6, and 8, respectively. Consequently, the individual terms in equations (6) and (7) are parameterized as a product of the change in the global aerosol or cloud state, idealized sensitivities (*S*) and those weighting factors (*c*). Applying this approach to equations (6) and (7) yields the following formula for:

(8) $$E=\Delta \tau_{a}\;\left[S_{\tau }^{\text{clear}}\;(1-c_{\tau })+S_{\tau }^{\text{cloudy}}\;c_{\tau }+\frac{dR}{dR_{\text{atm}}}\;\frac{dR_{\text{atm}}}{d\tau_{a}}\right]+\Delta \ln N_{d}\left[S_{N}\;c_{N}+S_{C,N}\;c_{C}+S_{L,N}\;c_{L}\right]$$

The term representing Fari has been decomposed into cloud‐free and cloudy contributions to properly account for the masking or enhancement of ari by clouds, as discussed above. The sensitivity $S_{\tau }^{\text{clear}}$ is defined as $\frac{\partial R_{\text{clear}}}{\partial \tau_{a}}$. Similarly, $S_{\tau }^{\text{cloudy}}$ is defined as $\frac{\partial R_{\text{cloudy}}}{\partial \tau_{a}}$. Sensitivities that are a product of two partial derivatives, as defined by equation (7), are denoted by a double subscript. For reference, Table 2 summarizes the definitions of the variables used in equation (8).

**Table 2:** Mathematical Definitions and Descriptions of the Variables of Equations (8), (15), and (24)

Section	Mathematical definition	Description
4	$\tau_{a}^{\text{PD}}$	Present day (2005–2015) *τ*_a_
4	$\Delta \tau_{a}=\tau_{a}^{\text{PD}}-\tau_{a}^{\text{PI}}$	Change in *τ*_a_ between present day (2005–2015)
		and preindustrial (1850)
4	$\Delta \ln\tau_{a}=\Delta \tau_{a}/\tau_{a}^{\text{PD}}$	Relative change in *τ*_a_ over the industrial era
6	$\Delta \ln N_{d}=\Delta N_{d}/N_{d}$	Relative change in *N*_d_ over the industrial era
Aerosol‐radiation interactions		
5	$S_{\tau }^{\text{clear}}=\partial R_{\text{clear}}/\partial \tau_{a}$	Sensitivity of *R* to changes in *τ*_a_ in clear (cloud‐free) sky
5	$S_{\tau }^{\text{cloudy}}=\partial R_{\text{cloudy}}/\partial \tau_{a}$	Sensitivity of *R* to changes in *τ*_a_ in cloudy‐sky
5	$c_{\tau }=\frac{\left\langle \tau_{c}\;\Delta \tau_{a}\;C\right\rangle }{\left\langle \tau_{c}\;\Delta \tau_{a}\right\rangle }$	Effective cloud fraction for RFari
7	*dR*/*dR*_atm_	Sensitivity of *R* to changes in
		atmospheric absorption
7	*dR*_atm_/*dτ*_a_	Sensitivity of atmospheric absorption to
		changes in *τ*_a_
Aerosol‐cloud interactions		
6	$\beta_{\ln N-\ln\tau }=\frac{\partial \ln N_{d}}{\partial \ln\tau_{a}}$	Sensitivity of *N*_d_ to changes in *τ*_a_
6	$S_{N}={\left.\frac{\partial R}{\partial \ln N_{d}}\right|}_{L,C}$	Sensitivity of *R* to changes in *N*_d_ at constant L and C
6	$c_{N}=\frac{\left\langle C_{\text{liq}}\;\alpha_{c}\left(1-\alpha_{c}\right)\;\beta_{\ln N-\ln\tau_{a}}\;\frac{\Delta \tau_{a}}{\tau_{a}}\;R_{\text{SW}}^{↓}\right\rangle }{\left\langle \alpha_{c}\;\left(1-\alpha_{c}\right)\rangle \langle \beta_{\ln N-\ln\tau_{a}}\rangle \langle \frac{\Delta \tau_{a}}{\tau_{a}}\rangle \langle R_{\text{SW}}^{↓}\right\rangle }$	Effective cloud fraction for RFaci
8	$\beta_{\ln L-\ln N}=\frac{\partial \ln L}{\partial \ln N_{d}}$	Sensitivity of L to changes in *N*_d_
8	$S_{L,N}=\frac{\partial R}{\partial L}\frac{dL}{d\ln N_{d}}$	Sensitivity of *R* to changes in L
		mediated by changes in *N*_d_
8	$c_{L}=\frac{\left\langle C_{\text{liq}}\;\alpha_{c}\;\left(1-\alpha_{c}\right)\beta_{\ln L-\ln N_{d}}\;\beta_{\ln N_{d}-\ln\tau_{a}}\;\frac{\Delta \tau_{a}}{\tau_{a}}R_{\text{SW}}^{↓}\right\rangle }{\left\langle \alpha_{c}\;\left(1-\alpha_{c}\right)\rangle \;\langle \beta_{\ln L-\ln N_{d}}\rangle \;\langle \beta_{\ln N_{d}-\ln\tau_{a}}\rangle \;\langle \frac{\Delta \tau_{a}}{\tau_{a}}\rangle \langle R_{\text{SW}}^{↓}\right\rangle }$	Effective cloud fraction for rapid adjustments in L
8	$\beta_{C-\ln N}=\frac{\partial C}{\partial \ln N_{d}}$	Sensitivity of C to changes in *N*_d_
8	$S_{C,N}=\frac{\partial R}{\partial C}\frac{dC}{d\ln N_{d}}$	Sensitivity of *R* to changes in C
		mediated by changed in *N*_d_
8	$c_{C}=\frac{\left\langle \left(1-C_{\text{ice}}\right)\;\left(\alpha_{c}-\alpha_{\text{clear}}\right)\;\beta_{C-\ln N}\;\beta_{\ln N-\ln\tau_{a}}\;\frac{\Delta \tau_{a}}{\tau_{a}}\;R_{\text{SW}}^{↓}\right\rangle }{\left\langle \left(\alpha_{c}-\alpha_{\text{clear}}\right)\rangle \langle \beta_{C-\ln N}\rangle \;\langle \beta_{\ln N-\ln\tau_{a}}\rangle \;\langle \frac{\Delta \tau_{a}}{\tau_{a}}\rangle \langle R_{\text{SW}}^{↓}\right\rangle }$	Effective cloud fraction for rapid adjustments
		in cloud fraction

*Note*. The first column gives the number of the section where the uncertainty range for each variable is assessed. *τ*_a_ and *τ*_c_ are the aerosol and cloud optical depths, respectively. *N*_d_ is the cloud droplet number concentration. L is the liquid cloud water path. C is the cloud fraction, and $C_{\text{liq}}$ and $C_{\text{ice}}$ are the liquid and ice cloud fractions, respectively. *R* is the sum of shortwave and longwave radiation at the top of the atmosphere, *R*_atm_ is the radiation absorbed in the atmosphere, and $R_{\text{SW}}^{↓}$ is the downwelling shortwave radiation at cloud top. *α*_c_ and *α*_clear_ are the cloud and cloud‐free albedos, respectively. Angle brackets denote global‐area‐weighted temporal averaging.

An important and long standing objection to the approach embodied by equation (8) is that because aerosol perturbations are large and local, their effects are nonlinear, and cannot be related to perturbations of the global aerosol state. However, such effects can be incorporated into the weighting factors. For instance, when applying the interpretive framework of equation (8) to the output from models that spatially and temporally resolve ari, it becomes possible to assess the extent to which differences arise from differences in how they represent the intrinsic sensitivity, *S*_*τ*_, the magnitude of the perturbation, Δ*τ*_a_, or the way in which local effects are scaled up globally, as measured by *c*_*τ*_. To the extent that nonlinearities are important—and often for global averages of very nonlinear local processes they are not—it means that the weighting factors, *c*, may be situation dependent, and their interpretation may be nontrivial.

The ari term of equation (8) has been assumed linear in Δ*τ*_a_. This assumption is justified by a series of arguments that starts at the source of the aerosol. For primary aerosols, aerosol number concentrations are linear in the emission rate. For secondary aerosols, linear relationships between emissions of gaseous precursor and RFari have been found at the global scale, including for precursors like dimethyl‐sulfide (Rap et al., 2013). The aerosol population undergoes fast microphysical aging processes right after emission or nucleation (Jacobson & Seinfeld, 2004), changing its size, composition, and mixing state. These microphysical processes grow anthropogenic nanoparticles into sizes comparable to the wavelength of the radiation, where aerosols interact efficiently with radiation. Preexisting aerosol particles act both as condensational sinks of gas‐phase precursors and as seeds to efficiently grow semivolatile aerosol precursors to ari‐relevant sizes. The overall scaling of secondary aerosol number concentrations from nucleation therefore depends on relative emission rates of primary and secondary aerosol precursors. Estimates from global microphysical aerosol models that include aerosol nucleation, condensation and coagulation confirm nonlinear responses to the coemission of primary carbonaceous aerosols and sulfur dioxide (SO_2_, a precursor to sulfate aerosols), in particular near aerosol source regions (Stier et al., 2006). However, these deviations do not exceed 30% locally for accumulation mode number concentrations and 15% for *τ*_a_, sufficiently small to be assumed linear in the global mean context of this review. Further, ari scales fairly linearly with Δ*τ*_a_ for a given SSA (Boucher et al., 1998). The SSA of the aerosol population, which moderates the top‐of‐atmosphere ERF (see box), depends on the composition of aerosol sources, specifically the fraction of anthropogenic absorbing aerosols and notably black carbon (BC; also called soot (Bond et al., 2013)) aerosols. These factors affect the clear‐sky albedo sensitivity *S*_*τ*_ and the atmospheric absorption efficiency d*R*_atm_/d*τ*_a_.

Finally, equation (8) may require an additional term to represent changes in ice cloud properties in response to changes in ice crystal number, but scientific understanding is not there yet to support a quantitative assessment of that term, as discussed in section 9.

### 3.2 Lines of Evidence

From a historical point of view, process‐oriented studies at the relevant aerosol and cloud scales are the foundation of the conceptual thinking of aerosol RF. Observational and modeling tools have led to investigations that have helped refine process understanding and generate further lines of investigation with increasingly sharp tools.

For the purpose of this review, lines of evidence are grouped into three categories: estimation of sensitivities of radiation and clouds to aerosol changes; estimation of large‐scale changes in the aerosol and cloud states over the industrial era; and inferences from observed changes in the overall Earth system.

#### 3.2.1 Estimation of Sensitivities

There are several methods with the potential to estimate sensitivities of radiation and clouds to aerosol changes:

- In situ observations using ground‐based and airborne instruments;
- Remote sensing observations from ground‐based networks, airborne, and satellite platforms;
- Process‐based modeling at small scales using cloud‐resolving models or large eddy models.

Airborne measurements that combine cloud droplet size, droplet number, liquid water and cloud‐reflected radiance (Brenguier et al., 2003; Werner et al., 2014), and high‐quality ground‐based measurements, for example, from supersites, have provided strong, quantitative evidence for aerosol effects on cloud microphysics (Brenguier et al., 2003; Feingold et al., 2003; Garrett et al., 2003; Kim et al., 2003; Twomey & Warner, 1967). However, translating those effects to the radiative response and deriving sensitivities remains a challenge. For example, negative correlations between droplet size and aerosol concentration support the underlying theory proposed by Twomey (1977) but confound droplet size responses to aerosol with cloud water responses to the aerosol and its associated meteorology (e.g. Brenguier et al., 2003) and thus make it difficult to unravel the net radiative response. Quantification of sensitivities has proven to be contingent on a variety of factors, including choice of instrument, retrieval accuracy (Sena et al., 2016), aggregation scale (McComiskey & Feingold, 2012), and cloud regime. Drizzle is also a confounding factor that obscures the relationships, reducing droplet number and increasing droplet size, as well as removing the aerosol (e.g., Feingold et al., 1996; Wood et al., 2012). In addition, in situ observations have thus far covered only a limited number of locations on the globe for varying duration and have sampled only a limited number of cloud regimes. The extent to which present understanding and estimates of Faci would be changed by future measurements is not known.

Satellite instruments provide the coverage in space and time necessary to evaluate sensitivities on the global scale. Aerosols and clouds are usually not retrieved in the same pixels, and there is some fuzziness in the distinction between thick haze and thin clouds. Satellite data are best used in conjunction with process understanding to factor out covariabilities for which a causal influence by the aerosols may be difficult to ascertain. For example, aerosol and *N*_d_ may be simultaneously low simply because of precipitation, leading to aerosol removal, rather than because of aci affecting droplets. Relationships have been found between *τ*_a_ in cloud‐free air and a variety of properties of nearby clouds: cloud droplet size (Nakajima et al., 2001; Sekiguchi et al., 2003), cloud fraction (Kaufman et al., 2005), cloud top pressure (Koren et al., 2005), shortwave radiative fluxes (Loeb & Schuster, 2008; Oreopoulos et al., 2016), precipitation (Lebsock et al., 2008) and lightning (Yuan, Remer, Pickering, & Yu, 2011). However, translating those relationships to physically meaningful sensitivities is difficult because variations in meteorological factors, such as humidity or atmospheric stability, affect both aerosol and cloud properties, generating correlations between them which are not necessarily causal in nature (Boucher & Quaas, 2013; Mauger & Norris, 2007). Constructs such as the albedo susceptibility (Platnick & Twomey, 1994) or precipitation susceptibility (Sorooshian et al., 2009) are useful in that they survey globally the regions of the Earth that have the potential to generate large responses to aerosol perturbations while controlling for key meteorologically driven variables. Progress in accounting for spurious correlations (i.e., correlations that do not imply a causal aerosol effect on the respective cloud property) has been made using statistical techniques (Gryspeerdt et al., 2016), careful sampling (Christensen et al., 2017) and through combination with reanalysis data (Koren, Feingold, & Remer, 2010; McCoy, Bender et al., 2018).

In addition to cloud albedo and cloud amount responses, fine‐scale models have highlighted other more nuanced, and potentially important aci processes like evaporative‐entrainment feedbacks (Ackerman et al., 2004; Hill et al., 2009; S. Wang et al., 2003; Xue & Feingold, 2006), sedimentation‐entrainment feedbacks (Bretherton et al., 2007), and boundary layer decoupling (Sandu et al., 2008). The consequences for the ERF are complex. In some conditions, the aerosol‐cloud system is resilient to perturbation (“buffered”) as a result of adjustments to the amount of cloud water (Stevens & Feingold, 2009) and sensitivities are small. In contrast, aerosol‐mediated transitions between closed cellular convection and open cellular convection (Goren & Rosenfeld, 2012) are associated with large sensitivities, but as those transitions are likely contingent on meteorological state (Feingold et al., 2015), their global significance is not yet known.

#### 3.2.2 Estimation of Large‐Scale Changes

Because unperturbed preindustrial aerosol and cloud distributions have not been observed, evaluation of Δ*τ*_a_ and $\Delta \ln N_{d}$ requires large‐scale modeling based on physical parameterizations of key processes. Large‐scale models, which are designed around the idea of integrating the essential processes of ari and aci at the global scale, could in principle be a useful tool to quantify aerosol ERF. This is in part because they are intended to physically account for energy exchanges through the Earth system and suited to analyzing the energy budget of the Earth and also because they are built to translate hypotheses on preindustrial emissions into estimates of preindustrial aerosol and cloud distributions. But especially for aci, the more nuanced cloud responses to drop number perturbations described above are driven by processes that act at scales much smaller than General Circulation Model (GCM) resolutions. Consequently, they can be represented in GCMs only by empirical, and thus inherently uncertain, parameterizations, and so their global applicability and importance are uncertain. GCMs therefore carry the uncertainties in forcing associated with less than ideal representation of aerosol and cloud processes. An important risk is therefore overinterpretation of model sensitivities to well‐studied processes while neglecting other important processes that are poorly represented because they act at scales smaller than those resolved by large‐scale models (Mülmenstädt & Feingold, 2018).

Nonetheless, when used correctly, large‐scale models help constrain significant parts of the ari and aci problem and are powerful tools for hypothesis testing about the impact of particular processes. Because of the uncertainties discussed above, global climate models (GCMs) produce a range of possible RFari (Myhre, Samset, et al., 2013) and ERFaci (Ghan et al., 2016). Understanding the causes of differences among global models has been one of the main objectives of the Aerosol Comparisons between Observations and Models (AeroCom) initiative since its inception in 2003 (Kinne et al., 2006; Schulz et al., 2006; Textor et al., 2006). Diversity among models can result from structural differences that arise from the use of different radiative transfer parameterizations, aerosol and cloud schemes, and surface albedo (Boucher et al., 1998; Fiedler et al., 2019; Ghan et al., 2016; Halthore et al., 2005; Penner et al., 2009; Randles et al., 2013; Stier et al., 2013). Diversity can result also from parametric differences, which arise from the imperfect knowledge of the parameters used in physical parameterizations as well as in boundary conditions like aerosol emissions. Parametric uncertainty can be quantified using perturbed parameter ensembles (PPEs) (Carslaw et al., 2013; Lee et al., 2011; Regayre et al., 2018). PPEs involve randomly perturbing model parameters within expert‐elicited ranges to generate an ensemble that unfolds most of the uncertainty associated with the tuning process of the original model. A PPE applied on the Hadley Centre climate model by considering uncertainties in both the aerosol representation and the host physical climate model found a 95% confidence interval for the parametric uncertainty, constrained by top‐of‐atmosphere radiative budget observations, of −2.3 to −0.6 W m^−2^ for aerosol ERF. This interval is shifted to more negative values compared to the expert judgment, guided by various observational and modeling considerations, of Boucher et al. (2013).

#### 3.2.3 Integral Energy Balance Inferences

This distinct line of evidence, also called top‐down approaches, builds inferences on RF and climate feedback and response (equation (1)) based on the time evolution of, for example, surface temperature and surface radiative fluxes. An example of such a top‐down inference is asserting that temperature changes and net forcing must have a common sign, so aerosol ERF must be less negative than total nonaerosol ERF. Those inferences are often interpreted with energy balance models. This particular line of evidence is discussed in section 10.

## 4 Preindustrial to Present‐Day Change in AOD

While present‐day aerosol properties such as the AOD, $\tau_{a}^{\text{PD}}$, considered here at a wavelength of 0.55 μm, can be measured directly by ground‐based Sun photometers at certain locations or retrieved from satellite observations, the preindustrial (defined here as 1850) value, $\tau_{a}^{\text{PI}}$, is not observable so $\Delta \tau_{a}=\tau_{a}^{\text{PD}}-\tau_{a}^{\text{PI}}$ can only be estimated.

Direct measurements of $\tau_{a}^{\text{PD}}$ come from the ground‐based AErosol RObotic NETwork (AERONET) Sun photometer network (Holben et al., 1998; Smirnov et al., 2009), which provides near‐global but sparsely sampled, cloud‐free hourly $\tau_{a}^{\text{PD}}$ at accuracies better than 0.01 (Eck et al., 1999; Smirnov et al., 2000). With added information of their sky radiances samples, the AERONET Sun photometers also provide information on vertically integrated aerosol size and light absorption. However, continental and often near‐urban locations lead to systematic biases (R. Wang et al., 2018). To supplement these measurements, long‐term satellite remote sensing retrievals are also available, with more than 30 years of passive measurements, including almost two decades of Moderate Resolution Imaging Spectroradiometer (MODIS) aerosol retrievals; and more than a decade of active measurements using the Cloud‐Aerosol Lidar with Orthogonal Polarization (CALIOP). Passive satellites retrieve an AOD from the measured radiance after carefully screening for clouds. Those AOD retrievals are based on radiative transfer calculations that take into account illumination and viewing geometry, extinction by Rayleigh scattering (with attendant assumption on aerosol height), surface reflectance, and aerosol properties (especially angular dependence of scattering and SSA). Levy et al. (2013) evaluate the uncertainty in global mean $\tau_{a}^{\text{PD}}$ from MODIS to about ±0.03, or 15% to 20%. Current satellite lidar retrieval of aerosol extinction requires the ratio of extinction to backscatter that depends on aerosol particle radius, sphericity, and SSA. An aerosol typing algorithm is used to choose from a set of default lidar ratio values, but this is a significant source of retrieval uncertainty. Globally averaged clear‐sky $\tau_{a}^{\text{PD}}$ at 0.55 μm from MODIS/Aqua Collection 6 is, at 0.17, about 30% larger than CALIOP Version 3 at about 0.12 (Winker et al., 2013). The true value is likely somewhere in between, because systematic errors in the MODIS retrieval, mostly driven by errors in surface albedo and cloud artifacts, tend to bias $\tau_{a}^{\text{PD}}$ high, whereas systematic CALIOP errors tend to bias $\tau_{a}^{\text{PD}}$ low (Kittaka et al., 2011). Among the retrieval algorithms applied to measurements by MODIS, the Advanced Along‐Track Scanning Radiometer (AATSR), and the Sea‐Viewing Wide Field‐of‐View Sensor (SeaWIFS), the lowest global mean $\tau_{\text{PD}}^{a}$ of 0.13 is obtained by the DeepBlue algorithm (Hsu et al. 2013) applied to SeaWIFS and the largest, at 0.17, is obtained by both the DarkTarget algorithm (Levy et al. 2013) applied to MODIS/Terra and the Oxford‐RAL Aerosol Cloud algorithm (Thomas et al. 2009) applied to AATSR.

**Figure 4:**
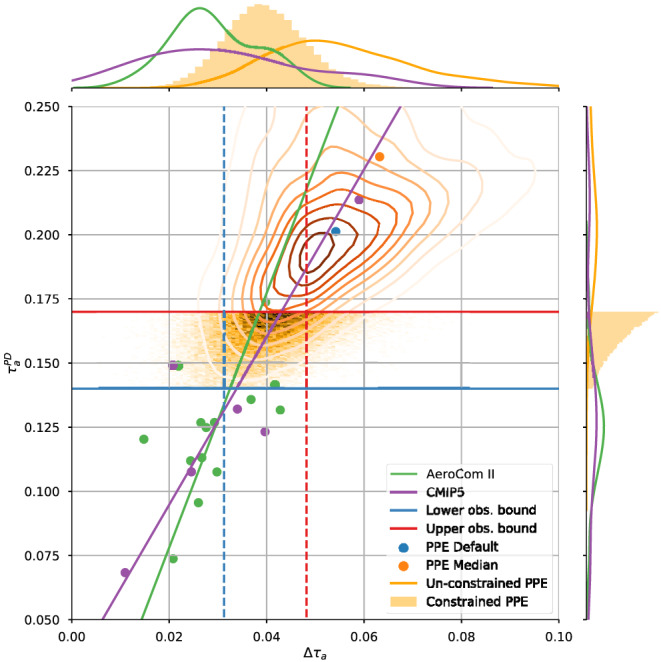
Distributions, standard deviation, and best fit lines of the present‐day aerosol optical depth, $\tau_{a}^{\text{PD}}$ against the industrial‐era change in aerosol optical depth at 0.55 μm, Δ*τ*_a_, between 1850 and present day, simulated for cloud‐free conditions by AeroCom Phase II and CMIP5 sstClimAerosol models. The full joint‐probability distribution sampled from the emulated HadGEM‐UKCA 26 aerosol parameter perturbed parameter ensemble (PPE) is shown as contour lines and the constrained distribution as a hex density. The default and median model runs of the PPE are also shown for completeness. The horizontal lines show the 1*σ* observational uncertainty range in globally averaged $\tau_{a}^{\text{PD}}$, while the vertical lines show the resulting 1*σ* range in Δ*τ*_a_ of the constrained PPE.

Most GCMs that simulate aerosol distributions routinely calculate *τ*_a_ for both present‐day and preindustrial conditions. Figure 4 shows the relationship between Δ*τ*_a_ and $\tau_{a}^{\text{PD}}$ in all of the CMIP5 (Taylor et al., 2012) models which participated in the sstClimAerosol experiment, the AeroCom Phase II (Myhre, Samset, et al., 2013) models and the density of 1 million emulated simulations of a PPE using the HadGEM3‐UKCA model to sample uncertainties in 26 physical parameters relating to aerosol processes as well as present‐day and preindustrial emissions (Yoshioka et al., 2019). While there is a large spread in the $\tau_{a}^{\text{PD}}$ (shown in the uppermost panel) in the unconstrained PPE, both multimodel ensembles (MMEs) peak between the lower and upper observational estimates. The MMEs simulate a relationship between $\tau_{a}^{\text{PD}}$ and Δ*τ*_a_, which one would expect on physical grounds from a residence time argument, allowing the observational constraints on $\tau_{a}^{\text{PD}}$ of 0.13 to 0.17 to be translated into a range for Δ*τ*_a_ of 0.03 to 0.04. Sampling only those PPE members, which fall within the observational bounds, leads to a constraint on Δ*τ*_a_ of 0.03 to 0.05. However, one needs to account for the high bias in the default $\tau_{a}^{\text{PD}}$ simulated by the PPE, and a possible high bias in observational estimates, so a range of 0.02 to 0.04 represents a more conservative assessment. By determining the anthropogenic contribution to $\tau_{a}^{\text{PD}}$ in the Monitoring Atmospheric Composition and Climate (MACC) Reanalysis (Benedetti et al., 2009); Bellouin, Quaas, et al. (2013) determine Δ*τ*_a_ as 0.06. The Max Planck Institute Aerosol Climatology (MAC) (Kinne, 2019; Kinne et al., 2013) combines AERONET climatologies with aerosol properties from AeroCom models (Kinne et al., 2006). They report Δ*τ*_a_ as 0.03, which is within the range of the GCM estimates. It should, however, be noted that these estimates rely on the same industrial‐era emissions data sets used in many of the GCM simulations. The larger spread in Δ*τ*_a_ in the PPE is likely due to the fact that it samples uncertainties in these emissions.

Relying on large‐scale models to estimate Δ*τ*_a_ implies that all preindustrial and present‐day sources and sinks of anthropogenic aerosols are represented in these models. There are several reasons that suggest that this is not the case. Potential underestimates of Δ*τ*_a_ come from many GCMs neglecting nitrate aerosols (Myhre, Samset, et al., 2013), which are partly anthropogenic, and having difficulties representing anthropogenic contributions to mineral dust aerosols (Evan et al., 2014). Potential overestimates of Δ*τ*_a_ come from ignoring the possibility that preindustrial fires emitted carbonaceous aerosols at rates similar to present‐day fires (Marlon et al., 2016). In addition, it remains unclear whether biogenic aerosols were more or less prevalent in the preindustrial atmosphere (Ding et al., 2008; Kirkby et al., 2016), and interactions between sulfate aerosol and organic aerosols of biogenic origin may be sizable (Zhu et al., 2019).

Regarding mineral dust aerosols, their anthropogenic component is emitted directly by agriculture and indirectly by soils made more erodible and climate conditions made more erosive by human influence. Estimates of present‐day anthropogenic dust fractions obtained by combining anthropogenic land use data with mineral dust AOD from satellite retrievals range from 8% in North Africa to about 75% in Australia (Ginoux et al., 2012) and China (X. Wang et al., 2018). On a global average, the present‐day anthropogenic mineral dust fraction may be as large as 25% (Ginoux et al., 2010; Huang et al., 2015), translating to an increase in Δ*τ*_a_ of about 0.007, or 15% to 30% of the range of 0.02 to 0.04 obtained above. However, uncertainties on these estimates are large. A few GCM studies yield a range of 10% to 60% for the global average in the anthropogenic fraction of mineral dust for present‐day (Mahowald & Luo, 2003; Stanelle et al., 2014; Tegen et al., 2004), although their simulated changes in anthropogenic mineral dust aerosol disagree in both sign and magnitude (Webb & Pierre, 2018). This disagreement is at least in part caused by differences in simulated meteorological processes (Fiedler et al., 2016). There are uncertainties on mineral dust distributions in 1850 as well, which depend on how vegetation responds to climate changes (Mahowald, 2007). Considered together the contribution of anthropogenic mineral dust aerosols to ERFari is expected to be smaller than for other anthropogenic aerosols, on the order of −0.1 ± 0.2 W m^−2^ (Boucher et al., 2013), owing to compensating contributions of SW scattering and LW absorption. Indeed, Kok et al. (2018) showed that most models underestimate the size of mineral dust aerosols so the compensation between mineral dust SW and LW radiative effects may in fact be stronger than modeled. However, mineral dust aerosols are efficient INPs so anthropogenic mineral dust aerosol potentially alters the radiative properties and life cycle of ice clouds (Gettelman et al., 2012; Kuebbeler et al., 2014; Penner et al., 2018).

Regarding carbonaceous aerosols, emission inventories used by GCMs usually scale fire emissions back to preindustrial levels using historical population changes (e.g., Lamarque et al., 2010), so obtain an increase through the industrial era. Paleoclimate records paint a more complex picture where preindustrial conditions might be more polluted, leading to a smaller Δ*τ*_a_. The synthesis of sedimentary charcoal records by Marlon et al. (2016) suggests a sharp increase in biomass burning from 1800 to 1850, a period of high level of biomass burning from 1850 to 1970, a trough around the year 2000, followed by an abrupt increase up to 2010, although data density is highest in North America and Europe, so those trends may not be globally representative. Still, according to Marlon et al. (2016) present‐day (2010) biomass burning appears larger, on a global scale and for the Northern and Southern Hemispheres individually, than preindustrial if the preindustrial reference year is set at 1750. But choosing a reference year of 1850 means that biomass burning levels were similar to present day. In contrast, van Marle et al. (2017) derive, by merging the satellite record with several existing proxies, including the charcoal records, similar biomass burning emissions between 1750 and 1850, and in their reconstruction, global biomass burning emissions increased only slightly over the full time period and peaked during the 1990s after which they decreased gradually. Hamilton et al. (2018) used significantly revised estimates of preindustrial fires in a single global model study and found an effect of only 10% on RFari. But the impact on CCN was larger, reducing the model's RFaci by 35% to 91% depending on the strength of preindustrial fire emissions.

In summary, a range of 0.02 to 0.04 for Δ*τ*_a_ at 0.55 μm is supported by large‐scale modeling and reanalyses. Combining the range for Δ*τ*_a_ with the range of 0.13 to 0.17 for $\tau_{a}^{\text{PD}}$ yields a range of 0.14 to 0.29 for $\Delta \tau_{a}/\tau_{a}^{\text{PD}}$, meaning that human activities are likely to have increased globally averaged *τ*_a_ by around 15% to 30% in 2005–2015 compared to the year 1850. The range for Δ*τ*_a_ may be too narrow if a large contribution by anthropogenic mineral dust or nitrate aerosols has been overlooked by large‐scale models, or too wide if the atmosphere in year 1850 was significantly more polluted by fire emissions than currently thought. Although those differences may not always affect globally averaged ERFari on account of the absorbing properties of the aerosols involved, they could lead to sizable changes in ERFaci.

## 5 RF of Aerosol‐Radiation Interactions

**Figure 5:**
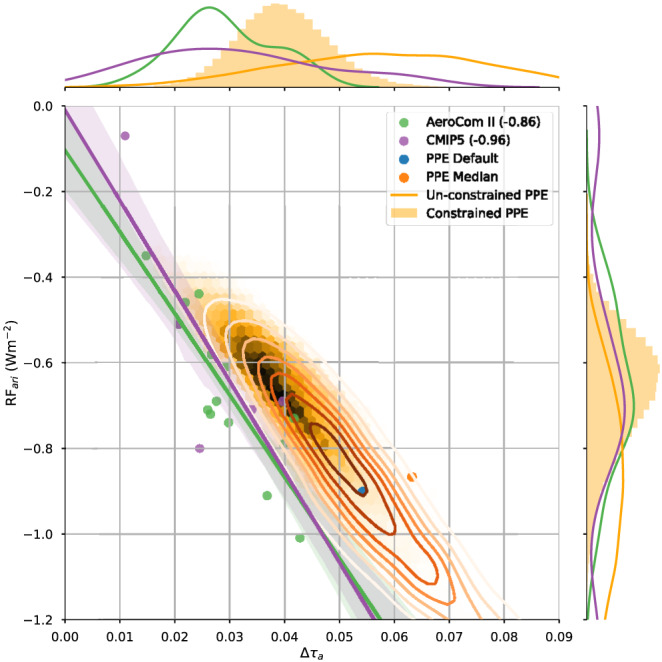
Clear‐sky radiative forcing of aerosol‐radiation interactions, RFari in W m^−2^, as a function of the industrial‐era change in aerosol optical depth at 0.55 μm, Δ*τ*_a_ in AeroCom models (green), CMIP5 models (purple). The slopes of the lines of best fit for each data set are −19.1 and −21 W m^−2^ $\tau_{a}^{-1}$, respectively. The joint distribution of the full emulated HadGEM‐UKCA 26 aerosol parameter Perturbed Physics Ensemble (PPE) is shown with contours, while the samples consistent with $\tau_{a}^{\text{PD}}$ is shown as a hex density. The slope for the PPE is −14 W m^−2^ $\tau_{a}^{-1}$. The default and median model runs are also shown for completeness. The 1*σ* uncertainty in the fits are shaded and the correlation coefficients are indicated in the parentheses in the legend.

As stated in section 2.1, efficiency factors for scattering and absorption per unit AOD depend on a wide array of physical and chemical properties of the aerosols. In light of that complexity, the good agreement in clear‐sky sensitivities $S_{\tau }^{\text{clear}}=\partial R_{\text{clear}}/\partial \tau_{a}$ among AeroCom models is remarkable, with Myhre, Samset, et al. (2013) reporting in their Table 3 a value of −23.7 ± 3.1 W m ${}^{-2}\;\tau_{a}^{-1}$ (neglecting an anomalous outlier because the causes for its very strong clear‐sky RFari are not understood) or, if sensitivities are expressed in terms of planetary albedo, a range for $S_{\tau }^{\text{clear}}$ from 0.06 to 0.08 $\tau_{a}^{-1}$. Clear‐sky RFari against Δ*τ*_a_ is shown in Figure 5 for two multimodel ensembles and a large single‐model PPE. While there is a large spread in the absolute values of RFari, particularly in the PPE which was designed to explore the full range of parametric uncertainty in HadGEM3 and is unconstrained by observations here, the slope, which is the sensitivity *S*_*τ*_, is similar between the multimodel and perturbed parameter ensembles.

Uncertainties in the retrieval of *τ*_abs_ are much larger than for *τ*_a_ and contribute to the spread in $S_{\tau }^{\text{clear}}$ seen in Figure 5. The absorption of seasalt and sulfate aerosols is well constrained, but the absorption of mineral dust and carbonaceous aerosol is poorly characterized. Bond et al. (2013) noted that AeroCom models underestimate *τ*_abs_ compared to AERONET so they proposed increasing emissions of absorbing BC aerosols in response. They estimated that the present‐day anthropogenic *τ*_abs_ from carbonaceous aerosols is about 0.007 at 0.55 μm. Bellouin, Quaas, et al. (2013) also used AERONET to prescribe aerosol absorption and reached a similar estimate based on a reanalysis of atmospheric composition. But more recent studies challenged the need for scaling of models and the suitability of AERONET constraints, instead improving modeled BC by increasing the model horizontal resolution (X. Wang et al., 2014), reducing BC lifetime (Samset et al., 2014) to reduce overestimations of BC concentrations in remote areas (Kipling et al., 2013), or accounting for AERONET *τ*_abs_ sampling errors (X. Wang et al., 2018) and possible high bias compared to in situ airborne absorption coefficients (Andrews et al., 2017). Compared to Kinne (2019), Bond et al. (2013) overestimated anthropogenic *τ*_abs_ because they underestimated the contribution of mineral dust aerosol to *τ*_abs_ and overestimated the anthropogenic fraction of BC aerosols. The revised calculation by Kinne (2019) therefore motivates a downward revision of *τ*_abs_ at 0.55 μm, with a range of 0.0025 to 0.005. Rapid adjustments due to anthropogenic absorption are discussed in section 7.

Extending $S_{\tau }^{\text{clear}}$ to all‐sky conditions requires accounting for masking by clouds above aerosol but also aerosol absorption enhancement when clouds are below the aerosol (Figure 3). According to AeroCom models, those situations only contribute a small forcing on a global average, with a distribution centered around 0 W m^−2^ (Schulz et al., 2006). Studies based on CALIOP estimate a positive aerosol radiative effect above clouds (Chand et al., 2009; Kacenelenbogen et al., 2019; Oikawa et al., 2018), resulting from a partial compensation of a positive radiative effect by smoke aerosols with a negative radiative effect from mineral dust aerosols, although the anthropogenic fraction of those aerosols and the resulting cloudy‐sky RFari is unknown. In addition, CALIOP underestimates aerosols at altitudes above 4 km (Watson‐Parris et al., 2018). Although the regional cloudy‐sky radiative effects of ari can be strongly positive (de Graaf et al., 2014; Keil & Haywood, 2003; Peers et al., 2015), a small globally averaged cloudy‐sky RFari is expected because most of anthropogenic aerosols are located in the planetary boundary layer, where their RFari is masked by dense water clouds or partially masked by ice clouds. Indeed, GCMs tend to put too much aerosol mass aloft compared to CALIOP vertical aerosol extinction profiles (Koffi et al., 2016), so even the small cloudy‐sky RFari reported by Schulz et al. (2006) may be an overestimate. Based on the results of Schulz et al. (2006), $S_{\tau }^{\text{cloudy}}$ may be as small as ±0.02 $\tau_{a}^{-1}$. That small efficiency coupled with the regional and seasonal nature of occurrences of anthropogenic aerosols above clouds suggest that all‐sky *S*_*τ*_ is approximately equal to $S_{\tau }^{\text{clear}}$ weighted by an effective clear‐sky fraction, and that cloudy‐sky forcing only adds an uncertainty of ±0.1 W m^−2^ (Schulz et al., 2006).

This effective clear‐sky fraction is the complement of the effective cloud fraction for ari, noted *c*_*τ*_ in equation (8). *c*_*τ*_ is the convolution of the cloud fraction C and cloud optical depth, *τ*_c_, to account for situations where clouds are too thin to completely mask the RFari of aerosols located below them, and Δ*τ*_a_, to account for the different distributions of anthropogenic aerosols and low clouds. This gives

(9) $$c_{\tau }=\frac{\left\langle \tau_{c}\;\Delta \tau_{a}\;C\right\rangle }{\left\langle \tau_{c}\;\Delta \tau_{a}\right\rangle }$$

where angle brackets denote global‐area‐weighted temporal averaging. Stevens (2015) finds *c*_*τ*_=0.65, which is close to the mean *c*_*τ*_ of 0.66 obtained by the nine global aerosol‐climate models that participated in Zhang et al. (2016) (Table 3). Those models give a standard deviation for *c*_*τ*_ of 0.06. Because large‐scale models tend to have similar geographical distributions of anthropogenic aerosols, differences primarily stems from different liquid cloud climatologies in AeroCom models. GCMs are known to underrepresent low‐level cloudiness (Nam et al., 2012), so may underestimate *c*_*τ*_ by simulating the wrong spatial patterns of C.

**Table 3:** Estimates of Effective Cloud Fraction for Aerosol‐Radiation Interactions, *c*_*τ*_, in the Nine Global Aerosol‐Climate Models That Participated in Zhang et al. (2016)

Model name	Reference	*c*_*τ*_
CAM5.3_CLUBB	Bogenschutz et al. (2013)	0.693
ECHAM6‐HAM2	Neubauer et al. (2014)	0.552
GEOS‐5	Barahona et al. (2014)	0.596
HadGEM3‐A‐GLOMAP	Bellouin, Mann, et al. (2013)	0.728
ModelE2‐TOMAS	Lee et al. (2015)	0.667
NCAR_CAM5.3_CLUBB_MG2		0.680
NCAR_CAM5.3_MG2	Gettelman and Morrison (2015)	0.637
NCAR_CAM5.3	Liu et al. (2012)	0.673
SPRINTARS	Takemura et al. (2005)	0.704
SPRINTARS_KK	Takemura et al. (2005)	0.697
	*Mean*	*0.663*
	*Median*	*0.677*
	*Standard deviation*	*0.054*

*Note*. Models that share the same host model use different aerosol and/or cloud schemes.

In summary, the RF of ari, Fari, is computed as in equation 8, $\Delta \tau_{a}\left[S_{\tau }^{\text{clear}}\left(1-c_{\tau }\right)+S_{\tau }^{\text{cloudy}}\;c_{\tau }\right]$ where the last term represents the contribution of cloudy‐sky ari. The ranges adopted for the terms of this equation are:

- 0.02 to 0.04 for Δ*τ*_a_, as obtained by section 4;
−20 to −27 W m ${}^{-2}\;\tau_{a}^{-1}$ for $S_{\tau }^{\text{clear}}$, rounding outwards the range simulated by AeroCom models used by Myhre, Samset, et al. (2013). $S_{\tau }^{\text{clear}}$ can also be expressed in terms of planetary albedo by dividing by the globally and annually averaged solar constant of 340 W m^−2^: The range becomes 0.06 to 0.08 $\tau_{a}^{-1}$;
- 0.59 to 0.71 for *c*_*τ*_, rounding outwards the range simulated by AeroCom models used by Zhang et al. (2016) (Table 3);
- 0.0 ± 0.1 W m^−2^ for the product $S_{\tau }^{\text{cloudy}}\;c_{\tau }$, as simulated by AeroCom models (Schulz et al., 2006).

Using the method described in section 2.2 to combine those ranges and compute the first term of equation (8) yields a range for Fari of −0.37 to −0.12 W m^−2^. The rapid adjustments due to anthropogenic absorption are discussed separately in section 7.

## 6 RF of Aerosol‐Cloud Interactions in Liquid Clouds

Since liquid clouds and ice clouds behave differently in several aspects, it is useful to distinguish between the two. Clouds with a cloud top temperature warmer than 0 °C are liquid. Clouds colder than this behave in the same way in terms of the mechanisms that determine RFaci if they consist of supercooled liquid water but behave differently when ice becomes present. The three key bulk quantities that describe the properties of a liquid cloud are their LWP, L, their cloud fraction, C, and their droplet number concentration, *N*_d_.

Cloud droplets are formed via adiabatic cooling of air parcels by updrafts that generate supersaturation, and each droplet forms on an aerosol particle that serves as a CCN at the supersaturation determined by the cooling rate. Which aerosols are activated into cloud droplets depends on the size of the particles and their hygroscopicity (Köhler, 1936), as well as on the maximum supersaturation that is reached given the balance between adiabatic cooling due to the updraft that increases supersaturation, and condensation of vapor onto the droplets that reduces it (Twomey, 1959). In consequence, additional aerosol leads to further cloud droplets if they are large enough compared to the preexisting aerosol population. On average, at the scale of an air parcel, an approximately logarithmic scaling between aerosol concentration and *N*_d_ is obtained (Twomey, 1959). At the cloud scale, it is therefore sufficient to know the aerosol size distribution and hygroscopicity, as well as the updraft distribution, to predict *N*_d_ as well as its sensitivity to the aerosol. A relative change of *N*_d_ in response to an aerosol, *a*, perturbation is thus

(10) $$\beta_{\ln N-\ln a}=\frac{\partial \ln N_{d}}{\partial \ln a}$$

The aerosol metric *a* is left ambiguous here, since in different observations‐based studies, different choices are made. The optimal definition would be the CCN concentration at cloud base, but for many observations (e.g., remote sensing), this quantity is not accessible. The sensitivity $\beta_{\ln N-\ln a}$ is often evaluated using linear regressions, with various choices for the aerosol metric *a* (Feingold et al., 2003; McComiskey et al., 2009). For large updrafts and suitable aerosol, at relatively low background aerosol concentration, such as found for remote marine trade‐wind cumulus, a sensitivity approximately equal to unity is observed with CCN as aerosol metric (Martin et al., 1994; Twohy et al., 2005; Werner et al., 2014). For more general situations, including smaller updraft speeds, higher CCN concentrations, and broader aerosol size distributions, the scaling between aerosol concentration and *N*_d_ is substantially lower (e.g., Boucher & Lohmann, 1995; Lu et al., 2009). McFiggans et al. (2006) explore the sensitivity from parcel modeling to obtain values between 0.7 and 0.9. Surface remote sensing statistics yield a range of 0.3 to 0.5 for a coastal site (McComiskey et al., 2009), midlatitude continental sites (Kim et al., 2008; Schmidt et al., 2015) and the Arctic (Garrett et al., 2004). Values can be larger—up to 0.75—when sampling updraft conditions only (Schmidt et al., 2015). Painemal and Zuidema (2013) obtain values as large as 0.8 to 0.9 when combining in situ aerosol observations with aircraft remote sensing for *N*_d_ over the Southeast Pacific Ocean.

A wide range of observational evidence from ship tracks, trends in anthropogenic emissions, and degassing volcanic eruptions (Gassó, 2008; Christensen & Stephens, 2011; Yuan, Remer, Pickering, & Yu, et al., 2011; Christensen et al., 2014; McCoy & Hartmann, 2015; Malavelle et al., 2017; Toll et al., 2017; McCoy, Field, et al., 2018; Li et al., 2018) and numerous field studies in different regions (Boucher & Lohmann, 1995; Lowenthal et al., 2004; Rosenfeld et al., 2008; Werner et al., 2014) support the theoretical argument that the impact of additional aerosols in the atmosphere is to increase *N*_d_, and decrease the cloud effective radius as the liquid water is spread among a larger number of droplets (Twomey, 1977). But quantifying those relationships is difficult and depends on cloud regime.

For global coverage, the sensitivity of *N*_d_ to aerosol can only be assessed from satellite retrievals (Nakajima et al., 2001; *Lohmann and Lesins*, 2002; Sekiguchi et al., 2003; *Quaas et al*., 2006). However, such assessments suffer from a number of problems.

1. Aerosol and cloud quantities usually cannot be retrieved in the same column. It is thus unclear to what extent the aerosol retrieved in clear‐sky pixels is representative of the aerosol relevant for cloud droplet formation (e.g., Gryspeerdt et al., 2015). Even if in general, the horizontal scale of variance of the aerosol is large compared to that of clouds (Anderson, Charlson, Winker, et al., 2003), this assumption may be weak in the proximity of precipitating clouds. In addition, sampling the aerosol radiative properties in close vicinity to clouds leads to errors (Christensen et al., 2017) due to the humidity swelling of the aerosol (Quaas et al., 2010) and misclassification of cloud as aerosols (Zhang et al., 2005).
2. The most straightforward remote sensing aerosol retrieval is the AOD, *τ*_a_. However, *τ*_a_ does not scale very well with the relevant CCN concentration at cloud base, because it is a column‐integrated quantity, is affected by humidity, and by aerosols that may not act as CCN (Stier, 2016). Errors in retrieved *τ*_a_ are also largest at small values where cloud sensitivity may be largest (Ma et al., 2018). The aerosol index, calculated by multiplying *τ*_a_ by a measure of aerosol size, is often suggested as an approximate solution to provide a possibly better indicator of CCN concentrations (Gryspeerdt et al., 2017; Penner et al., 2011; Stier, 2016), but at low aerosol loadings uncertainties in aerosol index are even larger than for *τ*_a_.
3. Cloud droplet number concentration is derived from retrievals in a very indirect way, which relies on cloud top quantities and assumptions to extrapolate down to cloud base where equation (10) applies. Depending in particular on cloud heterogeneity and solar zenith angle, retrievals may be strongly biased (Grosvenor et al., 2018). The retrieved *N*_d_ does not directly correspond to the activated droplet concentration near cloud base but is the result of both cloud microphysical processes and cloud entrainment mixing processes. Aggregation to relatively coarse retrieval scales reduces the representativeness of the sensitivity of *N*_d_ to the aerosol because important process‐level scales are not captured (McComiskey & Feingold, 2012).

From satellite remote sensing, thus, the sensitivity of *N*_d_ to aerosol is often estimated by evaluating equation (10) using *τ*_a_ as the aerosol metric. Most of the caveats listed above, except for the increased *τ*_a_ when considering retrievals within approximately 15 km of nearby clouds (Christensen et al., 2017), lead to too weak sensitivities when retrieving the *N*_d_—*τ*_a_ relationship. Making use of satellite‐based statistics to quantify $\beta_{\ln N-\ln\tau_{a}}$ usually yield much smaller values than those derived from airborne measurements (McComiskey & Feingold, 2012; McCoy et al., 2017; Nakajima & Schulz, 2009; Schmidt et al., 2015). The full range for $\beta_{\ln N-\ln a}$, using different aerosol quantities for *a*, compiled by those studies spans 0.14 to 1.00. However, local sensitivities need to be weighted globally to be relevant for the large‐scale forcing. The newest compilation of large‐scale sensitivities by McCoy et al. (2017) obtains (their Figure 3) a range of 0.3 to 0.8. It is difficult to rigorously assign confidence intervals, in particular because the physically meaningful range is bounded. Nevertheless, if one considers the full range of 0.14 to 1.00 from the four studies cited above as the 90% confidence interval, one obtains a ±*σ* interval of 0.52, which matches the interval obtained by McCoy et al. (2017).

Combining the range of 0.3 to 0.8 for $\beta_{\ln N_{d}-\ln\tau_{a}}$ with the ranges of 0.02 to 0.04 for Δ*τ*_a_ and 0.13 to 0.17 for *τ*_a_ obtained in section 4 yields a range of 0.05 to 0.17 for $\Delta \ln N_{d}$, encompassing the estimate of 0.15 obtained by Charlson et al. (1992) and Stevens (2015).

The dependency of cloud reflectance on the bulk cloud properties *N*_d_ and L is based on their relationship with cloud optical depth, *τ*_c_, assuming adiabatic clouds (e.g., Brenguier et al., 2000):

(11) $$\tau_{c}\propto L^{\frac{5}{6}}\;N_{d}^{\frac{1}{3}}$$

(12) $$d\ln\tau_{c}=\frac{5}{6}\;d\ln L+\frac{1}{3}\;d\ln N_{d}$$

Further, variations in cloud albedo, *α*_c_, are related to variations in *τ*_c_ approximately as (Ackerman et al., 2000)

(13) $$d\alpha_{c}=\alpha_{c}\left(1-\alpha_{c}\right)d\ln\tau_{c}=\alpha_{c}\;\left(1-\alpha_{c}\right)\;\left(\frac{5}{6}\;d\ln L+\frac{1}{3}\;d\ln N_{d}\right)$$

If dlnL is set to 0, the RF of the Twomey effect, Faci, is isolated. According to equation (8), Faci is also:

(14) $$F\text{aci}=\Delta \ln N_{d}\;S_{N}\;c_{N}$$

The anthropogenic perturbation of droplet number concentration is estimated from the sensitivity of *N* to aerosol perturbations, and the relative perturbation in aerosol, $\Delta \ln N_{d}=\beta_{\ln N-\ln a}\Delta \ln a$. If *τ*_a_ is chosen to quantify the aerosol, $\Delta N_{d}=\beta_{\ln N-\ln a}\frac{\Delta \tau_{a}}{\tau_{a}}$, leading to the equation:

(15) $$F\text{aci}=\beta_{\ln N-\ln a}\frac{\Delta \tau_{a}}{\tau_{a}}\;S_{N}\;c_{N}$$

For reference, Table 2 summarizes the definitions of the variables used in equation (15).

Since the Twomey effect has little impact on $R_{\text{LW}}^{↑}$, *S*_N_ can be redefined for convenience as the sensitivity of the planetary albedo with respect to *N*_d_ perturbations:

(16) $$S_{N}=\frac{\partial \alpha }{\partial \ln N_{d}}$$

Inserting equation (13) into equation (16) yields (Twomey, 1977):

(17) $$S_{N}=\frac{1}{3}\;\alpha_{c}\;\left(1-\alpha_{c}\right)$$

The global mean cloud albedo is quantified from the CERES SSF1deg Ed4A (Loeb et al., 2016) at *α*_c_ = 0.38 ± 0.02, evaluated as the planetary albedo at 1°×1° grid boxes where the fractional coverage by liquid water clouds is larger than 95%. Propagating the uncertainty in *α*_c_ to *S*_N_ using equation (17) yields a range for *S*_N_, as defined by equation (16), of 0.077 to 0.080. In equations (6) and (14), *c*_N_ is an effective cloud fraction. It is “effective” because it is not just the fractional coverage by liquid water clouds, $C_{\text{liq}}$, as retrieved from satellite data, that would be the relevant quantity at a given location in space and time (e.g., Quaas et al., 2008). Instead, it also takes into account the spatial covariability of the other terms relevant to deriving RFaci. *c*_N_ is needed because equation (13) is a global mean equation. In essence, *c*_N_ is the spatiotemporally resolved Faci, normalized by the global‐temporal averages of the first four terms on the right‐hand side of equation (13):

(18) $$c_{N}=\frac{\left\langle C_{\text{liq}}\;\alpha_{c}\left(1-\alpha_{c}\right)\;\beta_{\ln N-\ln\tau_{a}}\;\frac{\Delta \tau_{a}}{\tau_{a}}\;R_{\text{SW}}^{↓}\right\rangle }{\left\langle \alpha_{c}\;\left(1-\alpha_{c}\right)\rangle \langle \beta_{\ln N-\ln\tau_{a}}\rangle \langle \frac{\Delta \tau_{a}}{\tau_{a}}\rangle \langle R_{\text{SW}}^{↓}\right\rangle }$$

where angle brackets denote global‐area‐weighted temporal averaging of two‐dimensional distributions. In other words, it is the fractional coverage of liquid clouds weighted by:

- the sensitivity of cloud albedo to perturbations in *N*_d_, *S*_N_;
- the local sensitivity of *N*_d_ to perturbations in aerosol, $\beta_{\ln N_{d}-\ln\tau_{a}}$
- the occurrence of anthropogenic perturbations to the aerosol, $\frac{\Delta \tau_{a}}{\tau_{a}}$; and
- the incoming solar radiation.

The range in $\beta_{\ln N_{d}-\ln\tau_{a}}$, 0.3 to 0.8, is taken from McCoy et al. (2017) and the range for Δ*τ*_a_ is that spanned by Bellouin, Quaas, et al. (2013) and Kinne (2019).

**Figure 6:**
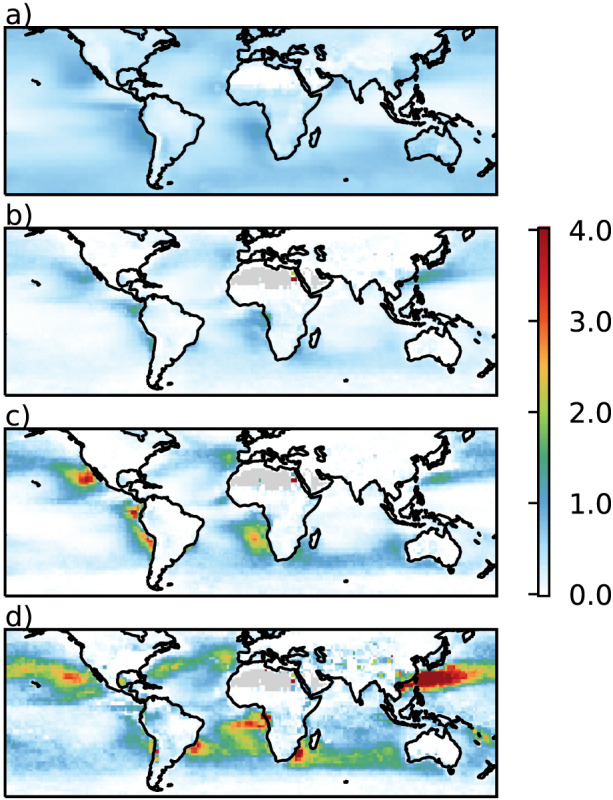
(a) Liquid cloud fraction *C*_liq_, multiplied by 2 to be legible on the shared color scale. (b–d) The effective cloud fractions for b) the radiative forcing of aerosol‐cloud interactions (*c*_*N*_), c) rapid adjustments in liquid water path ( $c_{L}$) and d) rapid adjustments in liquid cloud fraction (c ${}_{C}$). Distributions have been calculated using cloud retrievals by MODIS (Platnick et al., 2017), CERES cloud albedo (Wielicki et al., 1996), and the anthropogenic aerosol fraction from Bellouin, Quaas, et al. (2013).

The local sensitivity $\beta_{\ln N_{d}-\ln\tau_{a}}$ is calculated using MODIS collection 6 cloud droplet number concentration, sampled following Grosvenor et al. (2018) and the MODIS AOD (Levy et al., 2013). The Δ*τ*_a_/*τ*_a_ used are from Bellouin, Quaas, et al. (2013). Although the magnitude of $\beta_{\ln N_{d}-\ln\tau_{a}}$ calculated using this method is an underestimate (Penner et al., 2011), *c*_N_ only depends on its spatial pattern. To obtain an uncertainty range in *c*_N_, alternative spatial distributions for $\beta_{\ln N_{d}-\ln\tau_{a}}$ are taken from McCoy et al. (2017) and for Δ*τ*_a_/*τ*_a_ from Kinne (2019), yielding a range for *c*_N_ of 0.19 to 0.29. The value of 0.1 used in Stevens (2015) is therefore outside the 68% confidence interval obtained here, but he was likely referring to marine stratocumulus clouds, while the present range encompasses all liquid clouds. Figure 6 illustrates the geographical distribution of *c*_N_ as defined in equation (18) but averaging the numerator only in time, not in space. Compared to *C*_liq_, the distribution of *c*_N_ emphazises low maritime clouds, and especially stratocumulus decks, which are most sensitive to aerosol perturbations (Alterskjær et al., 2012; Oreopoulos & Platnick, 2008). The tendency of *C*_N_ to be larger than *C*_liq_ is expected due to spatial correlations between *C*_liq_ and β_ln Nd−ln τa_ (Gryspeerdt & Stier, 2012).

In summary, calculating $\Delta \ln N_{d}$ as $\Delta \tau_{a}/\tau_{a}^{\text{PD}}\;\beta_{\ln N_{d}-\ln\tau_{a}}$, where $\beta_{\ln N_{d}-\ln\tau_{a}}=\partial \ln N_{d}/\partial \ln\tau_{a}$, yields a range of 0.05 to 0.17, based on the ranges of

- 0.02 to 0.04 for Δ*τ*_a_ and 0.13 to 0.17 for $\tau_{a}^{\text{PD}}$, following section 4;
- 0.3 to 0.8 for $\beta_{\ln N_{d}-\ln\tau_{a}}$, following McCoy et al. (2017);

Note that McCoy et al. (2017) infer the sensitivity from sulfate mass concentration rather than AOD. Their sensitivity is therefore used here by assuming that the relative perturbation in anthropogenic AOD is proportional to the perturbation in anthropogenic sulfate mass concentration.

The range for $\Delta \ln N_{d}$ means that human activities are likely to have increased globally averaged cloud droplet number concentrations by 5 to 17% in 2005–2015 compared to the year 1850. The RF of aci, Faci, is computed following equation (14). The ranges adopted for the terms of this equation are as follows:

- 0.05 to 0.17 for $\Delta \ln N_{d}$, as above;
- 0.077 to 0.080 for *S*_*N*_, based on uncertainties in *α*_*c*_ from CERES. That range converts to a range of −26 to −27 W m^−2^ in terms of top‐of‐atmosphere radiation: The conversion is done by multiplying by the global, annual mean incoming solar radiation of 340 W m^−2^;
- the range of 0.19 to 0.29 for *c*_N_.

Using the method described in section 2.2 to combine those ranges and solve equation 15, the range for Faci is −1.10 to −0.33 W m^−2^. Rapid adjustments to aci are quantified separately in section 8.

## 7 Rapid Adjustments to Aerosol‐Radiation Interactions

Both d*R*/d*R*_atm_ and d*R*_atm_/d*τ*_a_ of equation (8) have been found to depend on the amount and altitude of absorbing aerosols and the location of those aerosols relative to the clouds by large‐eddy simulations (Johnson et al., 2004), global modeling (Hansen et al., 2005; Penner et al., 2003), and observations (Koren et al., 2004). These findings were summarized into frameworks where the sign of the adjustments depends on the cloud regime and whether aerosols are below, in, or above the clouds (Bond et al., 2013; Koch & Del Genio, 2010), although only a handful of studies were available to illustrate each case. When absorbing aerosol lies within the boundary layer, the RF is positive while when it lies above the boundary layer it is negative. Assessments based on large‐scale modeling, like Boucher et al. (2013), conclude that the rapid adjustments to ari operating via changes in cloud properties exert a negative RF on a global average, of the order of −0.1 W m^−2^, suggesting a dominance of absorbing aerosol above clouds. Large eddy simulation (LES) modeling of semidirect effects suggests a positive RF from convective cloud suppression when absorbing aerosol lies within the boundary layer (Feingold et al., 2005) and positive again when absorbing aerosol lies above stratocumulus clouds (Yamaguchi et al., 2015). The latter study showed a delay of the stratocumulus to cumulus transition, complementing observations by Adebiyi et al. (2015). That delay could be associated with a locally large negative RF. But as discussed in section 3.2.1, scaling those results to a global radiative sensitivity is challenging.

In contrast to RFari, a substantial fraction of the rapid adjustments happens in the LW spectrum (Penner et al., 2003). The Precipitation Driver Response Model Intercomparison Project (PDRMIP) (Myhre et al., 2017) focused on rapid adjustments in clouds, but also on the contribution stemming from altered tropospheric temperature and water vapor profiles. Smith et al. (2018) found that rapid adjustments associated with temperature changes in the troposphere and stratosphere and those due to water vapor changes are comparable in magnitude to the rapid adjustments in clouds. Again, most of the rapid adjustments occur in the LW spectrum. The PDRMIP results indicate that the total rapid adjustment represents about half of the strength of the RFari by BC aerosols. Scaling the PDRMIP results to current estimate of global anthropogenic emission of BC (Hoesly et al., 2018) would give a total rapid adjustment due to BC of about −0.2 W m^−2^. However, the PDRMIP results are based on global models that may overestimate the lifetime of BC aerosols and their concentrations aloft. A shorter BC lifetime, in better agreement with observations in the middle and upper troposphere, would reduce the magnitude of the rapid adjustment but would also reduce the BC RFari (Hodnebrog et al., 2014).

PDRMIP models find a total rapid adjustment of −1.3 W m^−2^ for an instantaneous change in atmospheric absorption of +6.1 W m^−2^ (Supplementary Tables 1 and 2 of *Myhre et al.*, 2018), leading to a mean d*R*/d*R*_atm_=−0.2, with a standard deviation of 0.09. The RF exerted within the atmosphere per unit anthropogenic *τ*_a_ is generally small, except over regions and during seasons where the amount of absorbing aerosol is large. On a global, annual average basis, Bellouin, Quaas, et al. (2013) find d*R*_atm_/d*τ*_a_=+41 W m ${}^{-2}\;\tau_{a}^{-1}$. This is at the higher end of the range obtained by AeroCom models, which span +13 to +47 W m ${}^{-2}\;\tau_{a}^{-1}$, with a median of +26 W m ${}^{-2}\;\tau_{a}^{-1}$ and a standard deviation of 9 W m ${}^{-2}\;\tau_{a}^{-1}$ (Myhre, Samset, et al., 2013).

In summary, rapid adjustments of ari are computed using the second term of equation (8), Δ*τ*_a_d*R*/d*R*_atm_d*R*_atm_/d*τ*_a_. The ranges adopted for the terms of this equation are:

- 0.02 to 0.04 for Δ*τ*_a_, as obtained by section 4;
−0.1 to −0.3 for d*R*/d*R*_atm_ based on PDRMIP simulations reported by Myhre et al. (2018)
- 17 to 35 W m ${}^{-2}\;\tau_{a}^{-1}$ for d*R*_atm_/d*τ*_a_, based on AeroCom simulations reported by Myhre, Samset, et al. (2013).

Using the method described in section 2.2 to combine those ranges yields a range for the rapid adjustments to ari of −0.06 to −0.25 W m^−2^. The range for Eari is obtained by adding, with the method described in section 2.2 again, the range for rapid adjustments to the range of −0.12 to −0.37 W m^−2^ obtained for Fari in section 5. Doing so yields a range of −0.23 to −0.58 W m^−2^ for Eari. Note that Fari and its rapid adjustments are correlated, at least in the framework of equation (8), through Δ*τ*_*a*_.

## 8 Rapid Adjustments to Aerosol‐Cloud Interactions

The change in *N*_d_ due to aerosols that drives the Twomey effect may also impact cloud droplet size and so modify cloud processes (Ackerman et al., 2004; Albrecht, 1989). While the RF of the Twomey effect is formulated in terms of a constant L, a change to cloud processes may be able to modify L and C, possibly generating a significant RF (Albrecht, 1989; Pincus & Baker, 1994). This section concentrates on liquid cloud adjustments. Similar rapid adjustments in response to aerosol perturbations in mixed‐phase and ice clouds may also produce a sizable RF (Lohmann, 2002; Lohmann, 2017; Storelvmo, 2017; Storelvmo et al., 2008) but are covered by section 9 because different processes are involved and the level of scientific inquiry is less advanced. The present section also considers constraints on rapid adjustments in L and C separately, following equation (8). This separation allows a better comparison with the observational studies that adopted an approach where a system‐wide variable, the cloud radiative effect, is used to compute ERFaci. Those studies treat “intrinsic” (changes in cloud albedo), and “extrinsic” (changes in C) effects separately (e.g., Chen et al., 2014). Doing so reduces the number of free parameters to just a few (e.g., C, *α*_c_, and *τ*_a_) in which the observational uncertainties are better known than for *N*_d_ and L. It also has a closer correspondence to the internal structure of many GCMs, where L and C are treated by different parametrizations, even though the liquid cloud adjustments are usually parameterized through modification of the autoconversion rate (e.g. *Khairoutdinov and Kogan*, 2000), which is the rate at which cloud water becomes rain water. The intrinsic/extrinsic methodology closely agrees with earlier methods (e.g. Quaas et al., 2008), as shown by Amiri‐Farahani et al. (2017) and Christensen et al. (2017).

### 8.1 Adjustments in LWP L

The sensitivity of L to *N*_d_ varies regionally (Han et al., 2002) and is expected to depend on the relative magnitude of two key processes (Lohmann & Feichter, 2001). The suppression of precipitation from a reduction in droplet size could increase L (Albrecht, 1989), while radiation, evaporation, and sedimentation enhance cloud top turbulence and increase cloud top cooling, enhancing the entrainment of dry air, resulting in a reduction in L in polluted regions (Ackerman et al., 2004; Bretherton et al., 2007; S. Wang et al., 2003). The overall sensitivity of L to aerosol is strongly modulated by meteorology, affecting the relative importance of each process (Chen et al., 2014; Christensen et al., 2017; Gryspeerdt, Goren, et al., 2019; Michibata et al., 2016; Neubauer et al., 2017), which will be different in different cloud regimes.

Satellite studies have shown a close relationship between cloud droplet size and precipitation in warm clouds, with smaller droplets inhibiting precipitation formation (Rosenfeld & Ulbrich, 2003; Suzuki et al., 2013). A strong positive $\beta_{\ln L-\ln N_{d}}$ (= $\partial \ln L/\partial \ln N_{d}$ following the definition of $\beta_{\ln N_{d}-\ln\tau_{a}}$) is found in precipitating clouds (Chen et al., 2014), suggesting that precipitation suppression can increase L. With a parametrized impact of *N*_d_ on only the autoconversion rate, many GCMs produce an increase in L with increasing aerosol (Quaas et al., 2009), resulting in a negative RF that enhances the overall ERFaci[liquid] in some models by around 30% (Gettelman, 2015). However, comparisons of GCM results to cloud perturbations due to shipping and volcanic aerosol support a weaker L adjustment on a global average (Malavelle et al., 2017; Toll et al., 2017).

The tendency of GCMs to form light precipitation too frequently may lead to an overly strong impact of precipitation suppression (Stephens et al., 2010; M. Wang et al., 2012), as aerosols cannot suppress precipitation from a nonprecipitating cloud (Sorooshian et al., 2009). Precipitation processes in GCMs have been shown to be less sensitive to aerosol than in observations (Jing & Suzuki, 2018), although observations can easily confuse cause and effect, so that scavenging may in fact not be sufficiently active in GCMs. In any case, the size of the L‐*N*_d_ sensitivity component driven by precipitation suppression is still uncertain. Despite this, GCMs rarely produce an enhancement of the RFaci larger than 50% due to changes in L (Gryspeerdt, Mülmenstädt, et al., 2019).

Satellites often observe a strong negative $\beta_{\ln L-\ln N_{d}}$, particularly in regions of low cloud top humidity (Chen et al., 2014; Michibata et al., 2016), which may be driven by aerosol‐dependent cloud top entrainment, and might also not respect assumptions made by the retrievals on the adiabatic nature of the clouds. It might also be a manifestation of reductions in *N*_d_ due to precipitation formation in clouds with elevated L. The relationship is reproduced by global cloud‐resolving simulations (Sato et al., 2018). The possible decrease in L due to this effect is therefore not well constrained and generally not included in the GCM studies cited above. Gryspeerdt, Goren, et al. (2019) find values of $\beta_{\ln L-\ln N_{d}}$ as negative as −0.4, but note that this is likely an overestimate due to the impact of meteorological covariations, with a value closer to −0.1 being in better agreement with Ackerman et al. (2004) and results from natural experiments. A conservative lower bound of −0.36 is chosen, based on Figure 2f of Gryspeerdt et al. (2019). Toll et al. (2017) find a value of −0.011, which is the least negative number that is based on large‐scale aggregate observations with plausible evidence for causality in the L – *N*_d_ relationship. It is thus taken as an upper bound for this adjustment, since positive values, although possible in individual clouds, are unlikely to hold on average according to the analyses of ship, volcano, and pollution tracks by Toll et al. (2017) and Toll et al. (2019).

### 8.2 Adjustments in Cloud Cover C

The suppression of precipitation may also lead to a change in C, either via increases in cloud lifetime (Albrecht, 1989) or by affecting the transition between closed‐ and open‐celled stratocumulus (Rosenfeld, 2006). Many studies have observed links between C and aerosol radiative properties, especially *τ*_a_, finding both increases and decreases in C with increasing aerosol (Dey et al., 2011; Gryspeerdt, Stier, & Partridge, 2014; Kaufman & Koren, 2006; Kaufman et al., 2005; Loeb & Schuster, 2008; Sekiguchi et al., 2003; Small et al., 2011; Yuan, Remer, & Yu, 2011). However, it has proved challenging to separate the role of aerosols from the impact of retrieval biases (Brennan et al., 2005; Várnai & Marshak, 2009) and meteorological covariations (Chand et al., 2012; Grandey et al., 2013; Quaas et al., 2010).

GCMs typically show an increase in C and a corresponding negative rapid adjustment in response to aerosol (Ghan et al., 2016; Zelinka et al., 2014), due to the aerosol impact depending indirectly on the aerosol‐driven reduction in autoconversion. Simulating a more complex array of processes, LES studies have found decreases in C in response to *N*_d_ increases, although there is often a compensating effect over the cloud lifetime (Seifert et al., 2015; Xue & Feingold, 2006), leading to a small overall $\beta_{C-\ln N_{d}}=\partial C/\partial \ln N_{d}$, suggesting a lower bound on $\beta_{C-\ln N_{d}}$ of 0.

Recent studies have applied a number of different methods to disentangle the role of meteorology from the impact of aerosols on C in observations. Three methods, based on a statistical accounting for confounders (Gryspeerdt et al., 2016), careful sampling (Christensen et al., 2017) and a neural network (Andersen et al., 2017), find rapid adjustments via C changes of between 130% and 200% of the RF of the Twomey effect. The agreement between these observational methods provides a measure of confidence in this estimate, but these methods are all based on snapshots of the aerosol‐cloud field. The inherently time‐dependent nature of cloud adjustments means that this may lead to an overestimate of the effect or an underestimate due to undetected aerosol perturbations (Possner et al., 2018) where similarly strong rapid adjustments via C were found.

### 8.3 Radiative Sensitivities and Effective Cloud Fractions

Following equation (13), the change in cloud albedo due to changes in L is given by the following:

(19) $$d\alpha_{c}=\frac{5}{6}\;\alpha_{c}\;\left(1-\alpha_{c}\right)\;d\ln L\;\Leftrightarrow \;S_{L,N}=\frac{5}{6}\;\alpha_{c}\;\left(1-\alpha_{c}\right)$$

The planetary albedo *α* can be expressed as the sum of cloudy‐sky albedo, *α*_c_, weighted by cloud fraction, C, and clear‐sky albedo, *α*_clear_, weighted by the complement:

(20) $$\alpha =C\;\alpha_{c}+(1-C)\;\alpha_{\text{clear}}=C\;\left(\alpha_{c}-\alpha_{\text{clear}}\right)+\alpha_{\text{clear}}$$

Thus, *α* scales with C with $\left(\alpha_{c}-\alpha_{\text{clear}}\right)$ as scaling factor:

(21) $$S_{C,N}=\alpha_{c}-\alpha_{\text{clear}}$$

Although *α*_c_ varies due to aerosol impacts on *N*_d_ and L, these changes are a small fraction of *α*_c_ so are ignored here. Calculating average cloud albedo across the global oceans based on CERES data for cases where the ice cloud fraction is zero, following Bender et al. (2011), yield a scaling factor of 0.3 to 0.5 for marine boundary‐layer clouds. The linear scaling is appropriate for stratocumulus clouds where clouds are capped by the inversion and therefore deepen relatively little as they widen (Feingold et al., 2017).

Like RFaci, rapid adjustments in L act on cloudy regions only, such that by analogy with equation (18), the effective cloud fraction $c_{L}$ can be written as

(22) $$c_{L}=\frac{\left\langle C_{\text{liq}}\;\alpha_{c}\;\left(1-\alpha_{c}\right)\beta_{\ln L-\ln N_{d}}\;\beta_{\ln N_{d}-\ln\tau_{a}}\;\frac{\Delta \tau_{a}}{\tau_{a}}R_{\text{SW}}^{↓}\right\rangle }{\left\langle \alpha_{c}\;\left(1-\alpha_{c}\right)\rangle \;\langle \beta_{\ln L-\ln N_{d}}\rangle \;\langle \beta_{\ln N_{d}-\ln\tau_{a}}\rangle \;\langle \frac{\Delta \tau_{a}}{\tau_{a}}\rangle \langle R_{\text{SW}}^{↓}\right\rangle }$$

Differing only through an introduction of the $\beta_{\ln L-\ln N_{d}}$ term, $c_{L}$ is very similar to *c*_N_ given by equation 18 and is calculated in a similar manner. $\beta_{\ln L-\ln N_{d}}$ is calculated using MODIS cloud retrievals at a 1°×1° resolution, with the sensitivities calculated using linear regressions on the log variables. Using CERES SSF 1deg Ed4 data (Wielicki et al., 1996) for the radiative sensitivities, gives $c_{L}$ as 0.27, an increase over $C_{liq}$ (0.22), with a similar spatial pattern (Figure 6c). The uncertainty in c_L_ depends on the retrieval uncertainty of C_liq_, and else only on the spatial pattern of the individual terms in equation (22). Using the spatial distributions of β_lnN−lnτa_ and ∆ ln τ_a_ from McCoy et al. (2017) gives a c_L_ of 0.21. Using the distributions of ∆ ln τ_a_ from Kinne (2019) gives a c_L_ of 0.29. This similarity in $c_{L}$ and *c*_N_ is supported by the resemblance of the patterns of the ERFaci[LWP] and the RFaci in observational (Gryspeerdt, Goren, et al., 2019) and modeling (Mülmenstädt et al., 2019) studies, due to the dominating influence of $C_{liq}$.

The effective cloud fraction for adjustments in C is less obvious, as it acts by changing the cloud fraction. The RFaci and the L adjustment only act by changing cloud properties, such that the area over which they act is the liquid cloud fraction. In contrast, the area over which the C adjustment can operate is any region not obscured by overlying ice cloud, leading to $\left(1-C_{\text{ice}}\right)$ as the initial cloud fraction (Gryspeerdt et al., 2016). $C_{\text{ice}}$ has to be weighted by the optical depth of the ice clouds, which determines the radiative impact of the underlying liquid clouds. This is approximated in observation‐based studies, with detected ice clouds assumed to be opaque and those below the detection limit, an optical depth of around 0.4 for MODIS (Ackerman et al., 2008), assumed transparent. The effective cloud fraction $c_{C}$ is:

(23) $$c_{C}=\frac{\left\langle \left(1-C_{\text{ice}}\right)\;\left(\alpha_{c}-\alpha_{\text{clear}}\right)\;\beta_{C-\ln N}\;\beta_{\ln N-\ln\tau_{a}}\;\frac{\Delta \tau_{a}}{\tau_{a}}\;R_{\text{SW}}^{↓}\right\rangle }{\left\langle \left(\alpha_{c}-\alpha_{\text{clear}}\right)\rangle \langle \beta_{C-\ln N}\rangle \;\langle \beta_{\ln N-\ln\tau_{a}}\rangle \;\langle \frac{\Delta \tau_{a}}{\tau_{a}}\rangle \langle R_{\text{SW}}^{↓}\right\rangle }$$

The calculation of $c_{C}$ follows $c_{L}$ (equation (22)), using MODIS cloud and AOD retrievals to calculate $\beta_{C-\ln N}$ and $\beta_{\ln N-\ln\tau_{a}}$ and CERES data for the radiative sensitivities. As for $\beta_{\ln L-\ln N}$, $\beta_{C-\ln N}$ is calculated with a linear regression within each 1°×1° gridbox. This gives $c_{C}$ as 0.59 (Figure 6d), a decrease compared to 1‐ $C_{\text{ice}}$ (0.68). The uncertainty in c_C_ depends on the retrieval uncertainty of C_ice_, and else only on the spatial pattern of the individual terms in equation (23). As with c_N_ and c_L_, the uncertainty in c_C_ is estimated using the spatial distributions of β_lnN−lnτa_ and ∆ ln τ_a_ from McCoy et al. (2017), then the distributions of ∆ ln τ_a_ from Kinne (2019). c_C_ is 1.07 using McCoy et al. (2017) and 0.76 using Kinne (2019). Note that $c_{C}$ can be greater than 1, as it is not a true cloud fraction and incorporates the covariation between the components of equation (8).

### 8.4 Summary

In summary, the contribution of rapid adjustments to globally averaged RFaci is calculated in a similar way to equation (14), as follows:

(24) $$\Delta \ln N_{d}\;\left[\beta_{\ln L-\ln N_{d}}\;S_{L,N}\;c_{L}+\beta_{\ln C-\ln N_{d}}\;S_{C,N}\;c_{C}\right]$$

For reference, Table 2 summarizes the definitions of the variables used in equation (24).

The ranges adopted for the terms of this equation are as follows:

- 0.05 to 0.17 for $\Delta \ln N_{d}$, following section 6;
−0.36 to −0.011 for $\beta_{\ln L-\ln N_{d}}$ based on the satellite analyses of Gryspeerdt et al. (2018) and Toll et al. (2017);
−54 to −56 W m^−2^ for $S_{L,N}$. This range is obtained by multiplying $S_{L,N}$ expressed in terms of planetary albedo, that is, from 0.177 to 0.184 based on propagating CERES albedo uncertainties using equation (19), by the solar constant 340 W m^−2^. The result is then multiplied by 0.9 to account for an offsetting contribution of 10% coming from the terrestrial spectrum, as calculated by GCMs (Heyn et al., 2017; Zelinka et al., 2014);
- 0.21 to 0.29 for $c_{L}$, based on satellite retrievals of cloud properties and planetary albedo;
- 0 to 0.1 for $\beta_{\ln C-\ln N_{d}}$ based on GCMs and large‐eddy simulations;
−91 to −153 W m^−2^ for $S_{C,N}$. This range is obtained by applying the method of Bender et al. (2011) to CERES data and converted to top‐of‐atmosphere radiance sensitivities using the same method as for $S_{L,N}$ above;
- 0.59 to 1.07 for $c_{C}$, based on satellite retrievals of cloud properties and planetary albedo;

Using the method described in section 2.2 to solve equation (24), rapid adjustments in L contribute from 0 to +0.56 W m^−2^ and rapid adjustments in C contribute from −1.14 to 0 W m^−2^. To obtain ERFaci, the range of −1.10 to −0.33 W m^−2^ obtained for Faci in section 6 is added to those rapid adjustments using the method described in section 2.2 to yield a range of −1.8 to −0.3 W m^−2^ for Eaci. Note that Faci and its rapid adjustments are correlated, at least in the framework of equations (15) and (24), through the term $\Delta \ln N_{d}=\beta_{\ln N-\ln a}\;\Delta \tau_{a}/\tau_{a}$.

Based on this potential correlation, an alternative way to bound ERFaci would be to directly scale rapid adjustments according to Faci. For rapid adjustments in L, Lebsock et al. (2008) and Christensen et al. (2017) find they do not completely offset RFaci, with the reduction likely less than 60% (Gryspeerdt, Goren, et al., 2019). But they may also enhance RFaci. The implementation of microphysical adjustments to aci by only one mechanism–precipitation suppression–in GCMs is suboptimal, but rarely gives an enhancement of the RFaci larger than 50% (Gryspeerdt, Mülmenstädt, et al., 2019). For rapid adjustments in C, satellite‐based studies that account for biases and confounding factors produce around a 150% enhancement to the RFaci (Andersen et al., 2017; Christensen et al., 2017; Gryspeerdt et al., 2016; Possner et al., 2018). Some high‐resolution simulations find a small change to C as a function of aerosol (Seifert et al., 2015), producing an upper bound of a 0% enhancement of the RFaci. Scaling rapid adjustments based on Faci is however only an advantage if the uncertainty in Faci is sufficiently small.

## 9 Aerosol Interactions With Ice Clouds

Ice clouds are also affected by aerosol, although the impact of aerosol depends on the aerosol type and the dominant ice nucleation mode. Sulfate aerosols facilitates homogeneous freezing of haze drops in the upper troposphere at cirrus temperatures (lower than about −38 °C). Thus, the increase in sulfate concentrations due to anthropogenic precursor emissions leads to an increase in ice crystal number, *N*_i_. This effect implies cirrus clouds with higher emissivity (less LW radiation emitted to space) and reflectivity (more SW reflected back to space), with RFs of opposite sign. Studies using satellite retrievals of *N*_i_ provide some observational evidence for an enhancement from aerosol (Gryspeerdt et al., 2018; Mitchell et al., 2018; Sourdeval et al., 2018) in regions of strong updrafts, although theoretical studies suggest that the overall magnitude of this effect is small because the primary control on the homogeneous nucleation rate is the in‐cloud updraft (DeMott et al., 1997; Jensen et al., 2013, 2016; Kay & Wood, 2008; Krämer et al., 2016; Lohmann & Kärcher, 2002).

Some aerosol types are effective heterogeneous INPs. Mineral dust (particularly feldspars; *Atkinson et al*., 2013) has been shown to be an effective INP in laboratory studies (Hoose & Möhler, 2012) and is correlated to the occurrence of glaciated clouds (Choi et al., 2010; Tan et al., 2014), so anthropogenic changes to mineral dust aerosols may change INP distributions (see section 4). The internal mixing of dust and soluble aerosol has been shown to suppress the INP activity of dust, such that anthropogenic emissions of liquid aerosol may also impact INP distributions (e.g. *Cziczo et al*., 2009). The ability of BC to act as an INP depends on its physical characteristics and mixing state, with particles containing macropores being observed to nucleate ice at cirrus temperatures (Mahrt et al., 2018), but there is increasing evidence that it is a poor INP at warmer temperatures (Kanji et al., 2017). In a situation dominated by heterogeneous nucleation, increasing INP would increase *N*_i_. In contrast, in situations dominated by homogeneous nucleation, increasing INP can reduce the available supersaturation below the homogeneous nucleation threshold, reducing *N*_i_ (Kärcher & Lohmann, 2003). Similarly, there is some evidence from satellite retrievals for a suppression of homogeneous nucleation and *N*_i_ by INP (Chylek et al., 2006; Gryspeerdt et al., 2018; Zhao et al., 2018), but the sparse nature of INP measurements makes these results uncertain. Furthermore, Christensen et al. (2014) found by studying CALIOP lidar observations of over 200 ship tracks in mixed‐phase stratocumulus clouds that increased aerosols enhance the occurrence of ice and decreased total water path in polluted clouds.

The only global estimates of RFaci[ice] and ERFaci[ice] that currently exist are produced using GCMs and mostly focus on cirrus. It is not always possible to separate the instantaneous RF from its rapid adjustments in the literature and it is not clear whether the ERFaci[ice] would scale with the RFaci in a similar fashion to the ERFaci[liq]. Gettelman et al. (2012) found a positive RFaci[ice] of +0.3 W m^−2^, or about a 20% offset of RFaci[liquid]. The importance of the fraction of particles acting as INP was highlighted by Penner et al. (2009), who found a negative RFaci[ice] of −0.3 to −0.4 W m^−2^ with a lower preindustrial INP population. Similarly, the INP efficiency of BC has a large effect on the simulated *N*_i_ and RFari (Penner et al., 2009). The uncertainty in these factors is reflected in the wide range of estimates of ERFaci[ice] (Heyn et al., 2017). In general, aerosol interactions with ice clouds are likely *N*_i_ dependent and slightly larger for those states with less homogeneous nucleation and lower ice number concentration in the base state. The uncertainty regarding the balance of homogeneous and heterogeneous nucleation for ice clouds (Gasparini & Lohmann, 2016) and the lack of observations to constrain globally cirrus INP or *N*_i_ limit how accurately the aerosol effect on ice clouds can be constrained. On balance, it seems like effects may be small and positive: by increasing ice crystal numbers, cirrus LW increases faster than SW cooling. However, as laboratory measurements have shown that BC is not as efficient an INP as previously thought and does not affect homogeneous nucleation, a large RFaci[ice] is less likely than in the past. Combined with the second order effect of aerosol on homogeneous nucleation, this suggests the resulting RFaci[ice] may be on the order of a small fraction of the total anthropogenic ERFaci, but cannot be bounded yet because of the large uncertainty in the present and preindustrial states of ice cloud nucleation pathways and INP populations.

There is no observational evidence for strong adjustments in mixed‐phase and ice clouds. Christensen et al. (2016) presents some evidence for a modest aerosol radiative warming by deep convective cores without anvil spreading, identified from CloudSat radar observations. The ability of INP to glaciate supercooled liquid clouds in the temperature range of −38 °C to 0 °C is well established theoretically and supported by observed relationships between aerosol and cloud glaciation (Choi et al., 2010; Hu et al., 2010; Kanitz et al., 2011; Tan et al., 2014) at a global scale. An increase in cloud ice through an increase in the number of INPs might be expected to increase precipitation rates (Field & Heymsfield, 2015; Lohmann, 2002), but the resulting impact on cloud water and amount along with the corresponding radiative effect of anthropogenic aerosols is currently not well constrained, with GCM studies suggesting the net overall effect to be small (Hoose et al., 2008; Lohmann, 2002). Models that include explicit treatment of INP sources and cloud microphysics at high resolution suggest a strong link between L (and reflected SW radiation) and INP driven by changes in precipitation (Vergara‐Temprado, Holden, et al., 2018).

There is some observational evidence of aerosols impacting convective clouds, with many possible mechanisms proposed (Fan et al., 2013; Rosenfeld et al., 2008; Williams et al., 2002). However, interpreting those results based on high‐resolution simulations of deep convective clouds that often last a few hours only may overemphasize the importance of microphysical perturbations that may not matter for longer climate‐relevant systems. While changes in cloud top height have proved difficult to isolate from meteorological covariations (Gryspeerdt, Stier, & Grandey, 2014), studies have found an enhancement of lightning in regions of enhanced aerosol (Yuan, Remer, Pickering, & Yu, 2011; Gryspeerdt, Stier, & Partridge, 2014; Thornton et al., 2017) and increases in cloud top height downwind of volcanoes (Yuan, Remer, & Yu, 2011; Mace & Abernathy, 2016), suggestive of an aerosol impact. The radiative effect of an aerosol impact on convective clouds is unclear. It is possible that an increase in thin anvil cirrus might act as a warming effect (Koren, Remer, et al., 2010), but there are no strong observational constraints on this process and it may be small globally due to the tendency of LW and SW effects to cancel each other (Heyn et al., 2017; Lohmann, 2008) in deep convective clouds. Local circulation changes associated with aerosol gradients could, however, be important. For example, Blossey et al. (2018) found by modeling shipping lanes that the gradient between polluted shipping lane clouds and their cleaner surroundings may strengthens updrafts in the lane.

In summary, there is clear evidence of aerosols influencing the cloud phase but uncertainties remain too large to provide robust assessments. Estimates of the ERFaci[ice] are currently sparse and the uncertainty from cloud microphysics schemes tends to rival potential aerosol effects (White et al., 2017). RFaci[ice] from cirrus would tend to be positive because of anthropogenic aerosols inducing more small ice crystals and higher ice mass through an increase in homogeneous freezing (Gettelman et al., 2012). This response might not occur depending on details of the balance of heterogeneous and homogeneous freezing (Penner et al., 2018; Zhou & Penner, 2014), and the ice nuclei population, but evidence currently supports a positive RFaci[ice] from cirrus of a few tenths of a W m^−2^. For shallow mixed‐phase clouds the effect of changes in CCN appears to be smaller than for liquid clouds (Christensen et al., 2014), but there is likely to be a positive RF in response to increases in INP, driven by increases in precipitation (Vergara‐Temprado, Miltenberger, et al., 2018).

The current lack of observational constraints on ice phase processes limits the accuracy with which the ERFaci[ice] can be constrained, so it is not bounded in this review. Observational constraints on the size of anthropogenic perturbation in *N*_i_ would allow further progress. In addition, studies providing estimates for sensitivities of ice cloud albedo, ice water path, and ice cloud fraction, as well as cloud top height, to anthropogenic aerosol changes would allow the decomposition of the ice as well as mixed‐phase and LW terms of equation (8) in a way similar to liquid clouds.

## 10 Inferences Based on Observed Changes in Temperature and Radiation

The temperature of Earth's surface has increased by 1.0 ± 0.2 °C since preindustrial times (Allen et al., 2018), and except for periods lasting less than a few decades, this increase in temperature occurred since 1850 (Hartmann et al., 2013). This increase in temperature is attributed mainly to anthropogenic forcing, primarily the ERFs due to increases in abundance of the greenhouse gases (GHG; positive, warming influence) minus the effective forcings due to increases in abundance of aerosols (negative, cooling influence) (Myhre, Shindell, et al., 2013; Bindoff et al., 2013). Here arguments are presented that the increase in global temperature together with knowledge of the GHG forcing can usefully constrain the aerosol forcing.

**Figure 7:**
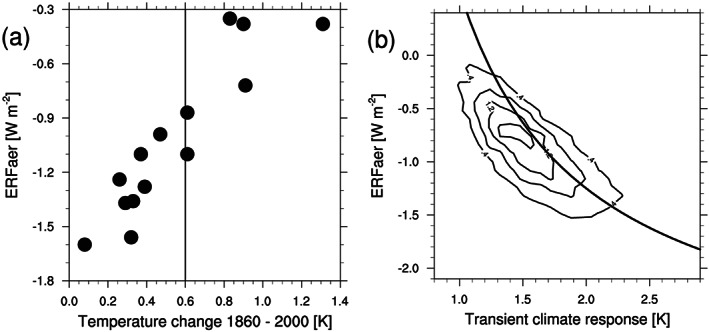
(a) Scatterplot of the change in global annual mean surface temperature between 1860 and 2000 and aerosol ERF, ERFaer, from 14 models of the CMIP5 ensemble. The vertical line at 0.6 K corresponds to the approximately observed change. After Rotstayn et al. (2015). (b) Joint histogram of the probability density function, normalized to 1, between aerosol ERF (2010 vs. 1765) and transient climate response (the global mean surface temperature increase at time of CO_2_ doubling) from a large ensemble obtained with the simple emissions‐based climate model of Smith et al. (2018). Superimposed is the relation determined from an energy balance model assuming an immediate temperature response to the total forcing. The total forcing here consists of the greenhouse gas forcing in 2011 (Myhre, Shindell, et al., 2013) 3.1 Wm^−2^, Myhre et al., 2013) plus aerosol ERF; the temperature increase in 2011 (relative to preindustrial) is taken as 1 K; and the transient sensitivity is translated to transient climate response for the ERF of doubled CO_2_ taken as 3.7 Wm^−2^.

Under the assumption that the increase in global temperature is a response to forcing, the continuous increase in Earth's temperature implies that the net average forcing has been positive throughout the period, except for short periods, for example, after volcanic eruptions (Stevens, 2015). Knowledge of greenhouse gas ERF provides a constraint on the magnitude of the total ERF: the total ERF in the year 2011 with respect to year 1750, excluding the aerosol ERF, is estimated as +3.1 ±0.4 W m^−2^ (Myhre, Shindell, et al., 2013) (uncertainty converted to ±1*σ*), implying within the stated assumption that the 2011 aerosol ERF was less negative than −3.5 W m^−2^. Rotstayn et al. (2015) analyzed the relationship between the simulated aerosol ERF (2010 vs. 1765) and the simulated change in global mean surface temperature 2000 vs. 1860 in the CMIP5 multimodel ensemble (Fig. 7a). They also cite the average temperature increase over the same period from five observational data sets at 0.6 K. The emergent constraint (Klein & Hall, 2015) constructed by Rotstayn et al. (2015) from surface temperature change suggests values of aerosol ERF around −1.0 W m^−2^.

Stevens (2015) proposed that it is possible to draw a tighter constraint on the aerosol ERF by considering an earlier part of the industrial period, when the relative importance of the aerosol ERF would be expected to have been greater due to the assumed sublinearity of the aerosol ERF. He further argued that the constraint is still tighter when assuming that increasing temperatures in the Northern Hemisphere can be linked to a net positive hemispheric ERF. The suggestion, based on a simple model for the hemispheric mean forcing, led to the conclusion that the present global aerosol ERF is unlikely to be more negative than −1.0 W m^−2^. However, slightly more comprehensive energy balance models (Booth et al., 2018) and GCMs (Kretzschmar et al., 2017) find that global mean aerosol ERFs as negative as −2 W m^−2^ are still consistent with the observed Northern Hemisphere temperature increase. It is plausible that restricting the analysis in the original study by Stevens (2015) to the northern hemispheric energy balance is hampered by the existence of and the uncertainty in the cross‐equatorial energy transports. Requiring instead that each decade during the second half of twentieth century has nonnegative total anthropogenic and natural ERF, taking into account a low efficacy of volcanic forcing (Gregory et al., 2016), shows that it is unlikely that the aerosol forcing is more negative than −1.7 W m^−2^. It is noteworthy that the GCMs in the CMIP5 ensemble with the most negative aerosol ERFs exhibit behavior that calls their fidelity into question, such as a much smaller warming than observed for many time periods of the twentieth century (Golaz et al., 2013) or unrealistic pattern in aerosol radiative effects (Stevens & Fiedler, 2017). The globally averaged emission rate of sulfate aerosols has been approximately stable since the mid‐1970s (Hoesly et al., 2018) although the geographical distribution has moved equatorward to different cloud regimes. This allows for a tighter constraint when considering only the more recent past and increasingly tight constraints may be possible in the future if aerosol ERF weakens and CO_2_ ERF increasingly dominates the overall anthropogenic ERF (Myhre et al., 2015). It is noteworthy that energy balance calculations with zero aerosol ERF can yield global mean temperature evolutions that are consistent with the instrumental record, albeit requiring low sensitivity. Schwartz (2018) showed that the observed temperature record over the period 1850 to 2011 is consistent with aerosol forcing throughout the IPCC AR5–95% uncertainty range, but requiring low transient sensitivity (1.0 K) for low‐magnitude present aerosol forcing (−0.09 W m^−2^) and high transient sensitivity (2.0 K) for high‐magnitude present aerosol forcing (−1.88 W m^−2^).

A stronger constraint could be obtained if transient climate sensitivity, the ratio of global temperature increase to global mean net forcing, were known to a good accuracy (Schwartz & Andreae, 1996; Knutti et al., 2002; Anderson, Charlson, Schwartz, et al., 2003). Figure 7b displays the hyperbolic inverse relationship between the transient climate response and aerosol ERF shown here for a large ensemble from a simple climate model and from an energy balance model. The ensemble by Smith et al. (2018) has a 16–84% confidence interval for transient climate response of 1.3 to 2.0 K, which translates into a ±1*σ* confidence interval for aerosol ERF of −1.2 to −0.6 W m^−2^. Skeie et al. (2018) obtain a quantitatively similar relationship between aerosol ERF and transient climate response using an energy balance model.

Beyond surface temperature, observations of the radiation budget may be exploited to infer clues about aerosol ERF. Murphy et al. (2009) analyze satellite retrievals of the top‐of‐atmosphere radiation budget. They find a likely range for aerosol ERF of about −0.6 to −1.5 W m^−2^. Cherian et al. (2014) explore the observations of surface solar radiation over Europe for the 1980–2005 period, in comparison to GCMs. They relate regional surface solar radiation trends simulated by the GCMs in the CMIP5 multiclimate model ensemble and simulated global mean aerosol ERF. The observed surface solar radiation trend for the 1980–2005 period over Europe, together with the GCM emergent constraint suggested a plausible range of aerosol ERF of −0.9 to −1.5 W m^−2^. In turn, Storelvmo et al. (2018) analyzed multiple surface solar radiation measurement stations across the globe with varying record lengths in comparison to the CMIP5 multimodel ensemble. They concluded that all GCMs exhibit much weaker trends in surface solar radiation than the observations they assessed, since the midtwentieth century. However, the simulated temperature trends by the GCMs are consistent with the observed temperature changes. Their result might be indicative of the possibility of a very strong aerosol ERF in the SW spectrum, but does not consider LW components.

In summary, there are two conclusions from the assessment of the climate responses: (i) the fact that surface SW radiation responded to aerosol emission changes as observed, combined with the conclusion by section 4 that anthropogenic aerosols are relatively weakly absorbing on a global average, establishes that the SW component of the aerosol ERF is negative, and (ii) different studies based on observed global temperature changes conclude that an ERF more negative than −1.2 to −2.0 W m^−2^, depending on the study, is outside the likely range considered in this assessment. On balance, −1.6 W m^−2^ is adopted here for the lower bound of the ±1*σ* confidence interval.

## 11 Synthesis and Challenges

Based on the conceptual model of aerosol instantaneous RF and rapid adjustments represented by equation 8, this review has considered several lines of evidence, including modeling and observations at various scales, on the likely strength of aerosol ERF, defined with respect to year 1850. Of all the components of aerosol ERF quantified in this review, and for which different lines of evidence support the existence of an effect, rapid adjustments to aci are most difficult to bound based on current literature, because it is challenging to properly average globally the many possible cloud responses to aerosol perturbations. The dominant uncertainties are, however, the industrial‐era changes in AOD, Δ*τ*_a_, and in cloud droplet number concentration, Δ*N*_d_, because they effectively cascade through to each ERF component under the framework of this review and its assumptions. Based on a combination of large‐scale modeling and satellite retrievals, human activities are likely to have increased AOD by 14% to 29% and cloud droplet number concentration by 5‐17% in 2005–2015 compared to the year 1850. Table 4 gives ranges in the 16–84% confidence interval for each term of equation (8). The table also lists the main lines of evidence used in this review to obtain each range. Global modeling and satellite analyses are the main lines of evidence used. Although small‐scale modeling and observation studies should in theory be the most accurate sources for the radiative sensitivities of equation (8), the current lack of a strategy for scaling their results to the global average limits their use.

**Table 4:** Ranges Obtained by This Review in the 16–84% Confidence Interval for the Variables of Equations (8), (15), and (24)

Section	Variable	Lower bound	Upper bound	Line of evidence
4	$\tau_{a}^{\text{PD}}$	0.13	0.17	Satellite retrievals
4	Δ*τ*_a_	0.02	0.04	Global modeling
4	$\Delta \ln\tau_{a}=\Delta \tau_{a}/\tau_{a}^{\text{PD}}$	0.14	0.29	Modeling/satellite
6	$\Delta \ln N_{d}=\Delta N_{d}/N_{d}$	0.05	0.17	Modeling/satellite
Aerosol‐radiation interactions				
5	$S_{\tau }^{\text{clear}}$ [W m ${}^{-2}\;\tau_{a}^{-1}$]	−27 (0.08)	−20 (0.06)	Global modeling
5	*c*_*τ*_	0.59	0.71	Global modeling
5	$S_{\tau }^{\text{cloudy}}\;c_{\tau }$ [W m^−2^]	−0.1	+0.1	Global modeling
5	*RF of ari* [W m^−2^]	−*0.37*	−*0.12*	
7	*dR*/*dR*_atm_	−0.3	−0.1	Global modeling
7	*dR*_atm_/*dτ*_a_ [W m ${}^{-2}\;\tau_{a}^{-1}$]	17	35	Global modeling
7	*RA of ari* [W m^−2^]	−*0.25*	−*0.06*	
7	*ERF of ari* [W m^−2^]	−*0.58*	−*0.23*	
Aerosol‐cloud interactions				
6	$\beta_{\ln N-\ln\tau }$	0.3	0.8	Modeling/satellite
6	*S*_*N*_ [W m^−2^]	−27 (0.079)	−26 (0.076)	Satellite retrievals
6	*c*_*N*_	0.19	0.29	Modeling/satellite
6	*RF of aci* [W m^−2^]	−*1.10*	−*0.33*	
8	$\beta_{\ln L-\ln N}$	−0.36	−0.011	Satellite analyses
8	$S_{L,N}$ [W m^−2^]	−54	−56	Mixed
8	$c_{L}$	0.21	0.29	Mixed
8	*RA of aci (liquid water path)* [W m^−2^]	*0.01*	*+0.56*	
8	$\beta_{C-\ln N}$	0	0.1	Global modeling, LES
8	$S_{C,N}$ [W m^−2^]	−91	−153	Satellite analysis
8	$c_{C}$	0.59	1.07	Mixed
8	*RA of aci (cloud fraction)* [W m^−2^]	−*1.14*	*0.0*	
8	*ERF of aci* [W m^−2^]	−*1.73*	−*0.27*	
11	**Total aerosol ERF** [W m^−2^]	−**2.19**	−**0.61**	
11	(constrained by observational inferences)	−1.60	−0.61	

*Note*. The ranges of radiative forcing (RF) and rapid adjustments (RA) components estimated from the variables are shown in italics. The bounds for total aerosol effective radiative forcing (ERF) are shown in bold. ari stands for aerosol‐radiation interactions, and aci for aerosol‐cloud interactions. Optical depths *τ*_a_ are given at 0.55 μm. Sensitivities (*S* terms) are given in W m^−2^ over the shortwave and longwave spectrum, and in parentheses also in terms of relative changes in planetary albedo for sensitivities that are predominantly acting in the shortwave spectrum. LES stands for large eddy simulation.

Evaluating equation (8) using the Monte Carlo approach described in section 2.2 yields a range for total aerosol ERF of −2.2 to −0.6 W m^−2^ at the 16% to 84% confidence level. This range is similar to that obtained from a single‐model PPE, constrained by observations, which covers −2.2 to −0.7 W m^−2^ (Regayre et al., 2018). In other words, process‐based attempts to quantify aerosol ERF do not constrain the more negative bound. As discussed in section 10, inferences based on observed climate changes provide additional constraints that narrow the distribution by making an aerosol ERF more negative than −1.6 W m^−2^ unlikely. The upper bound is not constrained further by those inferences. Consequently, the likely range of aerosol ERF obtained by this review spans −1.6 to −0.6 W m^−2^ (±1*σ* range).

This review estimates all uncertainty ranges at the 16% to 84% confidence level, as discussed in section 2.2. IPCC Assessment Reports make a different choice, reporting at the 5% to 95% confidence level. To facilitate comparisons, Table 5 translates the ranges given by this review to the 5% to 95% confidence level. However, those latter ranges are more dependent on the assumed shapes of the distributions given in Table 4, which are difficult to assess from the literature. Comparing Tables 1 and 5 suggests that working through traceable and arguable lines of evidence, as done in this review, produces uncertainty ranges that are similar to the expert judgment of IPCC AR5, albeit shifted toward more negative ERFs. Accounting for the different preindustrial reference years (1750 for IPCC AR5, 1850 for this review) would shift the present assessed range further, by 0 to −0.2 W m^−2^ (Myhre, Shindell, et al., 2013; Lund et al., 2019).

**Table 5:** Ranges Obtained by This Review for the Radiative Forcing (RF), Rapid Adjustments (RA), and Effective Radiative Forcing (ERF) of Aerosol‐Radiation Interactions (ari), Aerosol‐Cloud Interactions (aci), and Total Aerosol ERF

Variable	Lower bound	Upper bound
RFari	−0.45	−0.05
RAari	−0.35	−0.04
ERFari	−0.71	−0.14
RFaci	−1.46	−0.22
RAaci (liquid water path)	−0.06	+0.88
RAaci (cloud fraction)	−1.88	+0.16
ERFaci	−2.65	−0.07
**Total aerosol ERF**	−**3.15**	−**0.35**
(constrained by observational inferences)	−2.0	−0.35

*Note*. All values are in W m^−2^. Compared to Table 4, ranges are given as 5–95% confidence intervals.

**Figure 8:**
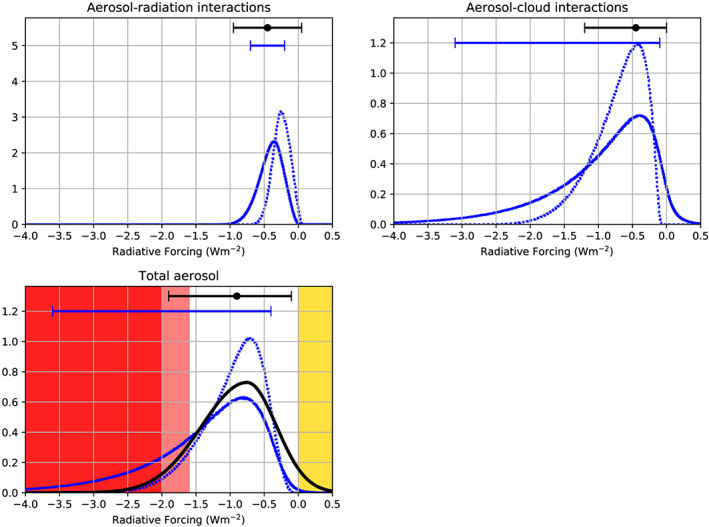
Probability distribution functions of aerosol radiative forcing (dashed lines) and effective radiative forcing (solid lines), in  W m^−2^, as derived by this review (blue) and by the Fifth Assessment Report of the IPCC (Myhre, Shindell, et al., 2013) (black). Those distributions functions are obtained based on understanding of the aerosol, cloud, and radiation physics. The top row shows distributions for (left) aerosol‐radiation interactions and (right) aerosol‐cloud interactions. The bottom row shows distributions for their sum. Corresponding 5–95% confidence intervals (90% likelihood of being in that range) for the effective radiative forcing are shown at the top of each panel, again in blue for this review and in black for the IPCC assessment. The IPCC intervals also show the best estimate as a dot. For total aerosol, colored regions indicate aerosols ERFs that are inconsistent with inferences based on observed changes in temperature (red shading for the 5–95% confidence interval, pink shading for the 17–84% confidence interval) and inconsistent with observed changes in surface radiation (yellow shading).

The full probability distribution functions for aerosol ERF and its components are shown in Figure 8. Also shown are the probability distribution functions obtained for total aerosol ERF by the IPCC Fifth Assessment Report (Myhre, Shindell, et al., 2013). The figure illustrates the reduction in the range for ERFari, which is due to a reduction of the likelihood of strong rapid adjustments to ari. The range for ERFaci is much wider in this review than in Myhre, Shindell, et al. (2013), the long tail coming from this review's wider assessment of rapid adjustments of aerosol‐cloud interactions in liquid clouds. Consequently, the range for total aerosol ERF is also much wider. This review, however, decreases the likelihood of an aerosol ERF more positive than −0.4 W m^−2^. In addition, recall that total aerosol ERF more negative than −2.0 W m^−2^ rely on more speculative aerosol‐driven cloud changes, and are not consistent with observed temperature and surface radiation changes, as discussed in section 10.

There are a number of challenges to overcome to narrow the range of aerosol ERF further. This review has already discussed the challenges associated with the imperfect knowledge of changes in aerosols over the industrial period (Carslaw et al., 2013), which in this review were encapsulated in Δ*τ*_a_ (section 4), and with aerosol interactions with ice clouds (section 9), which are not yet characterized sufficiently well to allow a global assessment of sensitivities. But other outstanding challenges should be highlighted:

- The lack of resolution of small scales by large‐scale models means that their integration of local processes into a globally averaged number is imperfect. For ari, small scales contribute significantly to spatial variability of relative humidity and unresolved aerosol amount/composition, which together determine hygroscopic growth and the amount of light scattered. Because aerosol growth factors are superlinear, application of a spatially averaged aerosol growth factor could significantly underestimate the average of the local growth factors, particularly at relative humidities above 85% (Haywood et al., 1997; Nemesure et al., 1995; Petersik et al., 2018). For aci, unresolved cloud‐scale vertical motion and turbulent mixing, and coarsely parameterized cloud and precipitation, as well as aerosol sink processes, lead to poor representations of cloud and aerosol fields, their spatiotemporal collocation, and regime‐dependent small‐scale to mesoscale interactions of processes. The emergence of global storm resolving models (Satoh et al., 2018) and the ability to perform global LES for a few days would add substantially to our ability to better quantify these processes.
- The covariability of aerosol, clouds, and meteorological conditions implies scale effects. The fidelity of a modeled ERFaci or ERFari is dependent on the ability of the model not only to generate realistic clouds on average but also to capture their covariability at smaller spatiotemporal scales. As shown by various studies, the composite response does not equal the local responses averaged up to the composite scale. In reality local data typically comprise relatively small aerosol ranges and small albedo, *N*_d_, or effective radius responses. If aci metrics or ERFs are based on aggregation of many such scenes they will tend to bias the relationships by (i) extending the range of conditions beyond the natural local fluctuations and (ii) removing the small‐scale covariability between meteorology and aerosol. The magnitude of these biases is poorly known.
- The frequency of occurrence of aerosol perturbations to planetary albedo in general, and to clouds in particular, has not yet been quantified at the global scale. For example, ship tracks are often cited as evidence that tremendous radiative effects can be generated by anthropogenic aerosol emissions, yet merely 0.002% of the world's commercial ocean‐going fleet are expected to generate a ship track in their wake at any given time (Campmany et al., 2009). So while evidence exists to support large contributions of rapid adjustments to ERFaci, these events seem infrequent. The challenge is that large‐scale attribution of changes in cloud properties to aerosol perturbations is complicated by covariability between meteorological drivers and aerosols, discussed above, and the high degree of natural variability within the cloud deck itself, which can span orders of magnitudes in cloud radiative properties (Wood et al., 2018).
- Models of all scales include a very large number of imprecisely known parameters. In such complex model systems with compensating effects of imperfectly known processes, even tight observational constraint of any model variables can leave open wide ranges of aerosol RFs (Johnson et al., 2018) so understanding why some models do well against multiple constraints is important (Penner, 2019). This model constraint limitation has become known as equifinality (Beven & Freer, 2001).
- Although this review has taken a global perspective in its assessment of aerosol ERF, geographical considerations remain important. For example, it is possible that the radiative sensitivities in equation (8) vary in time when aerosol and/or cloud patterns change in response to changes in emissions and climate. In addition, assuming that aci are saturated in the more polluted regions, then any additional anthropogenic aerosols would need to reach pristine regions in order to exert an ERFaci. More observational evidence is needed to constrain the magnitude with which anthropogenic aerosols affect pristine regions like the Southern Ocean and ocean cloud decks adjacent to continents in the eastern Pacific and eastern Atlantic Oceans.

The lack of evidence to support some of the hypotheses discussed in this review points to the need for improving scientific understanding of aerosol ERF processes and occurrences in the atmosphere. The review has identified a few critical next steps. First, scale effects are increasingly being considered among hierarchies of models and must inform global aerosol model development to ensure a more exhaustive representation of aerosol forcing and rapid adjustment mechanisms. Second, volcanic eruptions and ship tracks have provided important insights into cloud adjustments to aerosol perturbations and may provide opportunities to improve understanding of potential cloud phase shifts and ice cloud responses. Third, the strength of the constraints on aerosol ERF bounds provided by inferences based on observed climate changes is diminished by an incomplete understanding of the uncertainties affecting those methods. “Perfect model” comparisons, where top‐down methods are applied to synthetic data of known equilibrium climate sensitivity and aerosol ERF, would strengthen that important line of evidence. Fourth, statistical methods to thoroughly explore causes of model uncertainty are now being more widely adopted, and are being combined with traditional multimodel ensembles to more rigorously understand the effectiveness of observational constraints. Finally, global large‐eddy simulations hold promise to substantially improve the quantification of aerosol‐cloud interactions.

## Glossary

Aerosol: Solid and liquid particulates in suspension in the atmosphere, with the exception of cloud droplets and ice crystals.
Albedo: Ratio of reflected to incident irradiance.
Cloud condensation nuclei: Subset of the aerosol population that serves as sites where water vapor condenses to form cloud droplets.
Effective radiative forcing: The sum of radiative forcing and rapid adjustments (see those terms).
General Circulation Model: Numerical model that solves fluid mechanics equations to simulate the three‐dimensional dynamics of the moist atmosphere. Those models also include parametrizations of radiation, clouds, and, increasingly, aerosols.
Ice nucleating particle: Subset of the aerosol population that facilitate cloud ice crystal formation.
Large eddy simulation: Category of numerical models that solve the fluid dynamics equations by computing the large‐scale motion of turbulent flow.
Liquid water path: Column‐integrated cloud liquid water content, that is, mass of cloud liquid water per unit surface area.
Optical depth: Column‐integrated extinction cross section. Can be defined for any source of extinction in the atmosphere, including aerosols and clouds.
Primary aerosol: Aerosols that are emitted into the atmosphere directly as solid or liquid particulates.
Radiative forcing: Imbalance in the Earth's energy budget caused by human activities or volcanic eruptions, or changes in the output of the Sun or the orbital parameters of the Earth.
Rapid adjustments: Subset of the responses of the atmosphere‐land‐cryosphere system to radiative forcing, which happened independently of the much slower changes in sea surface temperature.
Secondary aerosol: Aerosols formed by atmospheric chemistry from gaseous precursors.
Single scattering albedo: Ratio of scattering efficiency to extinction efficiency, where extinction is the sum of scattering and absorbing. A purely scattering particle has a single‐scattering albedo of 1, and that value decreases with increasing absorption.
Twomey effect: Increase in cloud albedo caused by an increase in cloud condensation nuclei for a fixed water content. Named after the late Sean Twomey, following Twomey (1974).

## Acronyms

AeroCom: Aerosol Comparisons between Observations and Models
AERONET: AErosol RObotic NETwork
aci: Aerosol‐cloud interactions
ari: Aerosol‐radiation interactions
AOD: Aerosol optical depth
BC: Black carbon
CALIOP: Cloud‐Aerosol Lidar with Orthogonal Polarization
CCN: Cloud condensation nuclei
CERES: Clouds and the Earth's Radiant Energy System
CF: Cloud fraction
CMIP: Climate Model Intercomparison Project
ERF: Effective radiative forcing
ERFaci: Effective Radiative Forcing of Aerosol‐Cloud Interactions
ERFari: Effective Radiative Forcing of Aerosol‐Radiation Interactions
GCM: General Circulation Model
GHG: Greenhouse gas
IPCC: Intergovernmental Panel on Climate Change
IPCC AR5: 5^th^ Assessment Report of the IPCC
LES: Large eddy simulation
LWP: Liquid water path
MAC: Max Planck Institute Aerosol Climatology
MACC: Monitoring Atmospheric Composition and Climate
MODIS: Moderate Resolution Imaging Spectroradiometer
PDRMIP: Precipitation Driver Response Model Intercomparison Project
PPE: Perturbed parameter ensemble
RF: Radiative forcing
RFaci: Radiative Forcing of Aerosol‐Cloud Interactions
RFari: Radiative Forcing of Aerosol‐Radiation Interactions

## References

[rog20214-bib-0001] Ackerman, A. , Toon, O. , Taylor, J. , Johnson, D. , Hobbs, P. , & Ferek, R. (2000). Effects of aerosols on cloud albedo: Evaluation of Twomey's parameterization of cloud susceptibility using measurements of ship tracks. Journal of the Atmospheric Sciences, 57(16), 2684–2695. 10.1175/1520-0469(2000)057<2684:EOAOCA>2.0.CO;2

[rog20214-bib-0002] Ackerman, A. S. , Kirkpatrick, M. P. , Stevens, D. E. , & Toon, O. B. (2004). The impact of humidity above stratiform clouds on indirect aerosol climate forcing. Nature, 432, 1014–1017. 10.1038/nature03174 15616559

[rog20214-bib-0003] Ackerman, S. A. , Holz, R. E. , Frey, R. , Eloranta, E. W. , Maddux, B. C. , & McGill, M. (2008). Cloud detection with MODIS. Part II: Validation. Journal of Atmospheric and Oceanic Technology, 25, 1073–1086. 10.1175/2007JTECHA1053.1

[rog20214-bib-0004] Adebiyi, A. A. , Zuidema, P. , & Abel, S. J. (2015). The convolution of dynamics and moisture with the presence of shortwave absorbing aerosols over the southeast Atlantic. Journal of Climate, 28, 1997–2024. 10.1175/JCLI-D-14-00352.1

[rog20214-bib-0005] Aitken, J. (1880). On dust, fogs, and clouds. Proceedings of the Royal Society of Edinburgh, 11(108), 122–126.

[rog20214-bib-0006] Albrecht, B. A. (1989). Aerosols, cloud microphysics, and fractional cloudiness. Science, 245(4923), 1227–1230. 10.1126/science.245.4923.1227 17747885

[rog20214-bib-0007] Allen, M. R. , O. P. Dube , W. Solecki , F. Aragón‐Durand , W. Cramer , S. Humphreys , M. Kainuma , J. Kala , N. Mahowald , Y. Mulugetta , R. Perez , M. Wairiu , and K. Zickfeld (2018), Framing and context, in Global warming of 1.5^∘^C, edited by Masson‐Delmotte V., Zhai P., Pörtner H. O., Roberts D., Skea J., Shukla P., Pirani A., Moufouma‐Okia W., Péan C., Pidcock R., Connors S., Matthews J. B. R., Chen Y., Zhou X., Gomis M. I., Lonnoy E., Maycock T., Tignor M., and Waterfield T., chap. 1, Cambridge University Press, Cambridge, United Kingdom and New York, NY, USA.

[rog20214-bib-0008] Alterskjær, K. , Kristjánsson, J. E. , & Seland, Ø. (2012). Sensitivity to deliberate sea salt seeding of marine clouds—Observations and model simulations. Atmospheric Chemistry and Physics, 12, 2795–2807. 10.5194/acp-12-2795-2012

[rog20214-bib-0009] Amiri‐Farahani, A. , Allen, R. J. , Neubauer, D. , & Lohmann, U. (2017). Impact of saharan dust on north atlantic marine stratocumulus clouds: Importance of the semidirect effect. Atmospheric Chemistry and Physics, 17, 6305–6322. 10.5194/acp-17-6305-2017

[rog20214-bib-0010] Andersen, H. , Cermak, J. , Fuchs, J. , Knutti, R. , & Lohmann, U. (2017). Understanding the drivers of marine liquid‐water cloud occurrence and properties with global observations using neural networks. Atmospheric Chemistry and Physics, 17(15), 9535–9546. 10.5194/acp-17-9535-2017

[rog20214-bib-0011] Anderson, T. L. , Charlson, R. J. , Schwartz, S. E. , Knutti, R. , Boucher, O. , Rodhe, H. , & Heintzenberg, J. (2003). Climate forcing by aerosols—A hazy picture. Science, 300, 1103–1104. 10.1126/science.1084777 12750507

[rog20214-bib-0012] Anderson, T. L. , Charlson, R. J. , Winker, D. M. , Ogren, J. A. , & Holmen, K. (2003). Mesoscale variations of tropospheric aerosols. Journal of the Atmospheric Sciences, 60(1), 119–136. 10.1175/1520-0469(2003)060<0119:MVOTA>2.0.CO;2

[rog20214-bib-0013] Andrews, E. , Ogren, J. A. , Kinne, S. , & Samset, B. (2017). Comparison of aod, aaod and column single scattering albedo from aeronet retrievals and in situ profiling measurements. Atmospheric Chemistry and Physics, 17(9), 6041–6072. 10.5194/acp-17-6041-2017

[rog20214-bib-0014] Andrews, T. , Forster, P. M. , Boucher, O. , Bellouin, N. , & Jones, A. (2010). Precipitation, radiative forcing and global temperature change. Geophysical Research Letters, 37, l14701 10.1029/2010GL043991

[rog20214-bib-0015] Barahona, D. , Molod, A. , Bacmeister, J. , Nenes, A. , Gettelman, A. , Morrison, H. , Phillips, V. , & Eichmann, A. (2014). Development of two‐moment cloud microphysics for liquid and ice within the NASA Goddard Earth Observing System Model (GEOS‐5). Geoscientific Model Development, 7, 1733–1766. 10.5194/gmd-7-1733-2014

[rog20214-bib-0016] Bellouin, N. , Mann, G. W. , Woodhouse, M. T. , Johnson, C. , Carslaw, K. S. , & Dalvi, M. (2013). Impact of the modal aerosol scheme GLOMAP‐mode on aerosol forcing in the Hadley Centre Global Environmental Model. Atmospheric Chemistry and Physics, 13, 3027–3044. 10.5194/acp-13-3027-2013

[rog20214-bib-0017] Bellouin, N. , Quaas, J. , Morcrette, J.‐J. , & Boucher, O. (2013). Estimates of aerosol radiative forcing from the MACC re‐analysis. Atmospheric Chemistry and Physics, 13(4), 2045–2062. 10.5194/acp-13-2045-2013

[rog20214-bib-0018] Bender, F. , Charlson, R. , Ekman, A. , & Leahy, L. (2011). Quantification of monthly mean regional‐scale albedo of marine stratiform clouds in satellite observations and GCMs. Journal of Applied Meteorology and Climatology, 50, 2139–2148. 10.1175/JAMC-D-11-049.1

[rog20214-bib-0019] Benedetti, A. , Morcrette, J.‐J. , Boucher, O. , Dethof, A. , Engelen, R. J. , Fisher, M. , Flentje, H. , Huneeus, N. , Jones, L. , Kaiser, J. W. , Kinne, S. , Mangold, A. , Razinger, M. , Simmons, A. J. , & Suttie, M. (2009). Aerosol analysis and forecast in the European Centre for Medium‐Range Weather Forecasts Integrated Forecast System: 2. data assimilation. Journal of Geophysical Research: Atmospheres, 114, D13,205 10.1029/2008JD011115

[rog20214-bib-0020] Beven, K. , & Freer, J. (2001). Equifinality, data assimilation, and uncertainty estimation in mechanistic modelling of complex environmental systems using the glue methodology. Journal of Hydrology, 249(1), 11–29. 10.1016/S0022-1694(01)00421-8

[rog20214-bib-0021] Bindoff, N. , P. Stott , K. AchutaRao , M. Allen , N. Gillett , D. Gutzler , K. Hansingo , G. Hegerl , Y. Hu , S. Jain , I. Mokhov , J. Overland , J. Perlwitz , R. Sebbari , and X. Zhang (2013). Detection and attribution of climate change: From global to regional In Stocker T., Qin D., Plattner G.‐K., Tignor M., Allen S., Boschung J., Nauels A., Xia Y., Bex V., and Midgley P. (Eds.). Climate Change 2013: The Physical Science Basis. Contribution of Working Group I to the Fifth Assessment Report of the Intergovernmental Panel on Climate Change (Chap. 10, pp. 867–952). Cambridge, United Kingdom and New York, NY, USA: Cambridge University Press 10.1017/CBO9781107415324.022.

[rog20214-bib-0022] Bloch, M. R. (1965). A hypothesis for the change of ocean levels depending on the albedo of the polar ice caps. Palaeogeogr. Palaeocl., 1, 127–142. 10.1016/0031-0182(65)90010-6

[rog20214-bib-0023] Blossey, P. N. , Bretherton, C. S. , Thornton, J. A. , & Virts, K. S. (2018). Locally enhanced aerosols over a shipping lane produce convective invigoration but weak overall indirect effects in cloud‐resolving simulations. Geophysical Research Letters, 45(17), 9305–9313. 10.1029/2018GL078682

[rog20214-bib-0024] Bogenschutz, P. A. , Gettelman, A. , Morrison, H. , Larson, V. E. , Craig, C. , & Schanen, D. P. (2013). Higher‐order turbulence closure and its impact on climate simulations in the Community Atmosphere Model. Journal of Climate, 26(23), 9655–9676. 10.1175/JCLI-D-13-00075.1

[rog20214-bib-1001] Bollasina, M. A. , Ming, Y. , & Ramaswamy, V. (2011). Anthropogenic aerosols and the weakening of the South Asian summer monsoon. Science, 334, 502–505. 10.1126/science.1204994 21960529

[rog20214-bib-0025] Bond, T. C. , Doherty, S. J. , Fahey, D. W. , Forster, P. M. , Berntsen, T. , DeAngelo, B. J. , Flanner, M. G. , Ghan, S. , Kächer, B. , Koch, D. , Kinne, S. , Kondo, Y. , Quinn, P. K. , Sarofim, M. C. , Schultz, M. G. , Schulz, M. , Venkataraman, C. , Zhang, H. , Zhang, S. , Bellouin, N. , Guttikunda, S. K. , Hopke, P. K. , Jacobson, M. Z. , Kaiser, J. W. , Klimont, Z. , Lohmann, U. , Schwarz, J. P. , Shindell, D. , Storelvmo, T. , Warren, S. G. , & Zender, C. S. (2013). Bounding the role of black carbon in the climate system: A scientific assessment. Journal of Geophysical Research: Atmospheres, 118(11), 5380–5552. 10.1002/jgrd.50171

[rog20214-bib-0026] Booth, B. , Harris, G. , Jones, A. , Wilcox, L. , Hawcroft, M. , & Carslaw, K. (2018). Comments on “rethinking the lower bound on aerosol radiative forcing”. Journal of Climate, 31(22), 9407–9412. 10.1175/JCLI-D-17-0369.1

[rog20214-bib-0027] Boucher, O. , & Haywood, J. (2001). On summing the components of radiative forcing of climate change. Climate Dynamics, 18(3‐4), 297–302. 10.1007/s003820100185

[rog20214-bib-0028] Boucher, O. , & Lohmann, U. (1995). The sulfate‐ccn‐cloud albedo effect. Tellus B, 47(3), 281–300. 10.1034/j.1600-0889.47.issue3.1.x

[rog20214-bib-0029] Boucher, O. , & Quaas, J. (2013). Water vapour affects both rain and aerosol optical depth. Nature Geoscience, 6(1), 4–5. 10.1038/ngeo1692

[rog20214-bib-0030] Boucher, O. , D. Randall , P. Artaxo , C. Bretherton , G. Feingold , P. Forster , V.‐M. Kerminen , Y. Kondo , H. Liao , U. Lohmann , P. Rasch , S. Satheesh , S. Sherwood , B. Stevens , and X. Zhang (2013). Clouds and aerosols, in Climate change 2013: The physical science basis. Contribution of Working Group I to the Fifth Assessment Report of the Intergovernmental Panel on Climate Change, edited by Stocker T., Qin D., Plattner G.‐K., Tignor M., Allen S., Boschung J., Nauels A., Xia Y., Bex V., and Midgley P., chap. 7, pp. 571–658, Cambridge University Press, Cambridge, United Kingdom and New York, NY, USA, 10.1017/CBO9781107415324.016.

[rog20214-bib-0031] Boucher, O. , Schwartz, S. E. , Ackerman, T. P. , Anderson, T. L. , Bergstrom, B. , Bonnel, B. , Chylek, P. , Dahlback, A. , Fouquart, Y. , Fu, Q. , Halthore, R. N. , Haywood, J. M. , Iversen, T. , Kato, S. , Kinne, S. , Kirkevåg, A. , Knapp, K. R. , Lacis, A. , Laszlo, I. , Mishchenko, M. I. , Nemesure, S. , Ramaswamy, V. , Roberts, D. L. , Russell, P. , Schlesinger, M. E. , Stephens, G. L. , Wagener, R. , Wang, M. , Wong, J. , & Yang, F. (1998). Intercomparison of models representing direct shortwave radiative forcing by sulfate aerosols. Journal of Geophysical Research, 103(D14), 16,979–16,998. 10.1029/98JD00997

[rog20214-bib-0032] Brenguier, J.‐L. , Pawlowska, H. , & Schüller, L. (2003). Cloud microphysical and radiative properties for parameterization and satellite monitoring of the indirect effect of aerosol on climate. Journal of Geophysical Research: Atmospheres, 108(D15), 8632 10.1029/2002JD002682

[rog20214-bib-1002] Brenguier, J.‐L. , Pawlowska, H. , Schuller, L. , Preusker, R. , Fischer, J. , & Fouquart, Y. (2000). Radiative properties of boundary layer clouds: Droplet effective radius versus number concentration. Journal of the Atmospheric Sciences, 57, 803–821.

[rog20214-bib-0033] Brennan, J. , Kaufman, Y. , Koren, I. , & Rong Rong, L. (2005). Aerosol‐cloud interaction‐misclassification of MODIS clouds in heavy aerosol. IEEE T. Geosci. Remote, 43, 911–915. 10.1109/TGRS.2005.844662

[rog20214-bib-0034] Bretherton, C. S. , Blossey, P. N. , & Uchida, J. (2007). Cloud droplet sedimentation, entrainment efficiency, and subtropical stratocumulus albedo. Geophysical Research Letters, 34, L03,813 10.1029/2006GL027648

[rog20214-bib-0035] Campmany, E. , Grainger, R. G. , & Dean, S. M. (2009). Automatic detection of ship tracks in atsr‐2 satellite imagery. Atmospheric Chemistry and Physics, 9(6), 1899–1905. 10.5194/acp-9-1899-2009

[rog20214-bib-0036] Carslaw, K. S. , Gordon, H. , Hamilton, D. S. , Johnson, J. S. , Regayre, L. A. , Yoshioka, M. , & Pringle, K. J. (2017). Aerosols in the pre‐industrial atmosphere. Current Climate Change Reports, 3(1), 1–15. 10.1007/s40641-017-0061-2 32226722 PMC7089647

[rog20214-bib-0037] Carslaw, K. S. , Lee, L. A. , Reddington, C. L. , Pringle, K. J. , Rap, A. , Forster, P. M. , Mann, G. W. , Spracklen, D. V. , Woodhouse, M. T. , Regayre, L. A. , & Pierce, J. R. (2013). Large contribution of natural aerosols to uncertainty in indirect forcing. Nature, 503(7474), 67–71. 10.1038/nature12674 24201280

[rog20214-bib-0038] Chand, D. , Wood, R. , Anderson, T. L. , Satheesh, S. K. , & Charlson, R. J. (2009). Satellite‐derived direct radiative effect of aerosols dependent on cloud cover. Nature Geoscience, 2(3), 181–184. 10.1038/ngeo437

[rog20214-bib-0039] Chand, D. , Wood, R. , Ghan, S. J. , Wang, M. , Ovchinnikov, M. , Rasch, P. J. , Miller, S. , Schichtel, B. , & Moore, T. (2012). Aerosol optical depth increase in partly cloudy conditions. Journal of Geophysical Research, 117(D17), 17,207 10.1029/2012JD017894

[rog20214-bib-0040] Charlson, R. J. , Schwartz, S. E. , Hales, J. M. , Cess, R. D. , Coakley, J. A. , Hansen, J. E. , & Hofmann, D. J. (1992). Climate forcing by anthropogenic aerosols. Science, 255(5043), 423–430. 10.1126/science.255.5043.423 17842894

[rog20214-bib-0041] Chen, Y.‐C. , Christensen, M. W. , Stephens, G. L. , & Seinfeld, J. H. (2014). Satellite‐based estimate of global aerosol‐cloud radiative forcing by marine warm clouds. Nature Geoscience, 7(9), 643–646. 10.1038/NGEO2214

[rog20214-bib-0042] Cherian, R. , Quaas, J. , Salzmann, M. , & Wild, M. (2014). Pollution trends over Europe constrain global aerosol forcing as simulated by climate models. Geophysical Research Letters, 41, 2176–2181. 10.1002/2013GL058715

[rog20214-bib-0043] Choi, Y.‐S. , Lindzen, R. S. , Ho, C.‐H. , & Kim, J. (2010). Space observations of cold‐cloud phase change. Proceedings of the National Academy of Sciences of the United States of America, 107(25), 11,211–11,216. 10.1073/pnas.1006241107 20534562 PMC2895094

[rog20214-bib-0044] Christensen, M. W. , Chen, Y.‐C. , & Stephens, G. L. (2016). Aerosol indirect effect dictated by liquid clouds. Journal of Geophysical Research: Atmospheres, 121(24), 14,636–14,650. 10.1002/2016JD025245

[rog20214-bib-0045] Christensen, M. W. , Neubauer, D. , Poulsen, C. A. , Thomas, G. E. , McGarragh, G. R. , Povey, A. C. , Proud, S. R. , & Grainger, R. G. (2017). Unveiling aerosol‐cloud interactions—Part 1: Cloud contamination in satellite products enhances the aerosol indirect forcing estimate. Atmospheric Chemistry and Physics, 17(21), 13,151–13,164. 10.5194/acp-17-13151-2017

[rog20214-bib-0046] Christensen, M. W. , & Stephens, G. L. (2011). Microphysical and macrophysical responses of marine stratocumulus polluted by underlying ships: Evidence of cloud deepening. Journal of Geophysical Research, 116(D3), D03201 10.1029/2010JD014638

[rog20214-bib-0047] Christensen, M. W. , Suzuki, K. , Zambri, B. , & Stephens, G. L. (2014). Ship track observations of a reduced shortwave aerosol indirect effect in mixed‐phase clouds. Geophysical Research Letters, 41, 6970–6977. 10.1002/2014GL061320

[rog20214-bib-1003] Chung, E.‐S. , & Soden, B. J. (2017). Hemispheric climate shifts driven by anthropogenic aerosol‐cloud interactions. Nature Geoscience, 10, 566–571. 10.1038/ngeo2988

[rog20214-bib-0048] Chýlek, P. , & Coakley, J. A. (1974). Aerosols and climate. Science, 183(4120), 75–77. 10.1126/science.183.4120.75 17743150

[rog20214-bib-0049] Chylek, P. , Dubey, M. K. , Lohmann, U. , Ramanathan, V. , Kaufman, Y. J. , Lesins, G. , Hudson, J. , Altmann, G. , & Olsen, S. (2006). Aerosol indirect effect over the indian ocean. Geophysical Research Letters, 33, L06806 10.1029/2005GL025397

[rog20214-bib-0050] Conover, J. H. (1966). Anomalous cloud lines. Journal of the Atmospheric Sciences, 23(6), 778–785. 10.1175/1520-0469(1966)023<0778:ACL>2.0.CO;2

[rog20214-bib-0051] Coopman, Q. , Riedi, J. , Finch, D. P. , & Garrett, T. J. (2018). Evidence for changes in arctic cloud phase due to long‐range pollution transport. Geophysical Research Letters, 45(19), 10,709–10,718. 10.1029/2018GL079873

[rog20214-bib-0052] Costantino, L. (2012). Analysis of aerosol‐cloud interaction from space. (PhD thesis). Université de Versailles et Saint‐Quentin‐en‐Yvelines Retrieved from https://hal.archives-ouvertes.fr/tel-01430153/file/Costantino_Aerosol-Cloud_Interaction_from_Space.pdf

[rog20214-bib-0082] de Graaf, M. , Bellouin, N. , Tilstra, L. G. , Haywood, J. , & Stammes, P. (2014). Aerosol direct radiative effect of smoke over clouds over the southeast atlantic ocean from 2006 to 2009. Geophysical Research Letters, 41, 7723–7730. 10.1002/2014GL061103

[rog20214-bib-0053] DeMott, P. J. , Rogers, D. C. , & Kreidenweis, S. M. (1997). The susceptibility of ice formation in upper tropospheric clouds to insoluble aerosol components. Journal of Geophysical Research, 102(D16), 19,575–19,584. 10.1029/97JD01138

[rog20214-bib-0054] Dey, S. , Di, G. L. , Zhao, G. , Jones, A. L. , & McFarquhar, G. M. (2011). Satellite‐observed relationships between aerosol and trade‐wind cumulus cloud properties over the Indian Ocean. Geophysical Research Letters, 38, 01804 10.1029/2010GL045588

[rog20214-bib-0055] Ding, X. , M. Zheng , L. Yu , X. Zhang , R. J. Weber , B. Yan , A. G. Russell , E. S. Edgerton , and X. Wang (2008), Spatial and seasonal trends in biogenic secondary organic aerosol tracers and water‐soluble organic carbon in the southeastern united states, Environmental Science & Technology, 42(14), 5171–5176, doi:10.1021/es7032636, pMID: 18754365. 18754365

[rog20214-bib-0056] Eck, T. , Holben, B. N. , Reid, J. S. , Dubovik, O. , Smirnov, A. , O'Neill, N. T. , Slutsker, I. , & Kinne, S. (1999). Wavelength dependence of the optical depth of biomass burning, urban, and desert dust aerosols. Journal of Geophysical Research: Atmospheres, 104(D24), 31,333–31,349. 10.1029/1999JD900923

[rog20214-bib-0057] Evan, A. T. , Flamant, C. , Fiedler, S. , & Doherty, O. (2014). An analysis of aeolian dust in climate models. Geophysical Research Letters, 41, 5996–6001. 10.1002/GL060545

[rog20214-bib-1004] Eyring, V. , Bony, S. , Meehl, G. A. , Senior, C. A. , Stevens, B. , Stouffer, R. J. , & Taylor, K. E. (2016). Overview of the Coupled Model Intercomparison Project Phase 6 (CMIP6) experimental design and organization. Geoscientific Model Development, 9(5), 1937–1958. 10.5194/gmd-9-1937-2016

[rog20214-bib-0058] Fan, J. , Leung, L. R. , Rosenfeld, D. , Chen, Q. , Li, Z. , Zhang, J. , & Yan, H. (2013). Microphysical effects determine macrophysical response for aerosol impacts on deep convective clouds. Proceedings of the National Academy of Sciences of the United States of America, 110(48), E4581–E4590. 10.1073/pnas.1316830110 24218569 PMC3845117

[rog20214-bib-0059] Feingold, G. , Balsells, J. , Glassmeier, F. , Yamaguchi, T. , Kazil, J. , & McComiskey, A. (2017). Analysis of albedo versus cloud fraction relationships in liquid water clouds using heuristic models and large eddy simulation. Journal of Geophysical Research: Atmospheres, 122(13), 7086–7102. 10.1002/2017JD026467

[rog20214-bib-0060] Feingold, G. , Eberhard, W. L. , Veron, D. E. , & Previdi, M. (2003). First measurements of the Twomey indirect effect using ground‐based remote sensors. Geophysical Research Letters, 30(6), 1287 10.1029/2002GL016633

[rog20214-bib-0061] Feingold, G. , Jiang, H. , & Harrington, J. Y. (2005). On smoke suppression of clouds in Amazonia. Geophysical Research Letters, 32, L02804 10.1029/2004GL021369

[rog20214-bib-0062] Feingold, G. , Koren, I. , Yamaguchi, T. , & Kazil, J. (2015). On the reversibility of transitions between closed and open cellular convection. Atmospheric Chemistry and Physics, 15(13), 7351–7367. 10.5194/acp-15-7351-2015

[rog20214-bib-1005] Feingold, G. , Kreidenweis, S. M. , Stevens, B. , & Cotton, W. R. (1996). Numerical simulations of stratocumulus processing of cloud condensation nuclei through collision‐coalescence. Journal of Geophysical Research, 101(D16), 21,391–21,402. 10.1029/96JD01552

[rog20214-bib-0063] Fiedler, S. , Kinne, S. , Huang, W. T. K. , Räisänen, P. , O'Donnell, D. , Bellouin, N. , Stier, P. , Merikanto, J. , van Noije, T. , Makkonen, R. , & Lohmann, U. (2019). Anthropogenic aerosol forcing—Insights from multiple estimates from aerosol‐climate models with reduced complexity. Atmospheric Chemistry and Physics, 19(10), 6821–6841. 10.5194/acp-19-6821-2019

[rog20214-bib-0064] Fiedler, S. , Knippertz, P. , Woodward, S. , Martin, G. M. , Bellouin, N. , Ross, A. N. , Heinold, B. , Schepanski, K. , Birch, C. E. , & Tegen, I. (2016). A process‐based evaluation of dust‐emitting winds in the CMIP5 simulation of HadGEM2‐ES. Climate Dynamics, 46(3‐4), 1107–1130. 10.1007/s00382-015-2635-9

[rog20214-bib-0065] Field, P. R. , & Heymsfield, A. J. (2015). Importance of snow to global precipitation. Geophysical Research Letters, 42, 9512–9520. 10.1002/2015GL065497

[rog20214-bib-0066] Flanner, M. G. , Zender, C. S. , Randerson, J. T. , & Rasch, P. J. (2007). Present‐day climate forcing and response from black carbon in snow. Journal of Geophysical Research, 112(D11), D11202 10.1029/2006JD008003

[rog20214-bib-0067] Forster, P. (2016). Inference of climate sensitivity from analysis of Earth's energy budget. Annual Review of Earth and Planetary Sciences, 44(1), 85–106. 10.1146/annurev-earth-060614-105156

[rog20214-bib-0068] Forster, P. , V. Ramaswamy , P. Artaxo , T. Berntsen , R. Betts , D. Fahey , J. Haywood , J. Lean , D. Lowe , G. Myhre , J. Nganga , R. Prinn , G. Raga , M. Schulz , and R. V. Dorland (2007), Clouds and aerosols, in Climate change 2007: The physical science basis. Contribution of Working Group I to the Fourth Assessment Report of the Intergovernmental Panel on Climate Change, edited by Solomon S., Qin D., Manning M., Chen Z., Marquis M., Averyt K., Tignor M., and Miller H., chap. 2, pp. 130–234, Cambridge University Press, Cambridge, United Kingdom and New York, NY, USA.

[rog20214-bib-0069] Garrett, T. J. , L. M. Russell , V. Ramaswamy , S. F. Maria , and B. J. Huebert (2003), Microphysical and radiative evolution of aerosol plumes over the tropical North Atlantic Ocean, Journal of Geophysical Research: Atmospheres, 108(D1), AAC 11–1–AAC 11–16, doi:10.1029/2002JD002228, 4022.

[rog20214-bib-0070] Garrett, T. J. , Zhao, C. , Dong, X. , Mace, G. G. , & Hobbs, P. V. (2004). Effects of varying aerosol regimes on low‐level arctic stratus. Geophysical Research Letters, 31, n/a 10.1029/2004GL019928

[rog20214-bib-0071] Gasparini, B. , & Lohmann, U. (2016). Why cirrus cloud seeding cannot substantially cool the planet. Journal of Geophysical Research, 121(9), 4877–4893. 10.1002/2015JD024666

[rog20214-bib-0072] Gassó, S. (2008). Satellite observations of the impact of weak volcanic activity on marine clouds. Journal of Geophysical Research: Atmospheres, 113(D14), D14S19 10.1029/2007JD009106

[rog20214-bib-0073] Gettelman, A. (2015). Putting the clouds back in aerosol‐cloud interactions. Atmospheric Chemistry and Physics, 15(21), 12,397–12,411. 10.5194/acp-15-12397-2015

[rog20214-bib-0074] Gettelman, A. , Liu, X. , Barahona, D. , Lohmann, U. , & Chen, C. (2012). Climate impacts of ice nucleation. Journal of Geophysical Research, 117(D20). 10.1029/2012JD017950

[rog20214-bib-0075] Gettelman, A. , & Morrison, H. (2015). Advanced two‐moment bulk microphysics for global models. Part I: Off‐line tests and comparison with other schemes. Journal of Climate, 28(3), 1268–1287. 10.1175/JCLI-D-14-00102.1

[rog20214-bib-1006] Ghan, S. J. , & Schwartz, S. E. (2007). Aerosol properties and processes: A path from field and laboratory measurements to global climate models. Bulletin of the American Meteorological Society, 88, 1059–1083. 10.1175/BAMS-88-7-1059

[rog20214-bib-0076] Ghan, S. , Wang, M. , Zhang, S. , Ferrachat, S. , Gettelman, A. , Griesfeller, J. , Kipling, Z. , Lohmann, U. , Morrison, H. , Neubauer, D. , Partridge, D. G. , Stier, P. , Takemura, T. , Wang, H. , & Zhang, K. (2016). Challenges in constraining anthropogenic aerosol effects on cloud radiative forcing using present‐day spatiotemporal variability. P. Natl. Acad. Sci. USA, 113(21), 5804–5811. 10.1073/pnas.1514036113 PMC4889346 26921324

[rog20214-bib-0077] Ginoux, P. , Garbuzov, D. , & Hsu, N. C. (2010). Identification of anthropogenic and natural dust sources using Moderate Resolution Imaging Spectroradiometer (MODIS) Deep Blue level 2 data. Journal of Geophysical Research: Atmospheres, 115(D5), D05204 10.1029/2009JD012398

[rog20214-bib-0078] Ginoux, P. , Prospero, J. M. , Gill, T. E. , Hsu, N. C. , & Zhao, M. (2012). Global‐scale attribution of anthropogenic and natural dust sources and their emission rates based on MODIS Deep Blue aerosol products. Reviews of Geophysics, 50 10.1029/2012RG000388

[rog20214-bib-0079] Girard, E. , Blanchet, J.‐P. , Dubois , & Y. (2004). Effects of arctic sulphuric acid aerosols on wintertime low‐level atmospheric ice crystals, humidity and temperature at alert, nunavut. Atmospheric Research, 73, 131–148.

[rog20214-bib-0080] Golaz, J. , Horowitz, L. W. , & Levy, H. (2013). Cloud tuning in a coupled climate model: Impact on 20th century warming. Geophysical Research Letters, 40, 2246–2251. 10.1002/grl.50232

[rog20214-bib-0081] Goren, T. , & Rosenfeld, D. (2012). Satellite observations of ship emission induced transitions from broken to closed cell marine stratocumulus over large areas. Journal of Geophysical Research: Atmospheres, 117(D17), n/a 10.1029/2012JD017981

[rog20214-bib-0083] Grandey, B. S. , Stier, P. , & Wagner, T. M. (2013). Investigating relationships between aerosol optical depth and cloud fraction using satellite, aerosol reanalysis and general circulation model data. Atmospheric Chemistry and Physics, 13(6), 3177–3184. 10.5194/acp-13-3177-2013

[rog20214-bib-0084] Grassl, H. (1975). Albedo reduction and radiative heating of clouds by absorbing aerosol particles. Beiträge zur Physik der Atmosphäre, 48, 199–210.

[rog20214-bib-0085] Gregory, J. M. , Andrews, T. , Good, P. , Mauritsen, T. , & Forster, P. M. (2016). Small global‐mean cooling due to volcanic radiative forcing. Climate Dynamics, 47(12), 3979–3991. 10.1007/s00382-016-3055-1

[rog20214-bib-0086] Grosvenor, D. P. , Sourdeval, O. , Zuidema, P. , Ackerman, A. , Alexandrov, M. D. , Bennartz, R. , Boers, R. , Cairns, B. , Chiu, J. C. , Christensen, M. , Deneke, H. , Diamond, M. , Feingold, G. , Fridlind, A. , Hünerbein, A. , Knist, C. , Kollias, P. , Marshak, A. , McCoy, D. , Merk, D. , Painemal, D. , Rausch, J. , Rosenfeld, D. , Russchenberg, H. , Seifert, P. , Sinclair, K. , Stier, P. , van Diedenhoven, B. , Wendisch, M. , Werner, F. , Wood, R. , Zhang, Z. , & Quaas, J. (2018). Remote sensing of droplet number concentration in warm clouds: A review of the current state of knowledge and perspectives. Reviews of Geophysics, 56(2), 409–453. 10.1029/2017RG000593 30148283 PMC6099364

[rog20214-bib-0087] Gryspeerdt, E. , Goren, T. , Sourdeval, O. , Quaas, J. , Mülmenstädt, J. , Dipu, S. , Unglaub, C. , Gettelman, A. , & Christensen, M. (2019). Constraining the aerosol influence on cloud liquid water path. Atmospheric Chemistry and Physics, 19(8), 5331–5347. 10.5194/acp-19-5331-2019

[rog20214-bib-0088] Gryspeerdt, E. , Mülmenstädt, J. , Gettelman, A. , Malavelle, F. F. , Morrison, H. , Neubauer, D. , Partridge, D. G. , Stier, P. , Takemura, T. , Wang, H. , Wang, M. , & Zhang, K. (2019). Surprising similarities in model and observational aerosol radiative forcing estimates. Atmospheric Chemistry and Physics Discussions, 2019, 1–18. 10.5194/acp-2019-533 PMC7668122 33204244

[rog20214-bib-0089] Gryspeerdt, E. , Quaas, J. , & Bellouin, N. (2016). Constraining the aerosol influence on cloud fraction. Journal of Geophysical Research, 121(7), 3566–3583. 10.1002/2015JD023744

[rog20214-bib-0090] Gryspeerdt, E. , Quaas, J. , Ferrachat, S. , Gettelman, A. , Ghan, S. , Lohmann, U. , Morrison, H. , Neubauer, D. , Partridge, D. G. , Stier, P. , Takemura, T. , Wang, H. , Wang, M. , & Zhang, K. (2017). Constraining the instantaneous aerosol influence on cloud albedo. Proceedings of the National Academy of Sciences of the United States of America, 114(19), 4899–4904. 10.1073/pnas.1617765114 28446614 PMC5441736

[rog20214-bib-0091] Gryspeerdt, E. , Sourdeval, O. , Quaas, J. , Delanoë, J. , Krämer, M. , & Kühne, P. (2018). Ice crystal number concentration estimates from lidar‐radar satellite remote sensing—Part 2: Controls on the ice crystal number concentration. Atmospheric Chemistry and Physics, 18(19), 14,351–14,370. 10.5194/acp-18-14351-2018

[rog20214-bib-0092] Gryspeerdt, E. , & Stier, P. (2012). Regime‐based analysis of aerosol‐cloud interactions. Geophysical Research Letters, 39, 21802 10.1029/2012GL053221

[rog20214-bib-0093] Gryspeerdt, E. , Stier, P. , & Grandey, B. S. (2014). Cloud fraction mediates the aerosol optical depth‐cloud top height relationship. Geophysical Research Letters, 41, 3622–3627. 10.1002/2014GL059524

[rog20214-bib-0094] Gryspeerdt, E. , Stier, P. , & Partridge, D. G. (2014). Satellite observations of cloud regime development: the role of aerosol processes. Atmospheric Chemistry and Physics, 14(3), 1141–1158. 10.5194/acp-14-1141-2014

[rog20214-bib-0095] Gryspeerdt, E. , Stier, P. , & Partridge, D. G. (2014). Links between satellite‐retrieved aerosol and precipitation. Atmospheric Chemistry and Physics, 14(18), 9677–9694. 10.5194/acp-14-9677-2014

[rog20214-bib-1007] Gonzi, S. , Dubovik, O. , Baumgartner, D. , & Putz, E. (2007). Clear‐sky aerosol radiative forcing effects based on multi‐site AERONET observations over europe. Meteorology and Atmospheric Physics, 96, 277–291. 10.1007/s00703-006-0212-9

[rog20214-bib-0096] Halthore, R. N. , Crisp, D. , Schwartz, S. E. , Anderson, G. P. , Berk, A. , Bonnel, B. , Boucher, O. , Chang, F.‐L. , Chou, M.‐D. , Clothiaux, E. E. , Dubuisson, P. , Fomin, B. , Fouquart, Y. , Freidenreich, S. , Gautier, C. , Kato, S. , Laszlo, I. , Li, Z. , Mather, J. H. , Plana‐Fattori, A. , Ramaswamy, V. , Ricchiazzi, P. , Shiren, Y. , Trishchenko, A. , & Wiscombe, W. (2005). Intercomparison of shortwave radiative transfer codes and measurements. Journal of Geophysical Research: Atmospheres, 110(D11), D11206 10.1029/2004JD005293

[rog20214-bib-0097] Hamilton, D. S. , Hantson, S. , Scott, C. E. , Kaplan, J. O. , Pringle, K. J. , Nieradzik, L. P. , Rap, A. , Folberth, G. A. , Spracklen, D. V. , & Carslaw, K. S. (2018). Reassessment of pre‐industrial fire emissions strongly affects anthropogenic aerosol forcing. Nature Communications, 9(1), 3182 10.1038/s41467-018-05592-9 PMC6085333 30093678

[rog20214-bib-0098] Han, Q. , Rossow, W. B. , Zeng, J. , & Welch, R. (2002). Three different behaviors of liquid water path of water clouds in aerosol‐cloud interactions. Journal of the Atmospheric Sciences, 59, 726–735. 10.1175/1520-0469(2002)059<0726:TDBOLW>2.0.CO;2

[rog20214-bib-0099] Hansen, J. , Sato, M. , & Ruedy, R. (1997). Radiative forcing and climate response. Journal of Geophysical Research, 102(D6), 6831–6864. 10.1029/96JD03436

[rog20214-bib-0100] Hansen, J. , Sato, M. , Ruedy, R. , Nazarenko, L. , Lacis, A. , Schmidt, G. A. , Russell, G. , Aleinov, I. , Bauer, M. , Bauer, S. , Bell, N. , Cairns, B. , Canuto, V. , Chandler, M. , Cheng, Y. , Genio, A. D. , Faluvegi, G. , Fleming, E. , Friend, A. , Hall, T. , Jackman, C. , Kelley, M. , Kiang, N. Y. , Koch, D. , Lean, J. , Lerner, J. , Lo, K. , Menon, S. , Miller, R. L. , Minnis, P. , Novakov, T. , Oinas, V. , Perlwitz, J. P. , Perlwitz, J. , Rind, D. , Romanou, A. , Shindell, D. , Stone, P. , Sun, S. , Tausnev, N. , Thresher, D. , Wielicki, B. , Wong, T. , Yao, M. , & Zhang, S. (2005). Efficacy of climate forcings. Journal of Geophysical Research, 110(D18), D18104 10.1029/2005JD005776

[rog20214-bib-0101] Hansen, J. E. , & Travis, L. D. (1974). Light scattering in planetary atmospheres. Space Science Reviews, 16(4), 527–610. 10.1007/BF00168069

[rog20214-bib-0102] Hartmann, D. , Rusticucci, T. A. M. G. K. M. , Alexander, L. , Brönnimann, S. , Charabi, Y. , Dentener, F. , Dlugokencky, E. , Easterling, D. , Kaplan, A. , Soden, B. , Thorne, P. , Wild, M. , & Zhai, P. (2013). In Stocker T., Qin D., Plattner G.‐K., Tignor M., Allen S., Boschung J., Nauels A., Xia Y., Bex V., & Midgley P. (Eds.), Observations: Atmosphere and surface, in Climate change 2013: The physical science basis. Contribution of Working Group I to the Fifth Assessment Report of the Intergovernmental Panel on Climate Change. Cambridge, United Kingdom and New York, NY, USA, Cambridge, United Kingdom and New York, NY, USA: Cambridge University Press.

[rog20214-bib-0103] Haywood, J. (2015). Atmospheric aerosols and their role in climate change In Letcher T. (Ed.), Climate Change, Observed Impacts on Planet Earth (chap. 27, pp. 449–464). Cambridge, United Kingdom and New York, NY, USA: Elsevier.

[rog20214-bib-0104] Haywood, J. , Francis, P. , Osborne, S. , Glew, M. , Loeb, N. , Highwood, E. , Tanré, D. , Myhre, G. , Formenti, P. , & Hirst, E. (2003). Radiative properties and direct radiative effect of saharan dust measured by the c‐130 aircraft during shade: 1. solar spectrum. Journal of Geophysical Research: Atmospheres, 108(D18), 8577 10.1029/2002JD002687

[rog20214-bib-0105] Haywood, J. M. , Ramaswamy, V. , & Donner, L. J. (1997). A limited‐area‐model case study of the effects of sub‐grid scale variations in relative humidity and cloud upon the direct radiative forcing of sulfate aerosol. Geophysical Research Letters, 24(2), 143–146. 10.1029/96GL03812

[rog20214-bib-0106] Haywood, J. M. , Ramaswamy, V. , & Soden, B. J. (1999). Tropospheric aerosol climate forcing in clear‐sky satellite observations over the oceans. Science, 283(5406), 1299–1303. 10.1126/science.283.5406.1299 10037595

[rog20214-bib-0107] Haywood, J. M. , & Shine, K. P. (1995). The effect of anthropogenic sulfate and soot aerosol on the clear sky planetary radiation budget. Geophysical Research Letters, 22(5), 603–606. 10.1029/95GL00075

[rog20214-bib-0108] Heyn, I. , Block, K. , Mülmenstädt, J. , Gryspeerdt, E. , Kühne, P. , Salzmann, M. , & Quaas, J. (2017). Assessment of simulated aerosol effective radiative forcings in the terrestrial spectrum. Geophysical Research Letters, 44, 1001–1007. 10.1002/2016GL071975

[rog20214-bib-0109] Hill, A. A. , Feingold, G. , & Jiang, H. (2009). The influence of entrainment and mixing assumption on aerosol‐cloud interactions in marine stratocumulus. Journal of the Atmospheric Sciences, 66(5), 1450–1464. 10.1175/2008JAS2909.1

[rog20214-bib-0110] Hobbs, P. V. , Radke, L. F. , & Shumway, S. E. (1970). Cloud condensation nuclei from industrial sources and their apparent influence on precipitation in washington state. Journal of the Atmospheric Sciences, 27(1), 81–89. 10.1175/1520-0469(1970)027<0091:CCNFIS>2.0.CO;2

[rog20214-bib-0111] Hodnebrog, Ø. , Myhre, G. , & Samset, B. (2014). How shorter black carbon lifetime alters its climate effect. Nature Communications, 5(1), 5065 10.1038/ncomms6065 25255429

[rog20214-bib-0112] Hoesly, R. M. , Smith, S. J. , Feng, L. , Klimont, Z. , Janssens‐Maenhout, G. , Pitkanen, T. , Seibert, J. J. , Vu, L. , Andres, R. J. , Bolt, R. M. , Bond, T. C. , Dawidowski, L. , Kholod, N. , Kurokawa, J.‐I. , Li, M. , Liu, L. , Lu, Z. , Moura, M. C. P. , O'Rourke, P. R. , & Zhang, Q. (2018). Historical (1750–2014) anthropogenic emissions of reactive gases and aerosols from the community emissions data system (ceds). Geoscientific Model Development, 11(1), 369–408. 10.5194/gmd-11-369-2018

[rog20214-bib-0113] Holben, B. , Eck, T. , Slutsker, I. , Tanré, D. , Buis, J. , Setzer, A. , Vermote, E. , Reagan, J. , Kaufman, Y. , Nakajima, T. , Lavenu, F. , Jankowiak, I. , & Smirnov, A. (1998). Aeronet—A federated instrument network and data archive for aerosol characterization. Remote Sensing of Environment, 66(1), 1–16. 10.1016/S0034-4257(98)00031-5

[rog20214-bib-0114] Hoose, C. , Lohmann, U. , Stier, P. , Verheggen, B. , & Weingartner, E. (2008). Aerosol processing in mixed‐phase clouds in ECHAM5‐HAM: Model description and comparison to observations. Journal of Geophysical Research: Atmospheres, 113(D7), D07210 10.1029/2007JD009251

[rog20214-bib-0115] Hoose, C. , & Möhler, O. (2012). Heterogeneous ice nucleation on atmospheric aerosols: a review of results from laboratory experiments. Atmospheric Chemistry and Physics, 12(20), 9817–9854. 10.5194/acp-12-9817-2012

[rog20214-bib-1111] Hsu, N. C. , Sayer, A. M. , Jeong, M.‐J. , & Bettenhausen, C. (2013). SeaWiFS Deep Blue Aerosol Optical Depth and Angstrom Exponent Level 2 Data V004, Greenbelt, MD, USA, Goddard Earth Sciences Data and Information Services Center (GES DISC), 10.5067/MEASURES/SWDB/DATA201

[rog20214-bib-0116] Hu, Y. , Rodier, S. , Xu, K.‐m. , Sun, W. , Huang, J. , Lin, B. , Zhai, P. , & Josset, D. (2010). Occurrence, liquid water content, and fraction of supercooled water clouds from combined caliop/iir/modis measurements. Journal of Geophysical Research: Atmospheres, 115(D4), D00H34 10.1029/2009JD012384

[rog20214-bib-0117] Huang, J. P. , Liu, J. J. , Chen, B. , & Nasiri, S. L. (2015). Detection of anthropogenic dust using CALIPSO lidar measurements. Atmospheric Chemistry and Physics, 15(20), 11,653–11,665. 10.5194/acp-15-11653-2015

[rog20214-bib-0118] Jacobson, M. Z. , & Seinfeld, J. H. (2004). Evolution of nanoparticle size and mixing state near the point of emission. Atmospheric Environment, 38(13), 1839–1850. 10.1016/j.atmosenv.2004.01.014

[rog20214-bib-0119] Jensen, E. J. , Diskin, G. , Lawson, R. P. , Lance, S. , Bui, T. P. , Hlavka, D. , McGill, M. , Pfister, L. , Toon, O. B. , & Gao, R. (2013). Ice nucleation and dehydration in the tropical tropopause layer. Proceedings of the National Academy of Sciences of the United States of America, 110(6), 2041–2046. 10.1073/pnas.1217104110 23341619 PMC3568347

[rog20214-bib-0120] Jensen, E. J. , Ueyama, R. , Pfister, L. , Bui, T. V. , Lawson, R. P. , Woods, S. , Thornberry, T. , Rollins, A. W. , Diskin, G. S. , DiGangi, J. P. , & Avery, M. A. (2016). On the susceptibility of cold tropical cirrus to ice nuclei abundance. Journal of the Atmospheric Sciences, 73(6), 2445–2464. 10.1175/JAS-D-15-0274.1

[rog20214-bib-0121] Jiao, C. , Flanner, M. G. , Balkanski, Y. , Bauer, S. E. , Bellouin, N. , Berntsen, T. K. , Bian, H. , Carslaw, K. S. , Chin, M. , De Luca, N. , Diehl, T. , Ghan, S. J. , Iversen, T. , Kirkevåg, A. , Koch, D. , Liu, X. , Mann, G. W. , Penner, J. E. , Pitari, G. , Schulz, M. , Seland, Ø. , Skeie, R. B. , Steenrod, S. D. , Stier, P. , Takemura, T. , Tsigaridis, K. , van Noije, T. , Yun, Y. , & Zhang, K. (2014). An AeroCom assessment of black carbon in Arctic snow and sea ice. Atmospheric Chemistry and Physics, 14(5), 2399–2417. 10.5194/acp-14-2399-2014

[rog20214-bib-0122] Jing, X. , & Suzuki, K. (2018). The impact of process‐based warm rain constraints on the aerosol indirect effect. Geophysical Research Letters. 10.1029/2018GL079956

[rog20214-bib-0123] Johnson, B. T. , Shine, K. P. , & Forster, P. M. (2004). The semi‐direct aerosol effect: Impact of absorbing aerosols on marine stratocumulus. Quart. J. Roy. Meteorol. Soc., 130(599), 1407–1422. 10.1256/qj.03.61

[rog20214-bib-0124] Johnson, J. S. , Regayre, L. A. , Yoshioka, M. , Pringle, K. J. , Lee, L. A. , Sexton, D. M. H. , Rostron, J. W. , Booth, B. B. B. , & Carslaw, K. S. (2018). The importance of comprehensive parameter sampling and multiple observations for robust constraint of aerosol radiative forcing. Atmospheric Chemistry and Physics, 18(17), 13,031–13,053. 10.5194/acp-18-13031-2018

[rog20214-bib-0125] Jones, G. S. , Stott, P. A. , & Mitchell, J. F. B. (2016). Uncertainties in the attribution of greenhouse gas warming and implications for climate prediction. Journal of Geophysical Research: Atmospheres, 121(12), 6969–6992. 10.1002/2015JD024337

[rog20214-bib-0126] Kacenelenbogen, M. S. , Vaughan, M. A. , Redemann, J. , Young, S. A. , Liu, Z. , Hu, Y. , Omar, A. H. , LeBlanc, S. , Shinozuka, Y. , Livingston, J. , Zhang, Q. , & Powell, K. A. (2019). Estimations of global shortwave direct aerosol radiative effects above opaque water clouds using a combination of A‐Train satellite sensors. Atmospheric Chemistry and Physics, 19(7), 4933–4962. 10.5194/acp-19-4933-2019

[rog20214-bib-0127] Kanitz, T. , Seifert, P. , Ansmann, A. , Engelmann, R. , Althausen, D. , Casiccia, C. , & Rohwer, E. G. (2011). Contrasting the impact of aerosols at northern and southern midlatitudes on heterogeneous ice formation. Geophysical Research Letters, 38, n/a 10.1029/2011GL048532

[rog20214-bib-0128] Kanji, Z. A. , Ladino, L. A. , Wex, H. , Boose, Y. , Burkert‐Kohn, M. , Cziczo, D. J. , & Krämer, M. (2017). Overview of ice nucleating particles. Meteorological Monographs, 58, 1.1–1.33. 10.1175/AMSMONOGRAPHS-D-16-0006.1

[rog20214-bib-0129] Kärcher, B. , & Lohmann, U. (2003). A parameterization of cirrus cloud formation: Heterogeneous freezing. Journal of Geophysical Research, 108(D14), 4402 10.1029/2002JD003220

[rog20214-bib-1018] Kasoar, M. , Shawki, D. , & Voulgarikis, A. (2018). Similar spatial patterns of global climate response to aerosols from different regions. npj Climate and Atmospheric Science, 1(12), 1–8. 10.1038/s41612-018-0022-z

[rog20214-bib-0130] Kaufman, Y. J. , & Koren, I. (2006). Smoke and pollution aerosol effect on cloud cover. Science, 313(5787), 655–658. 10.1126/science.1126232 16840661

[rog20214-bib-0131] Kaufman, Y. J. , Koren, I. , Remer, L. A. , Rosenfeld, D. , & Rudich, Y. (2005). The effect of smoke, dust, and pollution aerosol on shallow cloud development over the atlantic ocean. Proceedings of the National Academy of Sciences of the United States of America, 102(32), 11,207–11,212. 10.1073/pnas.0505191102 PMC1182178 16076949

[rog20214-bib-0132] Kay, J. E. , & Wood, R. (2008). Timescale analysis of aerosol sensitivity during homogeneous freezing and implications for upper tropospheric water vapor budgets. Geophysical Research Letters, 35, L10809 10.1029/2007GL032628

[rog20214-bib-0133] Keil, A. , & Haywood, J. M. (2003). Solar radiative forcing by biomass burning aerosol particles during safari 2000: A case study based on measured aerosol and cloud properties. Journal of Geophysical Research: Atmospheres, 108(D13), n/a 10.1029/2002JD002315

[rog20214-bib-0134] Khain, A. P. , Rosenfeld, D. , & Pokrovsky, A. (2001). Simulating convective clouds with sustained supercooled liquid water down to −37.5^∘^C using a spectral microphysics model. Geophysical Research Letters, 28(20), 3887–3890. 10.1029/2000GL012662

[rog20214-bib-0135] Kim, B.‐G. , Miller, M. A. , Schwartz, S. E. , Liu, Y. , & Min, Q. (2008). The role of adiabaticity in the aerosol first indirect effect. Journal of Geophysical Research: Atmospheres, 113(D5), n/a 10.1029/2007JD008961

[rog20214-bib-0136] Kim, B.‐G. , Schwartz, S. E. , Miller, M. A. , & Min, Q. (2003). Effective radius of cloud droplets by ground‐based remote sensing: Relationship to aerosol. Journal of Geophysical Research: Atmospheres, 108(D23), 4740 10.1029/2003JD003721

[rog20214-bib-0137] King, M. D. , Kaufman, Y. J. , Tané, D. , & Nakajima, T. (1999). Remote sensing of tropospheric aerosols from space: Past, present, and future. Bull. Amer. Meteorol. Soc., 80(11), 2229–2259. 10.1175/1520-0477(1999)080<2229:RSOTAF>2.0.CO;2

[rog20214-bib-0138] Kinne, S. (2019). Aerosol radiative effects with MACv2. Atmospheric Chemistry and Physics, 19(16), 10,919–10,959. 10.5194/acp-19-10919-2019

[rog20214-bib-0139] Kinne, S. , O'Donnel, D. , Stier, P. , Kloster, S. , Zhang, K. , Schmidt, H. , Rast, S. , Giorgetta, M. , Eck, T. F. , & Stevens, B. (2013). MAC‐v1: A new global aerosol climatology for climate studies. Journal of Advances in Modeling Earth Systems, 5, 704–740. 10.1002/jame.20035

[rog20214-bib-0140] Kinne, S. , Schulz, M. , Textor, C. , Guibert, S. , Balkanski, Y. , Bauer, S. E. , Berntsen, T. , Berglen, T. , Boucher, O. , Chin, M. , Collins, W. , Dentener, F. , Diehl, T. , Easter, R. , Feichter, H. , Fillmore, D. , Ghan, S. , Ginoux, P. , Gong, S. , Grini, A. , Hendricks, J. , Herzog, M. , Horrowitz, L. , Isaksen, I. , Iversen, T. , Kirkevåg, A. , Kloster, S. , Koch, D. , Kristjansson, J. E. , Krol, M. , Lauer, A. , Lamarque, J. F. , Lesins, G. , Liu, X. , Lohmann, U. , Montanaro, V. , Myhre, G. , Penner, J. , Pitari, G. , Reddy, S. , Seland, Ø. , Stier, P. , Takemura, T. , & Tie, X. (2006). An aerocom initial assessment optical properties in aerosol component modules of global models. Atmospheric Chemistry and Physics, 6(7), 1815–1834. 10.5194/acp-6-1815-2006

[rog20214-bib-0141] Kipling, Z. , Stier, P. , Schwarz, J. P. , Perring, A. E. , Spackman, J. R. , Mann, G. W. , Johnson, C. E. , & Telford, P. J. (2013). Constraints on aerosol processes in climate models from vertically‐resolved aircraft observations of black carbon. Atmospheric Chemistry and Physics, 13(12), 5969–5986. 10.5194/acp-13-5969-2013

[rog20214-bib-0142] Kirkby, J. , Duplissy, J. , Sengupta, K. , Frege, C. , Gordon, H. , Williamson, C. , Heinritzi, M. , Simon, M. , Yan, C. , Almeida, J. , Tröstl, J. , Nieminen, T. , Ortega, I. , Wagner, R. , Adamov, A. , Amorim, A. , Bernhammer, A.‐K. , Bianchi, F. , Breitenlechner, M. , Brilke, S. , Chen, X. , Craven, J. , Dias, A. , Ehrhart, S. , Flagan, R. , Franchin, A. , Fuchs, C. , Guida, R. , Hakala, J. , Hoyle, C. , Jokinen, T. , Junninen, H. , Kangasluoma, J. , Kim, J. , Krapf, M. , Kürten, A. , Laaksonen, A. , Lehtipalo, K. , Makhmutov, V. , Mathot, S. , Molteni, U. , Onnela, A. , Peräkylä, O. , Piel, F. , Petäjä, T. , Praplan, A. , Pringle, K. , Rap, A. , Richards, N. , Riipinen, I. , Rissanen, M. , Rondo, L. , Sarnela, N. , Schobesberger, S. , Scott, C. , Seinfeld, J. , Sipilä, M. , Steiner, G. , Stozhkov, Y. , Stratmann, F. , Tome, A. , Virtanen, A. , Vogel, A. , Wagner, A. , Wagner, P. , Weingartner, E. , Wimmer, D. , Winkler, P. , Ye, P. , Zhang, X. , Hansel, A. , Dommen, J. , Donahue, N. , Worsnop, D. , Baltensperger, U. , Kulmala, M. , Carslaw, K. , & Curtius, J. (2016). Ion‐induced nucleation of pure biogenic particles. Nature, 533(7604), 521–526. 10.1038/nature17953 27225125 PMC8384037

[rog20214-bib-0143] Kittaka, C. , Winker, D. M. , Vaughan, M. A. , Omar, A. , & Remer, L. A. (2011). Intercomparison of column aerosol optical depths from CALIPSO and MODIS‐Aqua. Atmospheric Measurement Techniques, 4(2), 131–141. 10.5194/amt-4-131-2011

[rog20214-bib-0144] Klein, S. A. , & Hall, A. (2015). Emergent constraints for cloud feedbacks. Current Climate Change Reports, 1(4), 276–287. 10.1007/s40641-015-0027-1

[rog20214-bib-0145] Knutti, R. , Rugenstein, M. A. A. , & Hegerl, G. C. (2017). Beyond equilibrium climate sensitivity. Nature Geoscience, 10(10), 727–736. 10.1038/ngeo3017

[rog20214-bib-0146] Knutti, R. , Stocker, T. F. , Joos, F. , & Plattner, G.‐K. (2002). Constraints on radiative forcing and future climate change from observations and climate model ensembles. Nature, 416(6882), 719–723. 10.1038/416719a 11961550

[rog20214-bib-0147] Koch, D. , & Del Genio, A. D. (2010). Black carbon absorption effects on cloud cover: Review and synthesis. Atmospheric Chemistry and Physics, 10(16), 7685–7696. 10.5194/acp-10-7685-2010

[rog20214-bib-0148] Koffi, B. , Schulz, M. , Bréon, F.‐M. , Dentener, F. , Steensen, B. M. , Griesfeller, J. , Winker, D. , Balkanski, Y. , Bauer, S. E. , Bellouin, N. , Berntsen, T. , Bian, H. , Chin, M. , Diehl, T. , Easter, R. , Ghan, S. , Hauglustaine, D. A. , Iversen, T. , Kirkevåg, A. , Liu, X. , Lohmann, U. , Myhre, G. , Rasch, P. , Seland, Ø. , Skeie, R. B. , Steenrod, S. D. , Stier, P. , Tackett, J. , Takemura, T. , Tsigaridis, K. , Vuolo, M. R. , Yoon, J. , & Zhang, K. (2016). Evaluation of the aerosol vertical distribution in global aerosol models through comparison against CALIOP measurements: AeroCom phase II results. Journal of Geophysical Research: Atmospheres, 121(12), 7254–7283. 10.1002/2015JD024639 PMC7430518 32818126

[rog20214-bib-0149] Köhler, H. (1936). The nucleus in and the growth of hygroscopic droplets. Transactions of the Faraday Society, 32(0), 1152–1161. 10.1039/TF9363201152

[rog20214-bib-0151] Kok, J. F. , Ward, D. S. , Mahowald, N. M. , & Evan, A. T. (2018). Global and regional importance of the direct dust‐climate feedback. Nature Communications, 9(1), 241 10.1038/s41467-017-02620-y PMC5770443 29339783

[rog20214-bib-0152] Koren, I. , Feingold, G. , & Remer, L. A. (2010). The invigoration of deep convective clouds over the Atlantic: Aerosol effect, meteorology or retrieval artifact? Atmospheric Chemistry and Physics, 10(18), 8855–8872. 10.5194/acp-10-8855-2010

[rog20214-bib-0153] Koren, I. , Kaufman, Y. J. , Remer, L. A. , & Martins, J. V. (2004). Measurement of the effect of amazon smoke on inhibition of cloud formation. Science, 303(5662), 1342–1345. 10.1126/science.1089424 14988557

[rog20214-bib-0154] Koren, I. , Kaufman, Y. J. , Rosenfeld, D. , Remer, L. A. , & Rudich, Y. (2005). Aerosol invigoration and restructuring of atlantic convective clouds. Geophysical Research Letters, 32, 14828 10.1029/2005GL023187

[rog20214-bib-0155] Koren, I. , Remer, L. A. , Altaratz, O. , Martins, J. V. , & Davidi, A. (2010). Aerosol‐induced changes of convective cloud anvils produce strong climate warming. Atmospheric Chemistry and Physics, 10(10), 5001–5010. 10.5194/acp-10-5001-2010

[rog20214-bib-0156] Krämer, M. , Rolf, C. , Luebke, A. , Afchine, A. , Spelten, N. , Costa, A. , Meyer, J. , Zoger, M. , Smith, J. , Herman, R. L. , Buchholz, B. , Ebert, V. , Baumgardner, D. , Borrmann, S. , Klingebiel, M. , & Avallone, L. (2016). A microphysics guide to cirrus clouds ‐ part 1: Cirrus types. Atmospheric Chemistry and Physics, 16(5), 3463–3483. 10.5194/acp-16-3463-2016

[rog20214-bib-0157] Kravitz, B. , Robock, A. , Tilmes, S. , Boucher, O. , English, J. , Irvine, P. , Jones, A. , Lawrence, M. G. , MacCracken, M. , Muri, H. , Moore, J. C. , Niemeier, U. , Phipps, S. J. , Sillmann, J. , Storelvmo, T. , Wang, H. , & Watanabe, S. (2015). The Geoengineering Model Intercomparison Project Phase 6 (GeoMIP6): Simulation design and preliminary results. Geoscientific Model Development, 8, 3379–3392.

[rog20214-bib-0158] Kretzschmar, J. , Salzmann, M. , Mülmenstädt, J. , Boucher, O. , & Quaas, J. (2017). Comment on “rethinking the lower bound on aerosol radiative forcing”. Journal of Climate, 30, 6579–6584. 10.1175/JCLI-D-16-0668.1

[rog20214-bib-1008] Kristiansen, N. I. , Stohl, A. , & Wotawa, G. (2012). Atmospheric removal times of the aerosol‐bound radionuclides 137cs and 131i measured after the Fukushima Dai‐ichi nuclear accident ‐ a constraint for air quality and climate models. Atmospheric Chemistry and Physics, 12(22), 10,759–10,769. 10.5194/acp-12-10759-2012

[rog20214-bib-0159] Kuebbeler, M. , Lohmann, U. , Hendricks, J. , & Kärcher, B. (2014). Dust ice nuclei effects on cirrus clouds. Atmospheric Chemistry and Physics, 14, 3027–3046. 10.5194/acp-14-3027-2014

[rog20214-bib-1009] Kulmala, M. , Asmi, A. , Lappalainen, H. K. , Baltensperger, U. , Brenguier, J.‐L. , & Facchini, M. C. (2011). Pandis, S. N General overview: European integrated project on aerosol cloud climate and air quality interactions (eucaari); integrating aerosol research from nano to global scales. Atmospheric Chemistry and Physics, 11(4), 13,061–13,143. 10.5194/acp-11-13061-2011

[rog20214-bib-1010] Lamarque, J.‐F. , Bond, T. C. , Eyring, V. , Granier, C. , Heil, A. , & Klimont, Z. (2010). van Vuuren, D. P Historical (1850‐2000) gridded anthropogenic and biomass burning emissions of reactive gases and aerosols: methodology and application. Atmospheric Chemistry and Physics, 10(15), 7017–7039. 10.5194/acp-10-7017-2010

[rog20214-bib-1011] Leahy, L. V. , Anderson, T. L. , Eck, T. F. , & Bergstrom, R. W. (2007). A synthesis of single scattering albedo of biomass burning aerosol over southern africa during SAFARI 2000. Geophysical Research Letters, 34(12). 10.1029/2007GL029697

[rog20214-bib-0160] Lebsock, M. , Stephens, G. , & Kummerow, C. (2008). Multisensor satellite observations of aerosol effects on warm clouds. Journal of Geophysical Research, 113, D15,205 10.1029/2008JD009876

[rog20214-bib-0161] Lee, L. A. , Carslaw, K. S. , Pringle, K. J. , Mann, G. W. , & Spracklen, D. V. (2011). Emulation of a complex global aerosol model to quantify sensitivity to uncertain parameters. Atmospheric Chemistry and Physics, 11(23), 12,253–12,273. 10.5194/acp-11-12253-2011

[rog20214-bib-0162] Lee, Y. H. , Adams, P. J. , & Shindell, D. T. (2015). Evaluation of the global aerosol microphysical ModelE2‐TOMAS model against satellite and ground‐based observations. Geoscientific Model Development, 8(3), 631–667. 10.5194/gmd-8-631-2015

[rog20214-bib-0163] Levy, R. C. , Mattoo, S. , Munchak, L. A. , Remer, L. A. , Sayer, A. M. , Patadia, F. , & Hsu, N. C. (2013). The collection 6 MODIS aerosol products over land and ocean. Atmospheric Measurement Techniques, 6(11), 2989–3034. 10.5194/amt-6-2989-2013

[rog20214-bib-0164] Li, J. , Jian, B. , Huang, J. , Hu, Y. , Zhao, C. , Kawamoto, K. , Liao, S. , & Wu, M. (2018). Long‐term variation of cloud droplet number concentrations from space‐based lidar. Remote Sensing of Environment, 213, 144–161.

[rog20214-bib-0165] Liu, X. , Easter, R. C. , Ghan, S. J. , Zaveri, R. , Rasch, P. , Shi, X. , Lamarque, J.‐F. , Gettelman, A. , Morrison, H. , Vitt, F. , Conley, A. , Park, S. , Neale, R. , Hannay, C. , Ekman, A. M. L. , Hess, P. , Mahowald, N. , Collins, W. , Iacono, M. J. , Bretherton, C. S. , Flanner, M. G. , & Mitchell, D. (2012). Toward a minimal representation of aerosols in climate models: description and evaluation in the Community Atmosphere Model CAM5. Geoscientific Model Development, 5(3), 709–739. 10.5194/gmd-5-709-2012

[rog20214-bib-0166] Loeb, N. , Manalo‐Smith, N. , Su, W. , Shankar, M. , & Thomas, S. (2016). CERES top‐of‐atmosphere earth radiation budget climate data record: Accounting for in‐orbit changes in instrument calibration. Remote Sensing, 8, 182 10.3390/rs8030182

[rog20214-bib-0167] Loeb, N. G. , & Schuster, G. L. (2008). An observational study of the relationship between cloud, aerosol and meteorology in broken low‐level cloud conditions. Journal of Geophysical Research, 113, 14,214 10.1029/2007JD009763

[rog20214-bib-0168] Lohmann, U. (2002). A glaciation indirect aerosol effect caused by soot aerosols. Geophysical Research Letters, 29(4). 10.1029/2001GL014357

[rog20214-bib-0169] Lohmann, U. (2008). Global anthropogenic aerosol effects on convective clouds in ECHAM5*‐*HAM. Atmospheric Chemistry and Physics, 8(7), 2115–2131. 10.5194/acp-8-2115-2008

[rog20214-bib-0170] Lohmann, U. (2017). Anthropogenic aerosol influences on mixed‐phased clouds. Curr. Clim. Change Rep., 3, 32–44. 10.1007/s40641-017-0059-9

[rog20214-bib-0171] Lohmann, U. , & Feichter, J. (2001). Can the direct and semi‐direct aerosol effect compete with the indirect effect on a global scale? Geophysical Research Letters, 28(1), 159–161. 10.1029/2000GL012051

[rog20214-bib-0172] Lohmann, U. , & Kärcher, B. (2002). First interactive simulations of cirrus clouds formed by homogeneous freezing in the ECHAM general circulation model. Journal of Geophysical Research, 107(D10). 10.1029/2001JD000767

[rog20214-bib-1012] Lohmann, U. , & Lesins, G. (2002). Stronger constraints on the anthropogenic indirect aerosol effect. Science, 298(5595), 1012–1015. 10.1126/science.1075405 12411701

[rog20214-bib-0173] Lowenthal, D. H. , Borys, R. D. , Choularton, T. W. , Bower, K. N. , Flynn, M. J. , & Gallagher, M. W. (2004). Parameterization of the cloud droplet‐sulfate relationship. Atmospheric Environment, 38(2), 287–292. 10.1016/j.atmosenv.2003.09.046

[rog20214-bib-1013] Lu, M.‐L. , Sorooshian, A. , Jonsson, H. H. , Feingold, G. , Flagan, R. C. , & Seinfeld, J. H. (2009). Marine stratocumulus aerosol‐cloud relationships in the MASE‐II experiment: Precipitation susceptibility in eastern Pacifc marine stratocumulus. Journal of Geophysical Research, 114, D24203 10.1029/2009JD012774

[rog20214-bib-1114] Lund, M.‐T. , Myhre, G. , & Samset, B. H. (2019). Anthropogenic aerosol forcing under the Shared Socioeconomic Pathways. Atmospheric Chemistry and Physics, 19, 13827–13839. 10.5194/acp-19-13827-2019

[rog20214-bib-0174] Ma, P.‐L. , Rasch, P. J. , Chepfer, H. , Winker, D. M. , & Ghan, S. J. (2018). Observational constraint on cloud susceptibility weakened by aerosol retrieval limitations. Nature Communications, 9(1), 2640 10.1038/s41467-018-05028-4 PMC6035237 29980669

[rog20214-bib-0175] Mace, G. G. , & Abernathy, A. C. (2016). Observational evidence for aerosol invigoration in shallow cumulus downstream of Mount Kilauea. Geophysical Research Letters, 43, 2981–2988. 10.1002/2016GL067830

[rog20214-bib-0176] Mahowald, N. M. (2007). Anthropocene changes in desert area: Sensitivity to climate model predictions. Geophysical Research Letters, 34 10.1029/2007GL030472

[rog20214-bib-0177] Mahowald, N. M. , & Luo, C. (2003). A less dusty future? Geophysical Research Letters, 30(17). 10.1029/2003GL017880

[rog20214-bib-0178] Mahrt, F. , Marcolli, C. , David, R. O. , Grönquist, P. , Barthazy Meier, E. J. , Lohmann, U. , & Kanji, Z. A. (2018). Ice nucleation abilities of soot particles determined with the horizontal ice nucleation chamber. Atmospheric Chemistry and Physics, 18(18), 13,363–13,392. 10.5194/acp-18-13363-2018

[rog20214-bib-0179] Malavelle, F. F. , Haywood, J. M. , Jones, A. , Gettelman, A. , Clarisse, L. , Bauduin, S. , Allan, R. P. , Karset, I. H. H. , Kristjánsson, J. E. , Oreopoulos, L. , Cho, N. , Lee, D. , Bellouin, N. , Boucher, O. , Grosvenor, D. P. , Carslaw, K. S. , Dhomse, S. , Mann, G. W. , Schmidt, A. , Coe, H. , Hartley, M. E. , Dalvi, M. , Hill, A. A. , Johnson, B. T. , Johnson, C. E. , Knight, J. R. , O'Connor, F. M. , Partridge, D. G. , Stier, P. , Myhre, G. , Platnick, S. , Stephens, G. L. , Takahashi, H. , & Thordarson, T. (2017). Strong constraints on aerosol‐cloud interactions from volcanic eruptions. Nature, 546(7659), 485–491. 10.1038/nature22974 28640263

[rog20214-bib-0181] Marlon, J. R. , Kelly, R. , Daniau, A.‐L. , Vannière, B. , Power, M. J. , Bartlein, P. , Higuera, P. , Blarquez, O. , Brewer, S. , Brücher, T. , Feurdean, A. , Romera, G. G. , Iglesias, V. , Maezumi, S. Y. , Magi, B. , Courtney Mustaphi, C. J. , & Zhihai, T. (2016). Reconstructions of biomass burning from sediment‐charcoal records to improve data–model comparisons. Biogeosciences, 13(11), 3225–3244. 10.5194/bg-13-3225-2016

[rog20214-bib-0182] Martin, G. M. , Johnson, D. W. , & Spice, A. (1994). The measurement and parameterization of effective radius of droplets in warm stratocumulus clouds. Journal of the Atmospheric Sciences, 51(13), 1823–1842. 10.1175/1520-0469(1994)051<1823:TMAPOE>2.0.CO;2

[rog20214-bib-0183] Mauger, G. S. , & Norris, J. R. (2007). Meteorological bias in satellite estimates of aerosol‐cloud relationships. Geophysical Research Letters, 34, L16824 10.1029/2007GL029952

[rog20214-bib-0184] McComiskey, A. , & Feingold, G. (2012). The scale problem in quantifying aerosol indirect effects. Atmospheric Chemistry and Physics, 12, 1031–1049. 10.5194/acp-12-1031-2012 PMC7447155 32849860

[rog20214-bib-0185] McComiskey, A. , Feingold, G. , Frisch, A. S. , Turner, D. D. , Miller, M. A. , Chiu, J. C. , Min, Q. , & Ogren, J. A. (2009). An assessment of aerosol‐cloud interactions in marine stratus clouds based on surface remote sensing. Journal of Geophysical Research: Atmospheres, 114(D9). 10.1029/2008JD011006

[rog20214-bib-5000] McCormick, R. A. , & Ludwig, J. H. (1967). Climate modification by atmospheric aerosols. Science, 156 (3780), 1358–1359. 10.1126/science.156.3780.1358 4886897

[rog20214-bib-0186] McCoy, D. T. , Bender, F. A. M. , Grosvenor, D. P. , Mohrmann, J. K. , Hartmann, D. L. , Wood, R. , & Field, P. R. (2018). Predicting decadal trends in cloud droplet number concentration using reanalysis and satellite data. Atmospheric Chemistry and Physics, 18(3), 2035–2047. 10.5194/acp-18-2035-2018

[rog20214-bib-0187] McCoy, D. T. , Bender, F. A.‐M. , Mohrmann, J. K. C. , Hartmann, D. L. , Wood, R. , & Grosvenor, D. P. (2017). The global aerosol‐cloud first indirect effect estimated using MODIS, MERRA, and AeroCom. Journal of Geophysical Research, 122, 1779–1796. 10.1002/2016JD026141

[rog20214-bib-0188] McCoy, D. T. , Field, P. R. , Schmidt, A. , Grosvenor, D. P. , Bender, F. A.‐M. , Shipway, B. J. , Hill, A. A. , Wilkinson, J. M. , & Elsaesser, G. S. (2018). Aerosol midlatitude cyclone indirect effects in observations and high‐resolution simulations. Atmospheric Chemistry and Physics, 18(8), 5821–5846. 10.5194/acp-18-5821-2018

[rog20214-bib-0189] McCoy, D. T. , & Hartmann, D. L. (2015). Observations of a substantial cloud‐aerosol indirect effect during the 2014‐2015 Bàrdarbunga‐Veidivötn fissure eruption in Iceland. Geophysical Research Letters, 42, 10,409–10,414. 10.1002/2015GL067070

[rog20214-bib-0190] McFiggans, G. , Artaxo, P. , Baltensperger, U. , Coe, H. , Facchini, M. C. , Feingold, G. , Fuzzi, S. , Gysel, M. , Laaksonen, A. , Lohmann, U. , Mentel, T. F. , Murphy, D. M. , O'Dowd, C. D. , Snider, J. R. , & Weingartner, E. (2006). The effect of physical and chemical aerosol properties on warm cloud droplet activation. Atmospheric Chemistry and Physics, 6, 2593–2649. 10.5194/acp-6-2593-2006

[rog20214-bib-0191] Michibata, T. , Suzuki, K. , Sato, Y. , & Takemura, T. (2016). The source of discrepancies in aerosol‐cloud‐precipitation interactions between GCM and A‐Train retrievals. Atmospheric Chemistry and Physics, 16, 15,413–15,424. 10.5194/acp-16-15413-2016

[rog20214-bib-0192] Mitchell, D. L. , Garnier, A. , Pelon, J. , & Erfani, E. (2018). CALIPSO (IIR–CALIOP) retrievals of cirrus cloud ice‐particle concentrations. Atmospheric Chemistry and Physics, 18(23), 17,325–17,354. 10.5194/acp-18-17325-2018 PMC6818510 31662738

[rog20214-bib-5002] Mitchell, J. M. J. (1971). The effect of atmospheric aerosols on climate with special reference to temperature near the earth's surface. Journal of Applied Meteorology and Climatology, 10, 703–714.

[rog20214-bib-0193] Morgan, M. G. , Adams, P. J. , & Keith, D. W. (2006). Elicitation of expert judgments of aerosol forcing. Climatic Change, 75, 195 10.1007/s10584-005-9025-y

[rog20214-bib-0194] Mülmenstädt, J. , & Feingold, G. (2018). The radiative forcing of aerosol–cloud interactions in liquid clouds: Wrestling and embracing uncertainty. Current Climate Change Reports, 4(1), 23–40. 10.1007/s40641-018-0089-y

[rog20214-bib-0195] Mülmenstädt, J. , Gryspeerdt, E. , Salzmann, M. , Ma, P.‐L. , Dipu, S. , & Quaas, J. (2019). Separating radiative forcing by aerosol‐cloud interactions and fast cloud adjustments in the ECHAM‐HAMMOZ aerosol‐climate model using the method of partial radiative perturbations. Atmospheric Chemistry and Physics Discussions, 1–20. 10.5194/acp-2018-1304

[rog20214-bib-0196] Murphy, D. M. , Solomon, S. , Portmann, R. W. , Rosenlof, K. H. , Forster, P. M. , & Wong, T. (2009). An observationally based energy balance for the Earth since 1950. Journal of Geophysical Research: Atmospheres, 114(D17). 10.1029/2009JD012105

[rog20214-bib-0197] Myhre, G. , W. Aas , R. Cherian , W. Collins , G. Faluvegi , M. Flanner , P. Forster , O. Hodnebrog , Z. Klimont , M. T. Lund , J. Mülmenstädt , C. Lund Myhre , D. Olivié , M. Prather , J. Quaas , B. H. Samset , J. L. Schnell , M. Schulz , D. Shindell , R. B. Skeie , T. Takemura , and S. Tsyro (2017), Multi‐model simulations of aerosol and ozone radiative forcing due to anthropogenic emission changes during the period 1990–2015, Atmospheric Chemistry and Physics, 17, 2709–2720, doi:10.5194/acp-17-2709-2017.

[rog20214-bib-0198] Myhre, G. , Boucher, O. , Bréon, F.‐M. , Forster, P. , & Shindell, D. (2015). Declining uncertainty in transient climate response as CO2 forcing dominates future climate change. Nature Geoscience, 8, 181–185. 10.1038/ngeo2371

[rog20214-bib-0199] Myhre, G. , Kramer, R. J. , Smith, C. J. , Hodnebrog, Ø. , Forster, P. , Soden, B. J. , Samset, B. H. , Stjern, C. W. , Andrews, T. , Boucher, O. , Faluvegi, G. , Fläschner, D. , Kasoar, M. , Kirkevåg, A. , Lamarque, J.‐F. , Olivié, D. , Richardson, T. , Shindell, D. , Stier, P. , Takemura, T. , Voulgarakis, A. , & Watson‐Parris, D. (2018). Quantifying the importance of rapid adjustments for global precipitation changes. Geophysical Research Letters, 45(20), 11,399–11,405. 10.1029/2018GL079474 PMC6360531 30774164

[rog20214-bib-0200] Myhre, G. , Samset, B. H. , Schulz, M. , Balkanski, Y. , Bauer, S. , Berntsen, T. K. , Bian, H. , Bellouin, N. , Chin, M. , Diehl, T. , Easter, R. C. , Feichter, J. , Ghan, S. J. , Hauglustaine, D. , Iversen, T. , Kinne, S. , Kirkevåg, A. , Lamarque, J.‐F. , Lin, G. , Liu, X. , Lund, M. T. , Luo, G. , Ma, X. , van Noije, T. , Penner, J. E. , Rasch, P. J. , Ruiz, A. , Seland, O. , Skeie, R. B. , Stier, P. , Takemura, T. , Tsigaridis, K. , Wang, P. , Wang, Z. , Xu, L. , Yu, H. , Yu, F. , Yoon, J.‐H. , Zhang, K. , Zhang, H. , & Zhou, C. (2013). Radiative forcing of the direct aerosol effect from AeroCom phase II simulations. Atmospheric Chemistry and Physics, 13, 1853–1877. 10.5194/acp-13-1853-2013

[rog20214-bib-0201] Myhre, G. , D. Shindell , F.‐M. Bréon , W. Collins , J. Fuglestvedt , J. Huang , D. Koch , J.‐F. Lamarque , D. Lee , B. Mendoza , T. Nakajima , A. Robock , G. Stephens , T. Takemura , and H. Zhang (2013), Anthropogenic and natural radiative forcing, in Climate change 2013: The physical science basis. Contribution of Working Group I to the Fifth Assessment Report of the Intergovernmental Panel on Climate Change, edited by Stocker T., Qin D., Plattner G.‐K., Tignor M., Allen S., Boschung J., Nauels A., Xia Y., Bex V., and Midgley P., chap. 8, pp. 659–740, Cambridge University Press, Cambridge, United Kingdom and New York, NY, USA, doi:10.1017/CBO9781107415324.018.

[rog20214-bib-0202] Nakajima, T. , Higurashi, A. , Kawamoto, K. , & Penner, J. E. (2001). A possible correlation between satellite‐derived cloud and aerosol microphysical parameters. Geophysical Research Letters, 28, 1171 10.1029/2000GL012186

[rog20214-bib-0203] Nakajima, T. , & Schulz, M. (2009). What do we know about large‐scale changes of aerosols, clouds, and the radiation budget? Clouds in the Perturbed Climate System. Proceedings Ernst Strüngmann Forum. 10.7551/mitpress/9780262012874.003.0017

[rog20214-bib-0204] Nam, C. , Bony, S. , Dufresne, J.‐L. , & Chepfer, H. (2012). The “too few, too bright” tropical low‐cloud problem in CMIP5 models. Geophysical Research Letters, 39, L21801 10.1029/2012GL053421

[rog20214-bib-0205] Nemesure, S. , Wagener, R. , & Schwartz, S. E. (1995). Direct shortwave forcing of climate by the anthropogenic sulfate aerosol: Sensitivity to particle size, composition, and relative humidity. Journal of Geophysical Research: Atmospheres, 100(D12), 26,105–26,116. 10.1029/95JD02897

[rog20214-bib-0206] Neubauer, D. , Christensen, M. W. , Poulsen, C. A. , & Lohmann, U. (2017). Unveiling aerosol‐cloud interactions—Part 2: Minimising the effects of aerosol swelling and wet scavenging in ECHAM6‐HAM2 for comparison to satellite data. Atmospheric Chemistry and Physics, 17(21), 13,165–13,185. 10.5194/acp-17-13165-2017

[rog20214-bib-0207] Neubauer, D. , Lohmann, U. , Hoose, C. , & Frontoso, M. G. (2014). Impact of the representation of marine stratocumulus clouds on the anthropogenic aerosol effect. Atmospheric Chemistry and Physics, 14(21), 11,997–12,022. 10.5194/acp-14-11997-2014

[rog20214-bib-0208] Norgren, M. S. , de Boer, G. , & Shupe, M. D. (2018). Observed aerosol suppression of cloud ice in low‐level Arctic mixed‐phase clouds. Atmospheric Chemistry and Physics, 18(18), 13,345–13,361. 10.5194/acp-18-13345-2018

[rog20214-bib-0209] Oikawa, E. , Nakajima, T. , & Winker, D. (2018). An evaluation of the shortwave direct aerosol radiative forcing using CALIOP and MODIS observations. Journal of Geophysical Research: Atmospheres, 123(2), 1211–1233. 10.1002/2017JD027247

[rog20214-bib-0210] Oreopoulos, L. , Cho, N. , Lee, D. , & Kato, S. (2016). Radiative effects of global modis cloud regimes. Journal of Geophysical Research: Atmospheres, 121(5), 2299–2317. 10.1002/2015JD024502 PMC5880056 29619289

[rog20214-bib-0211] Oreopoulos, L. , & Platnick, S. (2008). Radiative susceptibility of cloudy atmospheres to droplet number perturbations: 2. Global analysis from modis. Journal of Geophysical Research: Atmospheres, 113(D14). 10.1029/2007JD009655

[rog20214-bib-0212] Painemal, D. , & Zuidema, P. (2013). The first aerosol indirect effect quantified through airborne remote sensing during vocals‐rex. Atmospheric Chemistry and Physics, 13(2), 917–931. 10.5194/acp-13-917-2013

[rog20214-bib-0213] Peers, F. , Waquet, F. , Cornet, C. , Dubuisson, P. , Ducos, F. , Goloub, P. , Szczap, F. , Tanré, D. , & Thieuleux, F. (2015). Absorption of aerosols above clouds from polder/parasol measurements and estimation of their direct radiative effect. Atmospheric Chemistry and Physics, 15(8), 4179–4196. 10.5194/acp-15-4179-2015

[rog20214-bib-0214] Penner, J. (2019). Soot, sulfate, dust and the climate—Three ways through the fog. Nature, 570, 158–159. 10.1038/d41586-019-01791-6 31186564

[rog20214-bib-0215] Penner, J. , M. Andreae , H. Annegarn , L. Barrie , J. Feichter , D. Hegg , A. Jayaraman , R. Leaitch , D. Murphy , J. Nganga , and G. Pitari (2001). Aerosols, their direct and indirect effects, in Climate Change 2001: Working group I: The Scientific Basis, edited by Houghton J., Ding Y., Griggs D., Noguer M., van der Linden P., Dai X., Maskell K., and Johnson C., chap. 5, pp. 289–348, Cambridge University Press, Cambridge, United Kingdom and New York, NY, USA.

[rog20214-bib-0216] Penner, J. E. , Chen, Y. , Wang, M. , & Liu, X. (2009). Possible influence of anthropogenic aerosols on cirrus clouds and anthropogenic forcing. Atmospheric Chemistry and Physics, 9(3), 879–896. 10.5194/acp-9-879-2009

[rog20214-bib-0217] Penner, J. E. , Xu, L. , & Wang, M. (2011). Satellite methods underestimate indirect climate forcing by aerosols. P. Natl. Acad. Sci. USA, 108, 13,404 10.1073/pnas.1018526108 PMC3158199 21808047

[rog20214-bib-0218] Penner, J. E. , Zhang, S. Y. , & Chuang, C. C. (2003). Soot and smoke aerosol may not warm climate. Journal of Geophysical Research – Atmospheres, 108(D21). 10.1029/2003JD003409

[rog20214-bib-0219] Penner, J. E. , Zhou, C. , Garnier, A. , & Mitchell, D. L. (2018). Anthropogenic aerosol indirect effects in cirrus clouds. Journal of Geophysical Research: Atmospheres, 123(20), 11,652–11,677. 10.1029/2018JD029204 PMC6360521 30775191

[rog20214-bib-0220] Petersik, P. , Salzmann, M. , Kretzschmar, J. , Cherian, R. , Mewes, D. , & Quaas, J. (2018). Subgrid‐scale variability of clear‐sky relative humidity and forcing by aerosol‐radiation interactions in an atmosphere model. Atmospheric Chemistry and Physics, 18, 8589–8599. 10.5194/acp-18-8589-2018

[rog20214-bib-0221] Pincus, R. , & Baker, M. (1994). Effects of precipitation on the albedo susceptibility of clouds in the marine boundary layer. Nature, 372, 250.

[rog20214-bib-0222] Pincus, R. , & Mauritsen, T. (2017). Committed warming inferred from observations. Nature Climate Change, 7, 652–655.

[rog20214-bib-0223] Platnick, S. , Meyer, K. G. , King, M. D. , Wind, G. , Amarasinghe, N. , Marchant, B. , Arnold, G. T. , Zhang, Z. , Hubanks, P. A. , Holz, R. E. , Yang, P. , Ridgway, W. L. , & Riedi, J. (2017). The MODIS cloud optical and microphysical products: Collection 6 updates and examples from Terra and Aqua. IEEE Transactions on Geoscience and Remote Sensing, 55(1), 502–525. 10.1109/TGRS.2016.2610522 29657349 PMC5896565

[rog20214-bib-0224] Platnick, S. , & Twomey, S. (1994). Determining the susceptibility of cloud albedo to changes in droplet concentration with the advanced very high resolution radiometer. Journal of Applied Meteorology, 33(3), 334–347. 10.1175/1520-0450(1994)033<0334:DTSOCA>2.0.CO;2

[rog20214-bib-0225] Possner, A. , Wang, H. , Wood, R. , Caldeira, K. , & Ackerman, T. P. (2018). The efficacy of aerosol–cloud radiative perturbations from near‐surface emissions in deep open‐cell stratocumuli. Atmospheric Chemistry and Physics, 18(23), 17,475–17,488. 10.5194/acp-18-17475-2018

[rog20214-bib-0226] Quaas, J. , Boucher, O. , Bellouin, N. , & Kinne, S. (2008). Satellite‐based estimate of the direct and indirect aerosol climate forcing. Journal of Geophysical Research, 113, 05204 10.1029/2007JD008962

[rog20214-bib-1014] Quaas, J. , Boucher, O. , & Lohmann, U. (2006). Constraining the total aerosol indirect effect in the LMDZ and ECHAM4 GCMs using MODIS satellite data. Atmospheric Chemistry and Physics, 6, 947–955.

[rog20214-bib-0227] Quaas, J. , Ming, Y. , Menon, S. , Takemura, T. , Wang, M. , Penner, J. , Gettelman, A. , Lohmann, U. , Bellouin, N. , Boucher, O. , Sayer, A. , Thomas, G. , McComiskey, A. , Feingold, G. , Hoose, C. , Kristjánsson, J. , Liu, X. , Balkanski, Y. , Donner, L. , Ginoux, P. , Stier, P. , Grandey, B. , Feichter, J. , Sednev, I. , Bauer, S. , Koch, D. , Grainger, R. , Kirkevåg, A. , Iversen, T. , Seland, Ø. , Easter, R. , Ghan, S. , Rasch, P. , Morrison, H. , Lamarque, J.‐F. , Iacono, M. , Kinne, S. , & Schulz, M. (2009). Aerosol indirect effects—General circulation model intercomparison and evaluation with satellite data. Atmospheric Chemistry and Physics, 9, 8697–8717. 10.5194/acp-9-8697-2009

[rog20214-bib-0228] Quaas, J. , Stevens, B. , Stier, P. , & Lohmann, U. (2010). Interpreting the cloud cover—Aerosol optical depth relationship found in satellite data using a general circulation model. Atmospheric Chemistry and Physics, 10(13), 6129–6135. 10.5194/acp-10-6129-2010

[rog20214-bib-0229] Ramanathan, V. (1975). Greenhouse effect due to chlorofluorocarbons: Climatic implications. Science, 190(4209), 50–52. 10.1126/science.190.4209.50

[rog20214-bib-0230] Randles, C. A. , Kinne, S. , Myhre, G. , Schulz, M. , Stier, P. , Fischer, J. , Doppler, L. , Highwood, E. , Ryder, C. , Harris, B. , Huttunen, J. , Ma, Y. , Pinker, R. T. , Mayer, B. , Neubauer, D. , Hitzenberger, R. , Oreopoulos, L. , Lee, D. , Pitari, G. , Genova, G. D. , Quaas, J. , Rose, F. G. , Kato, S. , Rumbold, S. T. , Vardavas, I. , Hatzianastassiou, N. , Matsoukas, C. , Yu, H. , Zhang, F. , Zhang, H. , & Lu, P. (2013). Intercomparison of shortwave radiative transfer schemes in global aerosol modeling: Results from the AeroCom Radiative Transfer Experiment. Atmospheric Chemistry and Physics, 13(5), 2347–2379. 10.5194/acp-13-2347-2013

[rog20214-bib-0231] Rap, A. , Scott, C. E. , Spracklen, D. V. , Bellouin, N. , Forster, P. M. , Carslaw, K. S. , Schmidt, A. , & Mann, G. (2013). Natural aerosol direct and indirect radiative effects. Geophysical Research Letters, 40(12), 3297–3301. 10.1002/grl.50441

[rog20214-bib-0232] Regayre, L. A. , Johnson, J. S. , Yoshioka, M. , Pringle, K. J. , Sexton, D. M. H. , Booth, B. B. B. , Lee, L. A. , Bellouin, N. , & Carslaw, K. S. (2018). Aerosol and physical atmosphere model parameters are both important sources of uncertainty in aerosol ERF. Atmospheric Chemistry and Physics, 18(13), 9975–10,006. 10.5194/acp-18-9975-2018

[rog20214-bib-0233] Richardson, T. B. , Forster, P. M. , Andrews, T. , & Parker, D. J. (2016). Understanding the rapid precipitation response to co2 and aerosol forcing on a regional scale. Journal of Climate, 29(2), 583–594. 10.1175/JCLI-D-15-0174.1

[rog20214-bib-0234] Rosenfeld, D. (2006). Aerosol‐cloud interactions control of Earth radiation and latent heat release budgets. Space Science Reviews, 125, 149 10.1007/s11214-006-9053-6

[rog20214-bib-0235] Rosenfeld, D. , Lohmann, U. , Raga, G. , O'Dowd, C. , Kulmala, M. , Fuzzi, S. , Reissell, A. , & Andreae, M. (2008). Flood or drought: How do aerosols affect precipitation? Science, 321, 1309 10.1126/science.1160606 18772428

[rog20214-bib-0236] Rosenfeld, D. , & Ulbrich, C. W. (2003). Cloud microphysical properties, processes, and rainfall estimation opportunities. Meteorological Monographs, 52, 237–258. 10.1175/0065-9401(2003)030<0237:CMPPAR>2.0.CO;2

[rog20214-bib-0237] Rotstayn, L. D. , Collier, M. A. , Shindell, D. T. , & Boucher, O. (2015). Why does aerosol forcing control historical global‐mean surface temperature change in CMIP5 models? Journal of Climate, 28, 6608–6625. 10.1175/JCLI-D-14-00712.1

[rog20214-bib-0238] Samset, B. H. , Myhre, G. , Herber, A. , Kondo, Y. , Li, S.‐M. , Moteki, N. , Koike, M. , Oshima, N. , Schwarz, J. P. , Balkanski, Y. , Bauer, S. E. , Bellouin, N. , Berntsen, T. K. , Bian, H. , Chin, M. , Diehl, T. , Easter, R. C. , Ghan, S. J. , Iversen, T. , Kirkevåg, A. , Lamarque, J.‐F. , Lin, G. , Liu, X. , Penner, J. E. , Schulz, M. , Seland, Ø. , Skeie, R. B. , Stier, P. , Takemura, T. , Tsigaridis, K. , & Zhang, K. (2014). Modelled black carbon radiative forcing and atmospheric lifetime in aerocom phase ii constrained by aircraft observations. Atmospheric Chemistry and Physics, 14, 12,465–12,477. 10.5194/acp-14-12465-2014

[rog20214-bib-0239] Sandu, I. , Brenguier, J.‐L. , Geoffroy, O. , Thouron, O. , & Masson, V. (2008). Aerosol impacts on the diurnal cycle of marine stratocumulus. Journal of the Atmospheric Sciences, 65(8), 2705–2718. 10.1175/2008JAS2451.1

[rog20214-bib-0240] Sato, Y. , Goto, D. , Michibata, T. , Suzuki, K. , Takemura, T. , Tomita, H. , & Nakajima, T. (2018). Aerosol effects on cloud water amounts were successfully simulated by a global cloud‐system resolving model. Nature Communications, 9(1). 10.1038/s41467-018-03379-6 PMC5841301 29515125

[rog20214-bib-0241] Satoh, M. , Noda, A. T. , Seiki, T. , Chen, Y.‐W. , Kodama, C. , Yamada, Y. , Kuba, N. , & Sato, Y. (2018). Toward reduction of the uncertainties in climate sensitivity due to cloud processes using a global non‐hydrostatic atmospheric model. Progress in Earth and Planetary Science, 5(1), 67 10.1186/s40645-018-0226-1

[rog20214-bib-0242] Schimel, D. , D. Alves , I. Entling , M. Heimann , F. Joos , D. Raynaud , T. Wigley , M. Prather , R. Derwent , D. Ehhalt , P. Fraser , E. Sanhueza , X. Zhou , P. Jonas , R. Charlson , H. Rohde , S. Sadasivan , K. P. Shine , Y. Fouquart , V. Ramaswamy , S. Solomon , J. Srinivasan , D. Albritton , I. Isaksen , M. Lal , and D. Wuebbles (1996), Radiative forcing of climate change, in Climate Change 1995: The science of climate change. Contribution of Working Group I to the Second Assessment Report of the Intergovernmental Panel on Climate Change, edited by Houghton J. T., Meiro Filho L. G., Callander B. A., Harris N., Kattenburg A., and Maskell K., chap. 2, pp. 69–131, Cambridge University Press, Cambridge, United Kingdom and New York, NY, USA.

[rog20214-bib-0243] Schmidt, J. , Ansmann, A. , Bühl, J. , & Wandinger, U. (2015). Strong aerosol–cloud interaction in altocumulus during updraft periods: Lidar observations over central Europe. Atmospheric Chemistry and Physics, 15, 10,687–10,700. 10.5194/acp-15-10687-2015

[rog20214-bib-0244] Schulz, M. , Textor, C. , Kinne, S. , Balkanski, Y. , Bauer, S. , Berntsen, T. , Berglen, T. , Boucher, O. , Dentener, F. , Guibert, S. , Isaksen, I. S. A. , Iversen, T. , Koch, D. , Kirkevåg, A. , Liu, X. , Montanaro, V. , Myhre, G. , Penner, J. E. , Pitari, G. , Reddy, S. , Seland, Ø. , Stier, P. , & Takemura, T. (2006). Radiative forcing by aerosols as derived from the aerocom present‐day and pre‐industrial simulations. Atmospheric Chemistry and Physics, 6, 5225–5246. 10.5194/acp-6-5225-2006

[rog20214-bib-0245] Schwartz, S. E. (2012). Determination of Earth's transient and equilibrium climate sensitivities from observations over the twentieth century: Strong dependence on assumed forcing. Surveys in Geophysics, 33(3), 745–777. 10.1007/s10712-012-9180-4

[rog20214-bib-0246] Schwartz, S. E. (2018). Unrealized global temperature increase: Implications of current uncertainties. Journal of Geophysical Research, 123, 3462–3482. 10.1002/2017JD028121

[rog20214-bib-0247] Schwartz, S. E. , & Andreae, M. O. (1996). Uncertainty in climate change caused by aerosols. Science, 272, 1121–1122.

[rog20214-bib-0248] Seifert, A. , Heus, T. , Pincus, R. , & Stevens, B. (2015). Large‐eddy simulation of the transient and near‐equilibrium behavior of precipitating shallow convection. Journal of Advances in Modeling Earth Systems, 7(4), 1918–1937. 10.1002/2015MS000489

[rog20214-bib-1015] Seinfeld, J. H. , Bretherton, C. , Carslaw, K. S. , Coe, H. , DeMott, P. J. , & Dunlea, E. J. (2016). Wood, R. Improving our fundamental understanding of the role of aerosol‐cloud interactions in the climate system. Proceedings of the National Academy of Sciences, 113, 5781–5790. 10.1073/pnas.1514043113 PMC4889348 27222566

[rog20214-bib-0249] Sekiguchi, M. , Nakajima, T. , Suzuki, K. , Kawamoto, K. , Higurashi, A. , Rosenfeld, D. , Sano, I. , & Mukai, S. (2003). A study of the direct and indirect effects of aerosols using global satellite data sets of aerosol and cloud parameters. Journal of Geophysical Research, 108(D22), 4699 10.1029/2002JD003359

[rog20214-bib-0250] Sena, E. T. , McComiskey, A. , & Feingold, G. (2016). A long‐term study of aerosol–cloud interactions and their radiative effect at the Southern Great Plains using ground‐based measurements. Atmospheric Chemistry and Physics, 16(17), 11,301–11,318. 10.5194/acp-16-11301-2016

[rog20214-bib-0251] Sherwood, S. C. , Bony, S. , Boucher, O. , Bretherton, C. , Forster, P. M. , Gregory, J. M. , & Stevens, B. (2015). Adjustments in the forcing‐feedback framework for understanding climate change. Bull. Amer. Meteor. Soc., 96, 217–228. 10.1175/BAMS-D-13-00167.1

[rog20214-bib-0252] Shine, K. P. , Cook, J. , Highwood, E. J. , & Joshi, M. M. (2003). An alternative to radiative forcing for estimating the relative importance of climate change mechanisms. Geophysical Research Letters, 30(20). 10.1029/2003GL018141

[rog20214-bib-0253] Skeie, R. B. , Berntsen, T. , Aldrin, M. , Holden, M. , & Myhre, G. (2018). Climate sensitivity estimates—Sensitivity to radiative forcing time series and observational data. Earth System Dynamics, 9(2), 879–894. 10.5194/esd-9-879-2018

[rog20214-bib-0254] Small, J. D. , Chuang, P. Y. , Feingold, G. , & Jiang, H. (2009). Can aerosol decrease cloud lifetime? Geophysical Research Letters, 36 10.1029/2009GL038888

[rog20214-bib-0255] Small, J. D. , Jiang, J. H. , Su, H. , & Zhai, C. (2011). Relationship between aerosol and cloud fraction over Australia. Geophysical Research Letters, 38, 23,802 10.1029/2011GL049404

[rog20214-bib-0256] Smirnov, A. , Holben, B. , Eck, T. , Dubovik, O. , & Slutsker, I. (2000). Cloud‐screening and quality control algorithms for the aeronet database. Remote Sensing of Environment, 73(3), 337–349. 10.1016/S0034-4257(00)00109-7

[rog20214-bib-0257] Smirnov, A. , Holben, B. , Slutsker, I. , Giles, D. , McClain, C. R. , Eck, T. , Sakerin, S. , Macke, A. , Croot, P. , Zibordi, G. , Quinn, P. , Sciare, J. , Kinne, S. , Harvey, M. , Smyth, T. , Piketh, S. , Zielinski, T. , Proshutinsky, A. , Goes, J. , Nelson, N. , Larouche, P. , Radionov, V. , Goloub, P. , Moorthy, K. K. , Matarrese, R. , Robertson, E. , & Jourdin, F. (2009). Maritime aerosol network as a component of aerosol robotic network. Journal of Geophysical Research, 114(D6), D06,204 10.1029/2008JD011257

[rog20214-bib-0258] Smith, C. J. , Kramer, R. J. , Myhre, G. , Forster, P. M. , Soden, B. J. , Andrews, T. , Boucher, O. , Faluvegi, G. , Fläschner, D. , Hodnebrog, Ø. , Kasoar, M. , Kharin, V. , Kirkevåg, A. , Lamarque, J.‐F. , Mülmenstädt, J. , Olivié, D. , Richardson, T. , Samset, B. H. , Shindell, D. , Stier, P. , Takemura, T. , Voulgarakis, A. , & Watson‐Parris, D. (2018). Understanding rapid adjustments to diverse forcing agents. Geophysical Research Letters, 45, 12,023–12,031. 10.1029/2018GL079826 PMC6334512 30686845

[rog20214-bib-0259] Sorooshian, A. , Feingold, G. , Lebsock, M. , Jiang, H. , & Stephens, G. (2009). On the precipitation susceptibility of clouds to aerosol perturbations. Geophysical Research Letters, 36, L13803 10.1029/2009GL038993

[rog20214-bib-0260] Sourdeval, O. , Gryspeerdt, E. , Krämer, M. , Goren, T. , Delanoë, J. , Afchine, A. , Hemmer, F. , & Quaas, J. (2018). Ice crystal number concentration estimates from lidar–radar satellite remote sensing—Part 1: Method and evaluation. Atmospheric Chemistry and Physics, 18(19), 14,327–14,350. 10.5194/acp-18-14327-2018

[rog20214-bib-0261] Stanelle, T. , Bey, I. , Raddatz, T. , Reick, C. , & Tegen, I. (2014). Anthropogenically induced changes in twentieth century mineral dust burden and the associated impact on radiative forcing. Journal of Geophysical Research – Atmospheres, 119(23), 13,526–13,546. 10.1002/2014JD022062

[rog20214-bib-0262] Stephens, G. L. , L'Ecuyer, T. , Forbes, R. , Gettlemen, A. , Golaz, J.‐C. , Bodas‐Salcedo, A. , Suzuki, K. , Gabriel, P. , & Haynes, J. (2010). Dreary state of precipitation in global models. Journal of Geophysical Research, 115(D24), D24,211 10.1029/2010JD014532

[rog20214-bib-0263] Stevens, B. (2015). Rethinking the lower bound on aerosol radiative forcing. Journal of Climate, 28(12), 4794–4819. 10.1175/JCLI-D-14-00656.1

[rog20214-bib-0264] Stevens, B. , & Feingold, G. (2009). Untangling aerosol effects on clouds and precipitation in a buffered system. Nature, 461(7264), 607–613. 10.1038/nature08281 19794487

[rog20214-bib-0265] Stevens, B. , & Fiedler, S. (2017). Reply to “comment on ‘rethinking the lower bound on aerosol radiative forcing’”. Journal of Climate, 30(16), 6585–6589. 10.1175/JCLI-D-17-0034.1

[rog20214-bib-0266] Stier, P. (2016). Limitations of passive remote sensing to constrain global cloud condensation nuclei. Atmospheric Chemistry and Physics, 16(10), 6595–6607. 10.5194/acp-16-6595-2016

[rog20214-bib-0267] Stier, P. , Feichter, J. , Kloster, S. , Vignati, E. , & Wilson, J. (2006). Emission‐induced nonlinearities in the global aerosol system: Results from the ECHAM5‐HAM aerosol‐climate model. Journal of Climate, 19(16), 3845–3862. 10.1175/JCLI3772.1

[rog20214-bib-0268] Stier, P. , Schutgens, N. A. J. , Bellouin, N. , Bian, H. , Boucher, O. , Chin, M. , Ghan, S. , Huneeus, N. , Kinne, S. , Lin, G. , Ma, X. , Myhre, G. , Penner, J. E. , Randles, C. A. , Samset, B. , Schulz, M. , Takemura, T. , Yu, F. , Yu, H. , & Zhou, C. (2013). Host model uncertainties in aerosol radiative forcing estimates: Results from the AeroCom Prescribed intercomparison study. Atmospheric Chemistry and Physics, 13(6), 3245–3270. 10.5194/acp-13-3245-2013

[rog20214-bib-0269] Stjern, C. W. , Samset, B. H. , Myhre, G. , Forster, P. M. , Hodnebrog, T. , Andrews, Ø. , Boucher, O. , Faluvegi, G. , Iversen, T. , Kasoar, M. , Kharin, V. , Kirkevåg, A. , Lamarque, J.‐F. , Olivié, D. , Richardson, T. , Shawki, D. , Shindell, D. , Smith, C. J. , Takemura, T. , & Voulgarakis, A. (2017). Rapid adjustments cause weak surface temperature response to increased black carbon concentrations. Journal of Geophysical Research – Atmospheres, 122(21), 11,462–11,481. 10.1002/2017JD027326 PMC7241673 32441705

[rog20214-bib-0270] Storelvmo, T. (2017). Aerosol effects on climate via mixed‐phase and ice clouds. Annual Review of Earth and Planetary Sciences, 45(1), 199–222. 10.1146/annurev-earth-060115-012240

[rog20214-bib-0271] Storelvmo, T. , Heede, U. , Leirvik, T. , Phillips, P. , Arndt, P. , & Wild, M. (2018). Lethargic response to aerosol emissions in current climate models. Geophysical Research Letters, 45(18), 9814–9823. 10.1029/2018GL078298

[rog20214-bib-0272] Storelvmo, T. , Kristjánsson, J. E. , Lohmann, U. , Iversen, T. , Kirkevåg, A. , & Seland, Ø. (2008). Modeling of the Wegener‐Bergeron‐Findeisen process—Implications for aerosol indirect effects. Environmental Research Letters, 3(4), 045,001 10.1088/1748-9326/3/4/045001

[rog20214-bib-0273] Ström, J. , & Ohlsson, S. (1998). In situ measurements of enhanced crystal number densities in cirrus clouds caused by aircraft exhaust. Journal of Geophysical Research, 103(D10), 1355–1361.

[rog20214-bib-0274] Suzuki, K. , Stephens, G. L. , & Lebsock, M. D. (2013). Aerosol effect on the warm rain formation process: Satellite observations and modeling. Journal of Geophysical Research, 118(1), 170–184. 10.1002/jgrd.50043

[rog20214-bib-0275] Takemura, T. , Nozawa, T. , Emori, S. , Nakajima, T. Y. , & Nakajima, T. (2005). Simulation of climate response to aerosol direct and indirect effects with aerosol transport‐radiation model. Journal of Geophysical Research – Atmospheres, 110(D2), D02202 10.1029/2004JD005029

[rog20214-bib-0276] Tan, I. , Storelvmo, T. , & Choi, Y.‐S. (2014). Spaceborne lidar observations of the ice‐nucleating potential of dust, polluted dust, and smoke aerosols in mixed‐phase clouds. Journal of Geophysical Research, 119(11), 6653–6665. 10.1002/2013JD021333

[rog20214-bib-0277] Taylor, K. E. , Stouffer, R. J. , & Meehl, G. A. (2012). An overview of CMIP5 and the experiment design. Bull. Amer. Meteorol. Soc., 93(4), 485–498. 10.1175/BAMS-D-11-00094.1

[rog20214-bib-0278] Tegen, I. , Werner, M. , Harrison, S. P. , & Kohfeld, K. E. (2004). Relative importance of climate and land use in determining present and future global soil dust emission. Geophysical Research Letters, 31, n/a 10.1029/2003GL019216

[rog20214-bib-0279] Textor, C. , Schulz, M. , Guibert, S. , Kinne, S. , Balkanski, Y. , Bauer, S. , Berntsen, T. , Berglen, T. , Boucher, O. , Chin, M. , Dentener, F. , Diehl, T. , Easter, R. , Feichter, H. , Fillmore, D. , Ghan, S. , Ginoux, P. , Gong, S. , Grini, A. , Hendricks, J. , Horowitz, L. , Huang, P. , Isaksen, I. , Iversen, I. , Kloster, S. , Koch, D. , Kirkevåg, A. , Kristjansson, J. E. , Krol, M. , Lauer, A. , Lamarque, J. F. , Liu, X. , Montanaro, V. , Myhre, G. , Penner, J. , Pitari, G. , Reddy, S. , Seland, Ø. , Stier, P. , Takemura, T. , & Tie, X. (2006). Analysis and quantification of the diversities of aerosol life cycles within aerocom. Atmospheric Chemistry and Physics, 6(7), 1777–1813. 10.5194/acp-6-1777-2006

[rog20214-bib-2222] Thomas, G. E. , Carboni, E. , Sayer, A. M. , Poulsen, C. A. , Siddans, R. , & Grainger, R. G. (2009). Oxford‐RAL Aerosol and Cloud (ORAC): Aerosol retrievals from satellite radiometers In Kokhanovsky A., de Leeuw G., (Eds.). Aerosol Remote Sensing over Land (pp. 193–225). Berlin/Heidelberg, Germany: Springer.

[rog20214-bib-0280] Thornton, J. A. , Virts, K. S. , Holzworth, R. H. , & Mitchell, T. P. (2017). Lightning enhancement over major oceanic shipping lanes. Geophysical Research Letters, 44, 9102–9111. 10.1002/2017GL074982

[rog20214-bib-0281] Toll, V. , Christensen, M. , Gassó, S. , & Bellouin, N. (2017). Volcano and ship tracks indicate excessive aerosol‐induced cloud water increases in a climate model. Geophysical Research Letters, 44, 12,492–12,500. 10.1002/2017GL075280 PMC5921053 29713108

[rog20214-bib-0282] Toll, V. , Christensen, M. , Quaas, J. , & Bellouin, N. (2019). Weak average liquid‐cloud‐water response to anthropogenic aerosols. Nature, 572(7767), 51–55. 10.1038/s41586-019-1423-9 31367029

[rog20214-bib-0283] Twohy, C. H. , Petters, M. D. , Snider, J. R. , Stevens, B. , Tahnk, W. , Wetzel, M. , Russell, L. , & Burnet, F. (2005). Evaluation of the aerosol indirect effect in marine stratocumulus clouds: Droplet number, size, liquid water path, and radiative impact. Journal of Geophysical Research: Atmospheres, 110(D8), D08203 10.1029/2004JD005116

[rog20214-bib-0284] Twomey, S. (1959). The nuclei of natural cloud formation part ii: The supersaturation in natural clouds and the variation of cloud droplet concentration. Geofisica Pura e Applicata, 43(1), 243–249. 10.1007/BF01993560

[rog20214-bib-0285] Twomey, S. (1974). Pollution and the planetary albedo. Atmospheric Environment, 8(12), 1251–1256. 10.1016/0004-6981(74)90004-3

[rog20214-bib-0286] Twomey, S. (1977). The influence of pollution on the shortwave albedo of clouds. Journal of the Atmospheric Sciences, 34(7), 1149–1152. 10.1175/1520-0469(1977)034<1149:TIOPOT>2.0.CO;2

[rog20214-bib-0287] Twomey, S. , & Warner, J. (1967). Comparison of measurements of cloud droplets and cloud nuclei. Journal of the Atmospheric Sciences, 24(6), 702–703. 10.1175/1520-0469(1967)024<0702:COMOCD>2.0.CO;2

[rog20214-bib-0180] van Marle, M. J. E. , Kloster, S. , Magi, B. I. , Marlon, J. R. , Daniau, A.‐L. , Field, R. D. , Arneth, A. , Forrest, M. , Hantson, S. , Kehrwald, N. M. , Knorr, W. , Lasslop, G. , Li, F. , Mangeon, S. , Yue, C. , Kaiser, J. W. , & van der Werf, G. R. (2017). Historic global biomass burning emissions for CMIP6 (BB4CMIP) based on merging satellite observations with proxies and fire models (1750–2015). Geoscientific Model Development, 10(9), 3329–3357. 10.5194/gmd-10-3329-2017

[rog20214-bib-0288] Várnai, T. , & Marshak, A. (2009). MODIS observations of enhanced clear sky reflectance near clouds. Geophysical Research Letters, 36, 6807 10.1029/2008GL037089

[rog20214-bib-0289] Vergara‐Temprado, J. , Holden, M. A. , Orton, T. R. , O'Sullivan, D. , Umo, N. S. , Browse, J. , Reddington, C. , Baeza‐Romero, M. T. , Jones, J. M. , Lea‐Langton, A. , Williams, A. , Carslaw, K. S. , & Murray, B. J. (2018). Is black carbon an unimportant ice‐nucleating particle in mixed‐phase clouds? Journal of Geophysical Research: Atmospheres, 123, 4273–4283. 10.1002/2017JD027831 PMC6001433 29938147

[rog20214-bib-0290] Vergara‐Temprado, J. , Miltenberger, A. K. , Furtado, K. , Grosvenor, D. P. , Shipway, B. J. , Hill, A. A. , Wilkinson, J. M. , Field, P. R. , Murray, B. J. , & Carslaw, K. S. (2018). Strong control of southern ocean cloud reflectivity by ice‐nucleating particles. Proceedings of the National Academy of Sciences, 115(11), 2687–2692. 10.1073/pnas.1721627115 PMC5856555 29490918

[rog20214-bib-0291] Wang, M. , Ghan, S. , Liu, X. , Yer, T. S. , Zhang, K. , Morrison, H. , Ovchinnikov, M. , Easter, R. , Marchand, R. , Chand, D. , Qian, Y. , & Penner, J. E. (2012). Constraining cloud lifetime effects of aerosols using a‐train satellite observations. Geophysical Research Letters, 39, 15,709 10.1029/2012GL052204

[rog20214-bib-0292] Wang, R. , Andrews, E. , Balkanski, Y. , Boucher, O. , Myhre, G. , Samset, B. H. , Schulz, M. , Schuster, G. L. , Valari, M. , & Tao, S. (2018). Spatial representativeness error in the ground‐level observation networks for black carbon radiation absorption. Geophysical Research Letters, 45, 2106–2114. 10.1002/2017GL076817 29937603 PMC5993241

[rog20214-bib-0293] Wang, S. , Wang, Q. , & Feingold, G. (2003). Turbulence, condensation, and liquid water transport in numerically simulated nonprecipitating stratocumulus clouds. Journal of the Atmospheric Sciences, 60(2), 262–278. 10.1175/1520-0469(2003)060<0262:TCALWT>2.0.CO;2

[rog20214-bib-0294] Wang, X. , Heald, C. L. , Ridley, D. A. , Schwarz, J. P. , Spackman, J. R. , Perring, A. E. , Coe, H. , Liu, D. , & Clarke, A. D. (2014). Exploiting simultaneous observational constraints on mass and absorption to estimate the global direct radiative forcing of black carbon and brown carbon. Atmospheric Chemistry and Physics, 14(20), 10,989–11,010. 10.5194/acp-14-10989-2014

[rog20214-bib-0295] Wang, X. , Liu, J. , Che, H. , Ji, F. , & Liu, J. (2018). Spatial and temporal evolution of natural and anthropogenic dust events over northern China. Scientific Reports, 8(1), 2141 10.1038/s41598-018-20382-5 29391425 PMC5795005

[rog20214-bib-0296] Warren, S. G. , & Wiscombe, W. J. (1980). A model for the spectral albedo of snow II: Snow containing atmospheric aerosols. Journal of the Atmospheric Sciences, 37(12), 2734–2745. 10.1175/1520-0469(1980)037<2734:AMFTSA>2.0.CO;2

[rog20214-bib-0298] Watson‐Parris, D. , Schutgens, N. , Winker, D. , Burton, S. P. , Ferrare, R. A. , & Stier, P. (2018). On the limits of CALIOP for constraining modeled free tropospheric aerosol. Geophysical Research Letters, 45, 9260–9266. 10.1029/2018GL078195

[rog20214-bib-0299] Webb, N. P. , & Pierre, C. (2018). Quantifying anthropogenic dust emissions. Earth's Future, 6, 286–295. 10.1002/2017EF000766

[rog20214-bib-0300] Werner, F. , Ditas, F. , Siebert, H. , Simmel, M. , Wehner, B. , Pilewskie, P. , Schmeissner, T. , Shaw, R. A. , Hartmann, S. , Wex, H. , Roberts, G. C. , & Wendisch, M. (2014). Twomey effect observed from collocated microphysical and remote sensing measurements over shallow cumulus. Journal of Geophysical Research – Atmospheres, 119(3), 1534–1545. 10.1002/2013JD020131

[rog20214-bib-0301] White, B. , Gryspeerdt, E. , Stier, P. , Morrison, H. , Thompson, G. , & Kipling, Z. (2017). Uncertainty from the choice of microphysics scheme in convection‐permitting models significantly exceeds aerosol effects. Atmospheric Chemistry and Physics, 17(19), 12,145–12,175. 10.5194/acp-17-12145-2017

[rog20214-bib-0302] Wielicki, B. A. , Barkstrom, B. R. , Harrison, E. F. , Lee, R. B. , Louis Smith, G. , & Cooper, J. E. (1996). Clouds and the Earth's Radiant Energy System (CERES): An earth observing system experiment. Bull. Amer. Meteorol. Soc., 77(5), 853–868. 10.1175/1520-0477(1996)077<0853:CATERE>2.0.CO;2

[rog20214-bib-0303] Wilcox, E. M. (2012). Direct and semi‐direct radiative forcing of smoke aerosols over clouds. Atmospheric Chemistry and Physics, 12(1), 139–149. 10.5194/acp-12-139-2012

[rog20214-bib-0304] Williams, E. , Rosenfeld, D. , Madden, N. , Gerlach, J. , Gears, N. , Atkinson, L. , Dunnemann, N. , Frostrom, G. , Antonio, M. , Biazon, B. , Camargo, R. , Franca, H. , Gomes, A. , Lima, M. , Machado, R. , Manhaes, S. , Nachtigall, L. , Piva, H. , Quintiliano, W. , Machado, L. , Artaxo, P. , Roberts, G. , Renno, N. , Blakeslee, R. , Bailey, J. , Boccippio, D. , Betts, A. , Wolff, D. , Roy, B. , Halverson, J. , Rickenbach, T. , Fuentes, J. , & Avelino, E. (2002). Contrasting convective regimes over the amazon: Implications for cloud electrification. Journal of Geophysical Research, 107(D20), 8082 10.1029/2001JD000380

[rog20214-bib-0305] Winker, D. M. , Tackett, J. L. , Getzewich, B. J. , Liu, Z. , Vaughan, M. A. , & Rogers, R. R. (2013). The global 3‐D distribution of tropospheric aerosols as characterized by CALIOP. Atmospheric Chemistry and Physics, 13(6), 3345–3361. 10.5194/acp-13-3345-2013

[rog20214-bib-0306] WMO (2017), World Meteorological Organization Cloud Atlas, cloudatlas.wmo.int.

[rog20214-bib-0307] Wood, R. , K.‐T. O. , Bretherton, C. S. , Mohrmann, J. , Albrecht, B. A. , Zuidema, P. , Ghate, V. , Schwartz, C. , Eloranta, E. , Glienke, S. , Shaw, R. A. , Fugal, J. , & Minnis, P. (2018). Ultraclean layers and optically thin clouds in the stratocumulus‐to‐cumulus transition. part i: Observations. Journal of the Atmospheric Sciences, 75(5), 1631–1652. 10.1175/JAS-D-17-0213.1

[rog20214-bib-1016] Wood, R. , Leon, D. , Lebsock, M. , Snider, J. , & Clarke, A. D. (2012). Precipitation driving of droplet concentration variability in marine low clouds. Journal of Geophysical Research, 117, D19210 10.1029/2012JD018305

[rog20214-bib-0308] Xue, H. , & Feingold, G. (2006). Large‐eddy simulations of trade wind cumuli: Investigation of aerosol indirect effects. Journal of the Atmospheric Sciences, 63(6), 1605–1622. 10.1175/JAS3706.1

[rog20214-bib-0309] Yamaguchi, T. , Feingold, G. , Kazil, J. , & McComiskey, A. (2015). Stratocumulus to cumulus transition in the presence of elevated smoke layers. Geophysical Research Letters, 42, 10,478–10,485. 10.1002/2015GL066544

[rog20214-bib-0310] Yoshioka, M. , Regayre, L. , Pringle, K. , Johnson, J. , Mann, G. , Partridge, D. , Sexton, D. , Schutgens, N. , Stier, P. , Kipling, Z. , Bellouin, N. , Browse, J. , Booth, B. , Johnson, C. , Johnson, B. T. , Mollard, J. , Lee, L. , & Carslaw, K. (2019). Ensembles of global climate model variants designed for the quantification and constraint of uncertainty in aerosols and their radiative forcing. Journal of Advances in Modeling Earth Systems, 2019MS001628 10.1029/2019MS001628

[rog20214-bib-0311] Yuan, T. , Remer, L. A. , Pickering, K. E. , & Yu, H. (2011). Observational evidence of aerosol enhancement of lightning activity and convective invigoration. Geophysical Research Letters, 38, L04701 10.1029/2010GL046052

[rog20214-bib-0312] Yuan, T. , Remer, L. A. , & Yu, H. (2011). Microphysical, macrophysical and radiative signatures of volcanic aerosols in trade wind cumulus observed by the A‐Train. Atmospheric Chemistry and Physics, 11(14), 7119–7132. 10.5194/acp-11-7119-2011

[rog20214-bib-0313] Zelinka, M. D. , Andrews, T. , Forster, P. M. , & Taylor, K. E. (2014). Quantifying components of aerosol‐cloud‐radiation interactions in climate models. Journal of Geophysical Research, 119(12), 7599–7615. 10.1002/2014JD021710

[rog20214-bib-0314] Zerefos, C. S. , Tetsis, P. , Kazantzidis, A. , Amiridis, V. , Zerefos, S. C. , Luterbacher, J. , Eleftheratos, K. , Gerasopoulos, E. , Kazadzis, S. , & Papayannis, A. (2014). Further evidence of important environmental information content in red‐to‐green ratios as depicted in paintings by great masters. Atmospheric Chemistry and Physics, 14(6), 2987–3015. 10.5194/acp-14-2987-2014

[rog20214-bib-0315] Zhang, H. , Zhao, S. , Wang, Z. , Zhang, X. , & Song, L. (2016). The updated effective radiative forcing of major anthropogenic aerosols and their effects on global climate at present and in the future. International Journal of Climatology, 36(12), 4029–4044. 10.1002/joc.4613

[rog20214-bib-0316] Zhang, J. , Reid, J. , & Holben, B. (2005). An analysis of potential cloud artifacts in MODIS over ocean aerosol optical thickness products. Geophysical Research Letters, 32, L15,803 10.1029/2005GL023254

[rog20214-bib-0317] Zhao, B. , Liou, K.‐N. , Gu, Y. , Jiang, J. H. , Li, Q. , Fu, R. , Huang, L. , Liu, X. , Shi, X. , Su, H. , & He, C. (2018). Impact of aerosols on ice crystal size. Atmospheric Chemistry and Physics, 18(2), 1065–1078. 10.5194/acp-18-1065-2018 31534446 PMC6750036

[rog20214-bib-0318] Zhou, C. , & Penner, J. E. (2014). Aircraft soot indirect effect on large‐scale cirrus clouds: Is the indirect forcing by aircraft soot positive or negative? Journal of Geophysical Research: Atmospheres, 119(19), 11,303–11,320. 10.1002/2014JD021914

[rog20214-bib-0319] Zhu, J. , Penner, J. E. , Yu, F. , Sillman, S. , Andreae, M. O. , & Coe, H. (2019). Decrease in radiative forcing by organic aerosol nucleation, climate, and land use change. Nature Communications, 10(1), 423 10.1038/s41467-019-08407-7 PMC6345905 30679429

[rog20214-bib-1017] Zuidema, P. , Redemann, J. , Haywood, J. , Wood, R. , Piketh, S. , Hipondoka, M. , & Formenti, P. (2016). Smoke and clouds above the southeast atlantic: Upcoming field campaigns probe absorbing aerosol's impact on climate. Bull. American Meteorological Society, 97(7), 1131–1135. 10.1175/BAMS-D-15-00082.1

